# Dinitrogen cleavage and hydrogenation to ammonia with a uranium complex

**DOI:** 10.1093/nsr/nwac144

**Published:** 2022-07-22

**Authors:** Xiaoqing Xin, Iskander Douair, Yue Zhao, Shuao Wang, Laurent Maron, Congqing Zhu

**Affiliations:** State Key Laboratory of Coordination Chemistry, Jiangsu Key Laboratory of Advanced Organic Materials, School of Chemistry and Chemical Engineering, Nanjing University, Nanjing 210023, China; School of Medicine and Holistic Integrative Medicine, Nanjing University of Chinese Medicine, Nanjing 210023, China; LPCNO, CNRS and INSA, Université Paul Sabatier, Toulouse 31077, France; State Key Laboratory of Coordination Chemistry, Jiangsu Key Laboratory of Advanced Organic Materials, School of Chemistry and Chemical Engineering, Nanjing University, Nanjing 210023, China; State Key Laboratory of Radiation Medicine and Protection, School for Radiological and Interdisciplinary Sciences (RAD-X) and Collaborative Innovation Center of Radiation Medicine of Jiangsu Higher Education Institutions, Soochow University, Suzhou 215123, China; LPCNO, CNRS and INSA, Université Paul Sabatier, Toulouse 31077, France; State Key Laboratory of Coordination Chemistry, Jiangsu Key Laboratory of Advanced Organic Materials, School of Chemistry and Chemical Engineering, Nanjing University, Nanjing 210023, China

**Keywords:** N_2_ activation, NH_3_ formation, depleted uranium, uranium azide, actinide coordination chemistry

## Abstract

The Haber–Bosch process produces ammonia (NH_3_) from dinitrogen (N_2_) and dihydrogen (H_2_), but requires high temperature and pressure. Before iron-based catalysts were exploited in the current industrial Haber–Bosch process, uranium-based materials served as effective catalysts for production of NH_3_ from N_2_. Although some molecular uranium complexes are known to be capable of combining with N_2_, further hydrogenation with H_2_ forming NH_3_ has not been reported to date. Here, we describe the first example of N_2_ cleavage and hydrogenation with H_2_ to NH_3_ with a molecular uranium complex. The N_2_ cleavage product contains three uranium centers that are bridged by three imido *μ*_2_-NH ligands and one nitrido *μ*_3_-N ligand. Labeling experiments with ^15^N demonstrate that the nitrido ligand in the product originates from N_2_. Reaction of the N_2_-cleaved complex with H_2_ or H^+^ forms NH_3_ under mild conditions. A synthetic cycle has been established by the reaction of the N_2_-cleaved complex with trimethylsilyl chloride. The isolation of this trinuclear imido-nitrido product implies that a multi-metallic uranium assembly plays an important role in the activation of N_2_.

## Teaser

The example of N_2_ activation and direct hydrogenation with H_2_ to NH_3_ was achieved by a molecular uranium complex under mild conditions.

## INTRODUCTION

**Figure 1.**
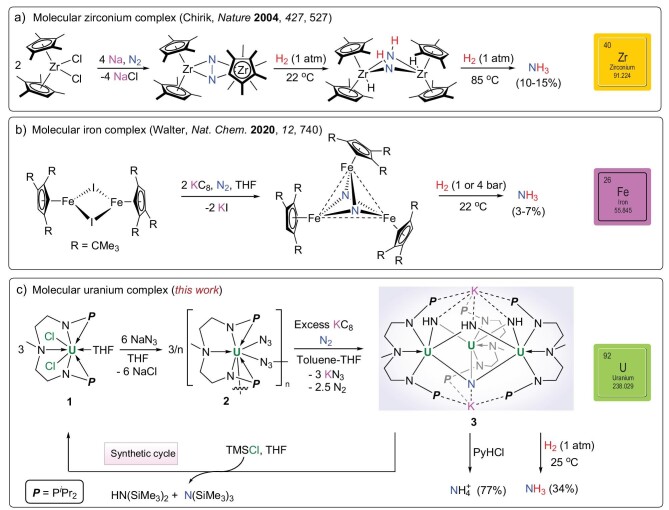
NH_3_ formation from N_2_ and H_2_ promoted by molecular complexes. (a) Hydrogenation and cleavage of N_2_ to NH_3_ with a Zr complex [13]. (b) N_2_ reduction and hydrogenation to NH_3_ with Fe complexes [30]. (c) N_2_ cleavage and hydrogenation to NH_3_ with a U complex (this work). N_2_ activation or cleavage occurs under reducing conditions with Na or KC_8_. The N atoms originating from N_2_ are shown in blue.

Fixation, activation and functionalization of dinitrogen (N_2_) are challenging issues due to the presence of the unreactive and non-polar N≡N triple bond in N_2_ [1–7]. Both nitrogenase enzymes and the industrial Haber–Bosch process for N_2_ fixation use transition metals to catalyze this challenging transformation [8,9], and both catalytic N_2_ fixation by transition metals and subsequent protonation to ammonia (NH_3_) have been extensively studied in recent decades [10–33]. However, examples of the hydrogenation of N_2_-activated products with hydrogen (H_2_) to form NH_3_, similar to the industrial Haber–Bosch process, remain uncommon [13,30]. Chirik and co-workers found that an N_2_-derived zirconium nitride complex reacts with H_2_ at 85^o^C to give NH_3_ in 10%–15% yield (Fig. 1a) [13]. Recently, Walter and co-workers found that the NH_3_ was formed in 3%–7% yield from N_2_ and H_2_ mediated by a molecular tri-iron species (Fig. 1b) [30].

Although uranium-based materials were known to be effective catalysts for NH_3_ production from N_2_ before use of the iron-based catalysts in the current industrial Haber–Bosch process [34], only a few examples of well-defined uranium species capable of transforming N_2_ into NH_3_ were known for more than a century and all NH_3_ formation processes required protonation with acid [35–38]. For instance, Mazzanti and co-workers reported a multi-metallic nitride-bridged diuranium(III) complex, [K_3_{[U(OSi(O*^t^*Bu)_3_)_3_]_2_(*μ*-N)}], which can convert N_2_ to NH_3_ after protonation with acid [35]. Arnold *et al.* recently reported thorium or uranium dinuclear metallacycles M_2_(*m*TP)_2_ (M = U, Th; *m*TP = [{2-(OC_6_H_2_-*^t^*Bu-2, Me-4)_2_CH}-C_6_H_4_-1,3]^4−^), which can mediate the reduction and protonation of N_2_ to NH_3_ in the presence of potassium graphite (KC_8_) [36]. However, examples of the direct hydrogenation of an N_2_-activated product with H_2_, forming NH_3_ by a molecular uranium complex, have not been reported.

Herein we report an example of NH_3_ formation from the hydrogenation of an N_2_-cleaved complex with H_2_ in a molecular uranium system (Fig. 1c). The conversion of the N_2_-cleaved product to silylamine was also achieved, thus a synthetic cycle has been established with the generation of the uranium precursor.

## RESULTS AND DISCUSSION

### Synthesis, characterization and transformation

Treatment of a uranium complex **1** [37] with 2 equiv. of NaN_3_ at room temperature (RT) in tetrahydrofuran (THF) resulted in the formation of **2**, which was isolated in 72% yield as a crystalline product (Fig. 1c). The ^1^H nuclear magnetic resonance (^1^H NMR) spectrum of **2** has 11 peaks between +69.64 and –70.48 ppm (Fig. S1), indicating an unsymmetrical structure. The Fourier-transform infrared (FT-IR) spectrum of **2** exhibits two strong azide stretching bands at 2094 cm^−1^ and 2150 cm^−1^ (Fig. S2), which are characteristic of actinide-bound terminal and bridged azide ligands, respectively [39–42].

**Figure 2.**
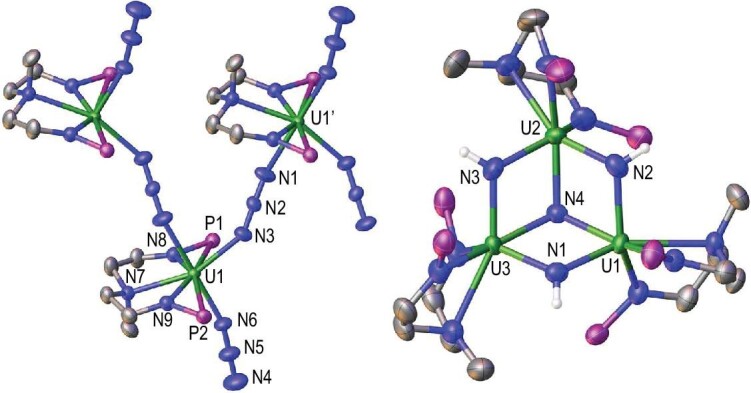
Molecular structures of **2** (left) and **3** (right) (50% probability). Hydrogen atoms (except for the NH protons in **3**), isopropyl moieties in P*^i^*Pr_2_ and two K^+^ counter ions in **3** are omitted for clarity. Selected distances (Å) and angles for **2**: U1–N3 2.447(3), U1–N6 2.292(3), U1–N7 2.584(3), U1–N8 2.240(3), U1–N9 2.220(3), N1–N2 1.159(4), N2–N3 1.169(4), N4–N5 1.145(5), N5–N6 1.181(4); N1–N2–N3 179.0(5)^0^, N4–N5–N6 176.8(5)^0^. For **3**: U1–N1 2.193(5), U1–N2 2.222(6), U2–N2 2.193(5), U2–N3 2.231(5), U3–N3 2.188(6), U3–N1 2.222(5), U1–N4 2.218(5), U2–N4 2.211(5), U3–N4 2.204(5); U1–N2–U2 106.8(2)^0^, U2–N3–U3 106.5(2)^0^, U3–N1–U1 106.7(2)^0^, U1–N4–U2 106.3(2)^0^, U1–N4–U3 106.4(2)^0^, U2–N4–U3 106.7(2)^0^.

The solid-state molecular structure of **2**, confirmed by single-crystal X-ray diffraction, is a novel one-dimensional molecular chain connected through 1,3-end-on bridged azides (Fig. 2). The U–N_azide_ distances between the uranium and bridged azide are 2.447(3) Å for U1–N3 and 2.471(4) Å for U1’–N1, which are clearly longer than the terminal U–N_azide_ distance (U1–N6 2.292(3) Å) but are similar to the corresponding distances in previously reported multi-metallic uranium complexes with bridged azide ligands [40,43,44]. The N1–N2 and N2–N3 distances are 1.159(4) and 1.169(4) Å, respectively, suggesting that a delocalized ^−^N = N^+^ = N^−^ resonance form is predominant in this 1,3-end-on bridged azide. However, the terminal azide shows a more localized form with N4–N5 and N5–N6 distances of 1.145(5) and 1.181(5) Å, respectively, suggesting weak activation of this terminal azide.

Uranium azides are well known as synthetic precursors of terminal uranium nitride species in photochemical or redox processes [39,40,45–48]. We first investigated the photolysis and thermolysis of **2**, but the products were unidentifiable. However, when **2** was reduced with an excess of KC_8_ in a toluene-THF mixed solvent under an N_2_ atmosphere, the color of the solution changed immediately from yellow-brown to dark brown. After working up this mixture, a crystalline complex, [{[U{N(CH_3_)(CH_2_CH_2_NP*^i^*Pr_2_)_2_}(*μ*-NH)]_3_(*μ*-N)}K_2_] (**3**) was isolated in 31% yield (Fig. 1c). The ^1^H NMR spectrum of **3** has signals between +73.69 and –33.22 ppm (Fig. S4). Attempts to increase the yield of **3** were unsuccessful even though it was the isolated product from the *in-situ* reaction of **2** with KC_8_ (Fig. S6). Dibenzyl was detected by gas chromatography-mass spectrometric (GC-MS) analysis of the reaction mixture (Fig. S7), which is consistent with the failure to obtain **3** when the reaction was conducted in the absence of toluene. Complex **3** could also be formed by the reaction of **2** with an excess of KC_8_ in THF in the presence of 1 equiv. of 9,10-dihydroanthracene (Fig. S8). These results suggest that the aromatic hydrogen source plays an important role in the formation of **3**. The presence of N–H groups in **3** was confirmed by the formation of HN(SiMe_3_)_2_ in the reaction of **3** with TMSCl (*vide infra*) and broad absorption at 3450 cm^−1^ in the FT-IR spectrum of **3** (Fig. S3) [39,40].

The molecular structure of **3** was also established by X-ray crystallography (Fig. 2). The salient feature of this structure is the presence of three U centers bridged by three imido *μ*-NH ligands and one nitrido *μ*_3_-N ligand. The six U–N_imido_ distances fall in the range of 2.188−2.231 Å and are comparable with the bridged U–N_imido_ distances in the range of 2.10–2.55 Å as reported previously [36,49,50]. These near-equivalent U–N_imido_ distances suggest that it is not a U–N=U bonding interaction but a U–NH–U unit by the comparison with reported analogues [50,51]. The three U–N_nitrido_ distances that range from 2.204 to 2.218 Å are comparable to those found in U(IV)/U(VI) tetrauranium nitride clusters (2.183(7)−2.319(7) Å) [52] and slightly longer than those found in a *μ*_3_-N nitride uranium complex (2.138−2.157 Å) [53]. Despite their bridging nature, the U–N_imido_ and U–N_nitrido_ distances in **3** remain slightly shorter than the sum of the single bond covalent radii of U and N (2.41 Å) [54]. Although similar trinuclear imido-nitride structures with transition metals have been described by the groups of Roesky [55], Yélamos [56] and Hou [20], complex **3** represents the first example of a trinuclear uranium species with both imido and nitrido ligands.

Treatment of **3** in THF solution with excess pyridine hydrochloride (PyHCl) produces NH_4_Cl in 77% yield (Fig. 1c). To confirm the source of the nitrido ligand in **3**, we reduced **2** with KC_8_ under 1 atm of ^15^N_2_. After the acidification of the ^15^N-labeled product (**3-^15^N**) with excess pyridine hydrochloride, a triplet resonance (δ = 7.42 ppm, *J*_NH_ = 52 Hz, assigned to NH_4_Cl) and a doublet resonance (δ = 7.42 ppm, *J*_NH_ = 72 Hz, assigned to ^15^NH_4_Cl) were observed in a ratio of ∼3:0.5 in its ^1^H NMR spectrum in deuterated dimethyl sulfoxide (Fig. S13) [57,58]. Exposing the THF solution of **3** to 1 atm of ^15^N_2_ for 2 days does not reveal any exchange between **3** and ^15^N_2_ (Fig. S14). Thus the generation of ^15^NH_4_Cl reveals that the ^15^N_2_ cleavage was involved in the formation of **3**. The ideal ratio for ^14^NH_4_^+^:^15^NH_4_^+^ is 3:1 (*vide infra*), and the lower ratio of ^15^NH_4_Cl is presumably due to the ^14^N_2_ generated *in situ* by the reduction of U–^14^N_3_ units, which possibly were unable to escape from the solvent cage even when the reduction of **2** took place under the ^15^N_2_ atmosphere. This result is consistent with the fact that complex **3** can also be prepared by the reduction of **2** with KC_8_ under Ar, albeit with a lower crystalline yield (16%). The ^1^H NMR spectrum recorded *in situ* of the reaction of **2** with KC_8_ under dynamic vacuum shows that no **3** was formed in the absence of N_2_ (Fig. S15). These results all demonstrate that the nitrido ligand in **3** originates from N_2_. Fixing and activation of N_2_ derived from the reduction of metal azides is very rare for either *d*- or *f*-block metals. Liddle and co-workers isolated a uranium(v)–bis(imido)–dinitrogen complex, [U(BIPM^TMS^)(NAd)_2_(μ-η^1^:η^1^-N_2_)(Li-2,2,2-cryptand)] by reacting a uranium-carbene species with an organoazide under an N_2_ or Ar atmosphere [59]. The N–N bond length of the coordinated N_2_ in this species (1.139(9) Å) is only slightly elongated over the N–N length in the free N_2_ molecule (1.0975 Å). Consequently, **3** represents the first example of N_2_ scission in a metal-azide reduction.

Direct hydrogenation of the N_2_-activated product with H_2_ under mild conditions was examined. Complex **3** was treated with H_2_ at atmospheric pressure and RT (Fig. 1c). The *in-situ*^1^H NMR and ^31^P{^1^H} NMR spectra of the reaction mixture show that **3** was consumed within 8 h and the free ligand was formed in the reaction (Figs S16 and S17). The formation of NH_3_ in 34% yield was identified by the formation of NH_4_Cl after treating the volatile products with an excess of PyHCl (Fig. S18). The NH_3_ formation in this hydrogenation process was further confirmed by the reaction of **3** with 1 atm D_2_ at RT. However, the signals for ND_3_ and NHD_2_ in the ^2^H NMR spectrum are very similar and could not be distinguished (Fig. S19) [30,60,61]. This process is the first example of NH_3_ production by the hydrogenation of an N_2_-activated product with H_2_ or D_2_ in a uranium system. The reaction of the ^15^N-labeled product (**3-^15^N**) with H_2_ generates ^14^NH_3_ and ^15^NH_3_ in a ratio of ∼3:0.5 (Fig. S20), which shows that the three imido groups and one nitrido group in **3** were all converted to NH_3_. The hydrogenation of transition metal nitrido complexes with H_2_ has been reported previously [62–64]. Reaction of **3** overnight with an excess of Me_3_SiCl (TMSCl) at RT afforded the uranium precursor (**1**, 46% yield) and N-containing products (HN(SiMe_3_)_2_ and N(SiMe_3_)_3_) (Figs S21 and S22). Both H^14^N(SiMe_3_)_2_ and ^15^N(SiMe_3_)_3_ were observed in the reaction of complex **3-^15^N** with Me_3_SiCl (Fig. S23), which further confirmed that complex **3** contains both imido and nitrido groups and the source of the nitrido group is ^15^N_2_. A synthetic cycle has thus been established allowing reuse of the uranium-containing precursor (Fig. 1c). Attempts to construct N–C bonds from the reactions of complex **3** with electrophilic reagents, such as MeOTf, 2,4,6-(CH_3_)_3_C_6_H_2_NCO and *p*-CH_3_C_6_H_4_COCl, have been unsuccessful.

**Figure 3.**
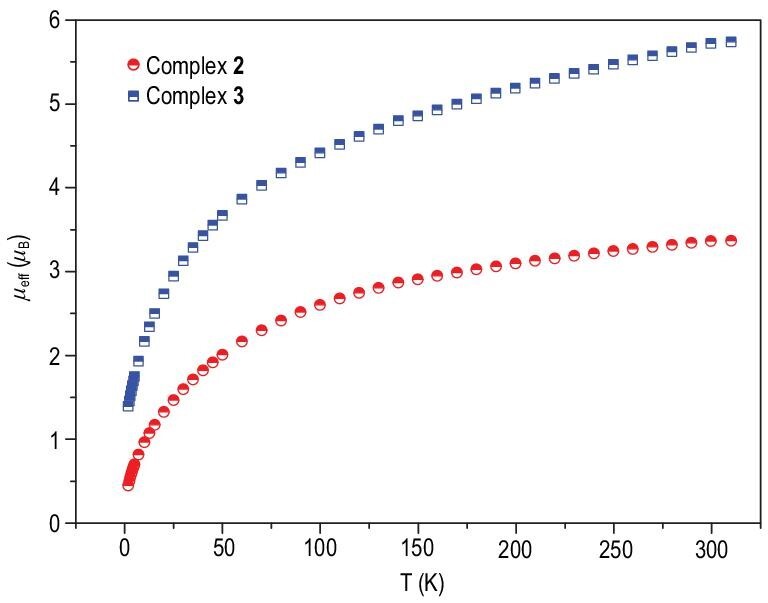
Variable-temperature effective magnetic moment data of **2** (with one U ion) and **3** (with three U ions) under a magnetic field of 1000 Oe.

The oxidation state of the uranium center in **2** is +IV, which was confirmed by the variable-temperature magnetic data determined by a super-conducting quantum interference device (SQUID) in the solid state (Fig. 3). Typically, the magnetic moment of a U(IV) ion approaches zero at low temperatures due to a 5*f*^2^ singlet ground state. The magnetic moment of **2** is 3.37 *μ*_B_ at 300 K and smoothly decreases to 0.45 *μ*_B_ at 1.8 K, then approaches zero. The magnitude of *μ*_eff_ and temperature dependence of **2** are consistent with a U(IV) center, which is a magnetic singlet at low temperatures [40,45,65–67]. Formally, **3** contains two U(IV) centers and one U(V) center. The measured magnetic moment at 300 K for **3**, which contains three U ions, is 5.72 *μ*_B_. It exhibits significant temperature dependence, decreasing steadily to 1.39 *μ*_B_ at 1.8 K, which is characteristic of U(V) 5*f*^1^ complexes. To further investigate the electronic structure of uranium ions, the ultraviolet-visible-near infrared (UV-Vis-NIR) absorption spectra of **2** and **3** were recorded in THF at RT (Fig. S26). The spectrum of **2** shows moderate absorption in the 300–450 nm range, while the spectrum of **3** exhibits a significantly more intense absorption than that of **2** over the entire visible and NIR region. Both **2** and **3** exhibit several weak absorption peaks (ϵ < 50 and 200 M^−1^ cm^−1^ for **2** and **3**, respectively) in the NIR region, which is characteristic of *f-f* transitions involving the 5*f*^1^ and 5*f*^2^ electronic configuration [59,68–70]. X-ray photoelectron spectroscopy (XPS) was undertaken to investigate the probable oxidation state of uranium in **3** (Fig. S27). The binding energy for U-4*f*_7/2_ in the XPS of **3** was determined to be 380.52 eV, which is in the range of the binding energies for U(IV) and U(V) species [71,72]. These results are consistent with the assignment of U(IV)/U(IV)/U(V) to **3** and the overall charge of this cluster is balanced with two K^+^ ions.

### Theoretical studies

**Figure 4.**
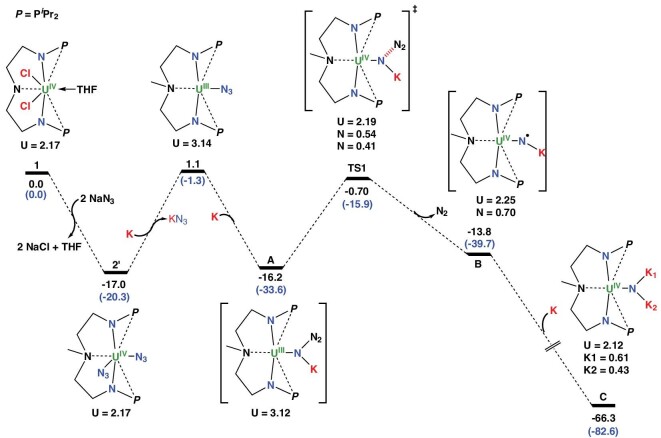
Computed enthalpy profile (in kcal mol^−1^) for the reduction of **2′** to a dianionic uranium–nitride intermediate **C**. Gibbs free energies are given in parentheses. The unpaired spin density numbers are also indicated for all complexes.

To further investigate the conversion of **1** to **3**, density functional theory (DFT) calculations were carried out using the B3PW91 function that has been proven to be reliable in dealing with such systems [36–38]. The formation of NH_3_ from **3** and H_2_ is out of scope computationally since locating transition states for complexes as large as **3** is not yet possible even with supercomputers. Dispersion corrections were considered and appeared to be small in this case (Figs S30 and S31). The Gibbs free energies were calculated and differed from the enthalpy barriers by only 1.0 kcal mol^−1^. Experimentally, **2** is a coordination polymer in the solid state that could not be computationally modeled. However, the formation of a monomer (**2′**) was predicted to be favorable by 17.0 kcal mol^−1^ (Fig. 4). The main geometrical features of **2** were correctly reproduced in the computed monomeric form **2′**. For instance, the U–N distances are 2.26 Å vs. 2.24 Å (exp.) and the N–N distances are 1.21/1.15 Å vs. 1.18/1.15 Å (exp.). The unpaired spin density is 2.17, consistent with a U(IV) center. Reduction of the diazide **2′** is predicted to be almost athermic (0.8 kcal mol^−1^) and is assisted by the coordination of a K^+^ ion to form the monoazide **A** [40,46]. In the absence of potassium coordination, the reduction is endothermic by 28.6 kcal mol^−1^ (Fig. S31). The reduction of the U(IV) center is highlighted by the unpaired spin density value of 3.12 at the uranium center of **A**, which is consistent with a U(III) system. This monoazide **A** stabilized by a K^+^ can lose N_2_, forming a nitride **B**. A potassium-mediated N_2_-release transition state (**TS1**) has been identified and the associated energy barrier is 15.5 kcal mol^−1^, suggesting a facile process.

Two facts reveal the significance of the potassium. First, the release of N_2_ from the monomeric form of **2′** in the absence of K was shown to imply an activation barrier of 46.7 kcal mol^−1^ (28.6 kcal mol^−1^ from the uncapped monoazide complex, Fig. S31). Interestingly, the N–N bond cleavage implies a single electron transfer from the uranium center to the azide ligand. In **TS1**, the unpaired spin density appears to be distributed between U (2.19), consistent with a U(IV) system, and the two terminal nitrogen atoms of the azide ligand (0.54 for the nitride and 0.42 for the N_2_), which are stabilized by the potassium cation. Following the intrinsic reaction coordinate, **TS1** releases N_2_, forming a nitrido-type intermediate **B**, whose formation from the diazide **2′** is slightly endothermic by 3.2 kcal mol^−1^ (Fig. 4). Intermediate **B** could be better described as a U(IV)–(nitride radical)–K(I) complex since the unpaired spin density is distributed between U (2.25) and N (0.70). The presence of an unpaired electron in the nitride indicates that this intermediate is fairly unstable and will react further with potassium to yield a dianionic uranium–nitride (**C**) stabilized by two potassium counterions (–66.3 kcal mol^−1^ from **1**, –49.3 kcal mol**^−^**^1^ from the diazide **2′**). The intermediate **C** is also interesting because the unpaired spin density indicates that the potassium does not reduce the metal but reduces the nitride, yielding a U(IV)–N(-III)–2K(I) species (unpaired spin density of 2.12 on U, and 0.61 and 0.43 on the two K ions). However, attempts to isolate this terminal uranium nitride intermediate at low temperatures and/or with diminished levels of KC_8_ were unsuccessful.

**Figure 5.**
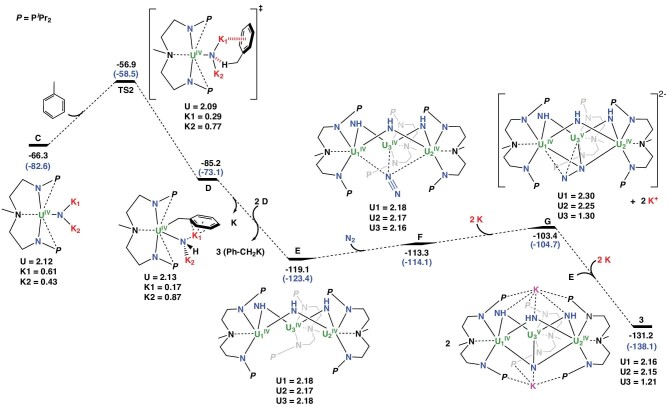
Computed enthalpy profile (in kcal mol^−1^) for the formation of **3** from the uranium-nitride **C**. Gibbs free energies are given in parentheses. The unpaired spin density numbers are also indicated for all complexes.

Intermediate **C** appears to be sufficiently nucleophilic to abstract a hydrogen from the solvent. The relevant **TS2** was located and the associated barrier is 9.4 kcal mol^−1^, indicating a facile reaction (Fig. 5). The charge on the hydrogen (+0.22) shows that this reaction is a proton transfer rather than a hydrogen atom transfer (HAT) reaction and this is possibly due to the presence of a U(IV)–N(-III) unit in **C**. At the **TS2**, the assistance from the potassium is again crucial since an observed interaction between one potassium and the phenyl ring of the toluene allowed the proton transfer. Following the intrinsic reaction coordinate, this leads to the formation of a PhCH_2_K adduct to the U(IV)–imido **D** (–85.2 kcal mol^−1^ from the entrance channel), which can easily trimerize with loss of PhCH_2_K to form **E** (–119.1 kcal mol^−1^). This trimetallic tri-imido species **E** can bind N_2_ to form **F** (–113.3 kcal mol^−1^). Although low-valent U species has a stronger interaction with N_2_, examples of U(IV) or even U(V) complexes have been reported to be capable of binding N_2_ experimentally [36,37,59]. In **F**, the three uranium centers are U(IV) with unpaired spin densities of ∼2.17 per U and the N_2_ is not reduced (N–N distance of 1.19 Å) but binds to the uranium (U–N_2_ Wiberg bond index (WBI) of 0.26). The lowest unoccupied molecular orbital (LUMO) of **F** is a bonding interaction between the uranium centers and the π* of N_2_ (Fig. S32).

**Figure 6.**
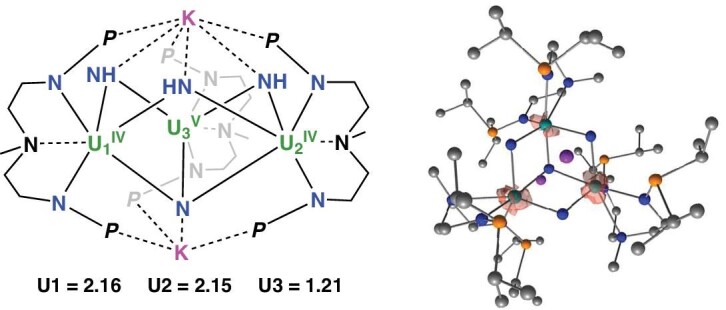
Unpaired spin density plot of complex **3**.

Finally, the reduction of **F** yields the species **G** (–103.4 kcal mol^−1^), in which the N_2_ moiety is triply reduced and has an N–N distance of 1.3 Å with an unpaired spin density of 0.8 on N_2_. This implies that one uranium center has been oxidized, as evidenced by the unpaired spin densities of U (Fig. 5). **G** is thus halfway to **3**, and **3** is formed upon coordination of a second **E**. This coordination was investigated computationally and leads readily to the formation of two molecules of complex **3**. However, the size of the system with six uranium centers precluded frequency calculations and only the thermodynamic of the transformation from **3** to **1** could be obtained. The formation of **3** from **1** is exothermic by –131.2 kcal mol^−1^. The optimized geometry of **3** agrees well with the experimental observations. The U–N distances, for example, are correctly reproduced (between 2.17 and 2.24 Å vs. between 2.17 and 2.23 Å exp.). Scrutiny of the unpaired spin density in **3** supports determination of two U(IV) and one U(V) in this species (Fig. 6), although the possibility of having three equivalent uranium centers with a formal oxidation state of +4.33 is also consistent with the computed charges and symmetry of the system. Upon comparison of the uranium oxidation states found for the nitride and imido complexes, the formation of **3** implies a N≡N bond cleavage via trimetallic uranium synergy, but only a single electron oxidation of one uranium center, which is surprising at first glance. However, the other electrons used for the reduction are held by the potassium.

## CONCLUSIONS

In summary, we report the first example of N_2_ cleavage and hydrogenation by H_2_, forming NH_3_ in a uranium system. The N_2_-cleaved product was formed by the reduction of a uranium azide complex, which has not been observed previously for the reduction of any *d*-block or *f*-block metal azides. The detailed mechanism of N_2_ activation by multi-metallic synergy was revealed by DFT calculations, which show that the formation of **3** from **1** is likely to involve the formation of transient U(IV)–nitride and U(IV)–imido complexes. The reduction of N_2_ yields the final trinuclear imido-nitrido uranium complex, which can be protonated by H_2_ or H^+^ to form NH_3_ under mild conditions. The uranium precursor **1** was generated by the functionalization of **3** with TMSCl, thereby establishing a synthetic cycle. This study suggests that multiple uranium atoms can cooperate in the cleavage of the N≡N triple bond in N_2_ and that uranium species are promising materials for activation and conversion of small molecules.

## METHODS

Experiments were performed under an Ar or N_2_ atmosphere using standard Schlenk-line or glovebox techniques. Solvents were dried and degassed with a solvent purification system before use. See the Supplementary Data for detailed experimental procedures, and crystallographic and computational details.

## Data availability

The X-ray crystallographic coordinates for structures reported in this study have been deposited at the Cambridge Crystallographic Data Centre (CCDC), under deposition numbers CCDC-2021046 (**2**), 2021047 (**3**, prepared under Ar) and 2044515 (**3**, prepared under N_2_). These data can be obtained free of charge from the CCDC via www.ccdc.cam.ac.uk/data_request/cif. The data that support the findings of this study are available from the corresponding author upon request.

## Supplementary Material

nwac144_Supplemental_File
