# Supplementary material for: Dinitrogen cleavage and hydrogenation to ammonia with a uranium complex
Source: Natl Sci Rev. 2022 Jul 22;10(2):nwac144. doi: 10.1093/nsr/nwac144 (PMC10026940; doi:10.1093/nsr/nwac144)
Supplement: nwac144_Supplemental_File [file nwac144_supplemental_file.pdf]

Supplementary material

for

Dinitrogen Cleavage and Hydrogenation to Ammonia with a Uranium Complex

Xiaoqing Xin,[1,2] Iskander Douair,[3] Yue Zhao,[1] Shuao Wang,[4] Laurent Maron,[3]* and Congqing Zhu[1]*

[1] State Key Laboratory of Coordination Chemistry, Jiangsu Key Laboratory of Advanced Organic Materials, School of Chemistry and Chemical Engineering, Nanjing University, Nanjing 210023, China

[2] School of Medicine & Holistic Integrative Medicine, Nanjing University of Chinese Medicine, Nanjing 210023, China

[3] LPCNO, CNRS & INSA, Université Paul Sabatier, 31077 Toulouse, France

[4] State Key Laboratory of Radiation Medicine and Protection, School for Radiological and interdisciplinary Sciences (RAD-X) and Collaborative Innovation Center of Radiation Medicine of Jiangsu Higher Education Institutions, Soochow University, Suzhou 215123, China

*Correspondence and requests for materials should be addressed to L.M. (E-mail: laurent.maron@irsamc.ups-tlse.fr) or to C.Z. (E-mail: zcq@nju.edu.cn).

Contents

1. Experimental Procedure……………………………………………………………2

2. Supplementary Figures……………………………………………………………7

3. X-ray Crystallographic Analysis………………………………………………………22

4. Theoretical Calculations…………………………………………………………..28

5. References……………………………………………………………………………58

1. Experimental Procedure

General Procedure: All manipulations were performed under an atmosphere of argon or nitrogen using standard Schlenk techniques or a glovebox. Commercially available chemicals were purchased from TCI, Aladdin and J&K Scientific Ltd., and used as received without further purification unless otherwise stated. The solvents were obtained by passing through a Solve Purer G5 (MIKROUNA) solvent purification system and further dried over 4 Å molecular sieves. THF-d_8 were dried over Na/K and stored under an Ar or N_2 atmosphere prior to use. Nuclear magnetic resonance spectroscopy was performed using a Bruker AVIII-400, a Bruker AVIII-500 or a Bruker AVIII-600 spectrometer at room temperature (RT). The ^1H and $^{13}C\{^1H\}$ NMR chemical shifts (δ) are reported relative to tetramethylsilane, and $^{31}P\{^1H\}$ NMR chemical shifts are relative to 85% H_3PO_4. Absolute values of the coupling constants are provided in Hertz (Hz). Multiplicities are abbreviated as singlet (s), doublet (d), triplet (t), multiplet (m), and broad (br). Magnetic measurements on crystalline samples were performed using a Quantum Design SQUID VSM magnetometer from 300 to 1.8 K under an external magnetic field of 1000 Oe. The sample was added to a pre-weighed SQUID capsule in a glovebox. The capsule was then sealed, weighed, and transferred to the SQUID cavity for the magnetic measurements. All magnetic data were corrected for the diamagnetic contributions of the sample holder and of the core diamagnetism of the samples using Pascal's constant.[1] Fourier transform infrared spectra (FT-IR) were measured on a Nicolet FT-IR 170X spectrophotometer in the range of 4000-400 cm^{-1} at 25 °C using KBr plates. X-ray photoelectron spectroscopy (XPS) was recorded on an EscaLab 250Xi photoelectron spectrometer. Elemental analyses (C, H, N) were performed on a Vario EL III elemental analyzer at the Shanghai Institute of Organic Chemistry, Chinese Academy of Sciences. GC-MS experiments were performed

on Agilent 5977B. Complex **1** and KC_8 were prepared according to previously reported procedures.[2,3]

Synthesis of {[U{N(CH$_3$)(CH$_2$CH$_2$NP^{i}Pr$_2$)$_2$}(N$_3$)](*μ*-N$_3$)}$_n$] (2)

A solution of {U[N(CH$_3$)(CH$_2$CH$_2$NP^{i}Pr$_2$)$_2$](Cl)$_2$(THF)} **1** (218 mg, 0.3 mmol, 1 equiv.) in THF (2 mL) was added to a mixture of NaN_3 (39 mg, 0.6 mmol, 2 equiv.) in THF (2 mL). The mixture was stirred overnight at RT and then filtered through celite. The filtrate was dried *in vacuo* and the solid was washed three times with toluene (1.0 mL) to afford complex **2** as a yellow-brown solid (171 mg, 85%). Crystals of **2** suitable for X-ray diffraction were grown from a saturated solution in THF stored at -30 ^{o}C (145 mg, 72%). ^{1}H NMR (THF-d$_8$, 500 MHz, ppm) δ 69.64 (s, 2H, CH_2), 54.45 (s, 2H, CH_2), 54.28 (s, 2H, CH_2), 47.16 (s, 2H, CH_2), 25.25 (s, 6H, CH(CH_3)$_2$), 24.52 (s, 6H, CH(CH_3)$_2$), 24.43 (s, 6H, CH(CH_3)$_2$), 22.05 (s, 6H, CH(CH_3)$_2$), -31.96 (s, 2H, CH(CH$_3$)$_2$), -58.61 (s, 2H CH(CH$_3$)$_2$), -70.48 (s, 3H, NCH_3). ^{31}P{^{1}H} NMR (THF-d$_8$, 202 MHz, ppm) δ 64.6. FTIR *v*/cm^{-1} (KBr): 3459 (w), 3417 (w), 2969 (s), 2949 (s), 2919 (s), 2861 (s), 2712 (s), 2150 (s), 2094 (s), 1458 (s), 1361 (s), 1330 (m), 1290 (w), 1232 (w), 1192 (w), 1150 (m), 1121 (s), 1052 (w), 1016 (w), 980 (w), 951(m), 905 (m), 876 (w), 764 (s), 658 (w), 604 (w), 569 (w), 542 (w), 511 (w), 439 (w). Anal. Calcd. for $C_{17}H_{39}N_9P_2U$: C, 30.50; H, 5.87; N, 18.83. Found: C, 30.56; H, 5.91; N, 18.74.

Synthesis of [{[U{N(CH$_3$)(CH$_2$CH$_2$NP^{i}Pr$_2$)$_2$}(μ-NH)]$_3$(μ-N)}K$_2$] (3) and [{[U{N(CH$_3$)(CH$_2$CH$_2$NP^{i}Pr$_2$)$_2$}(μ-NH)]$_3$(μ-^{15}N)}K$_2$] (3-^{15}N)

A solution of complex **2** (134 mg, 0.2 mmol, 1 equiv.) in THF (2 mL) and toluene (5 drops) was added dropwise to a suspension of KC_8 (162 mg, 1.2 mmol, 6 equiv.) in THF (2 mL) under an atmosphere of argon or nitrogen. The mixture was stirred overnight at RT and then the solvents were removed under reduced pressure and the residues were extracted with toluene. The filtrate was concentrated to 1 mL and placed at RT for 48 h. Dark-brown crystals of **3** suitable for X-ray diffraction were obtained (under Ar: 20 mg, 16%; under N_2: 39 mg, 31%). Attempts to improve the crystallized yield of complex **3** were unsuccessful although it was the major product in the *in-situ* reactions. **3-^{15}N** was prepared by exposing **2** to $^{15}N_2$. A mixture of **2** (134 mg, 0.2 mmol, 1 equiv.) and KC_8 (162 mg, 1.2 mmol, 6 equiv.) in THF (4 mL) was freeze pump thaw degassed three times and exposed to an atmosphere of $^{15}N_2$ (1 atm), following the same procedure that was used with **3** to afford **3-^{15}N**. Complex **3** could be also formed by the reaction of complex **2** with excess KC_8 in THF in the presence of 9.10-dihydroanthracene. Complex **3** could not be obtained when the reaction was conducted in the absence of toluene and 9.10-dihydroanthracene. Under Ar: ^{1}H NMR (THF-d$_8$, 400 MHz, ppm) ^{1}H NMR (THF-d$_8$, 400 MHz, ppm) δ 73.69 (s, 1H), 69.71 (s, 1H), 34.38 (s, 1H), 20.96 (s, 1H), 11.29 (s, 1H), 10.49 (s, 1H), 5.40 (s, 1H), 4.97 (s, 4H), -0.01 (s, 3H), -0.37 (s, 3H), -1.66 (s, 3H), -3.48 (s, 3H), -5.65 (s, 3H), -6.58 (s, 6H), -12.02 (s, 1H), -12.67 (s, 3H), -18.59 (s, 3H), -33.22 (s, 1H). ^{31}P{^{1}H} NMR (THF-d$_8$, 162 MHz, ppm) δ 115.52, 65.87. FTIR v/cm^{-1} (KBr): 3450 (br, NH, w), 2942 (m), 2861 (m), 2806 (m), 1639 (m), 1455 (w), 1373 (w), 1245 (m), 1146 (w), 1090 (m), 986 (w), 934 (w), 875 (w), 772 (m), 627 (w), 555 (w), 514 (w), 466 (w). Anal. Calcd. for $C_{51}H_{120}K_2N_{13}P_6U_3$: C, 32.35; H, 6.39; N, 9.62. Found: C, 32.20; H, 6.33; N, 9.34.

Under N_2: ^1H NMR (THF-d_8, 400 MHz, ppm) δ 73.75 (s, 1H), 69.79 (s, 1H), 34.40 (s, 1H), 20.99 (s, 1H), 11.30 (s, 1H), 10.50 (s, 1H), 5.43 (s, 1H), 4.98 (s, 4H), 0.01 (s, 3H), -0.36 (s, 3H), -1.66 (s, 3H), -3.47 (s, 3H), -5.65 (s, 3H), -6.57 (s, 6H), -12.03 (s, 1H), -12.68 (s, 3H), -18.62 (s, 3H), -33.25 (s, 1H). $^{31}P\{^1H\}$ NMR (THF-d_8, 162 MHz, ppm) δ 116.10, 64.15. Anal. Calcd. for $C_{51}H_{120}K_2N_{13}P_6U_3$: C, 32.35; H, 6.39; N, 9.62. Found: C, 32.04; H, 6.25; N, 9.26.

Reaction of 2 with KC_8 under dynamic vacuum

A Schlenk flask was charged with a mixture of **2** (34 mg, 0.05 mmol, 1 equiv.) and KC_8 (41 mg, 0.3 mmol, 6 equiv.) in THF-d_8 (2 mL) was freeze pump thaw degassed three times and immersed in liquid nitrogen. Then the flask was connected to a Schlenk line and slowly evacuated. The mixture was removed from liquid nitrogen and warmed to RT followed by stirring for 2 h. The *in-situ* ^1H NMR spectrum (THF-d_8, 400 MHz) shows that no complex **3** was formed.

Protonation of complexes 3 and 3-^{15}N with acid

An excess of pyridine hydrochloride (15.3 mg, 0.13 mmol, 50 equiv.) was added to a brown solution of **3** (5.0 mg, 0.0026 mmol, 1 equiv.) in THF (1 ml). The mixture was stirred at RT overnight, affording a pale-yellow solution and a white precipitate. The supernatant was removed and the solid was washed three times with 1.0 mL of THF, then dried under vacuum. ^1H NMR analysis showed the presence of ammonium chloride (NH_4Cl) and the protonated ligand [$CH_3N(CH_2CH_2NHP^iPr_2)_2$]. The amount of ammonia was evaluated by quantitative ^1H NMR using dibromomethane as an internal standard. The NH_4Cl was formed by the reaction of complex **3** with PyHCl (77% yield of ammonia). The NH_4Cl and $^{15}NH_4Cl$ were formed by the reaction of **3-^{15}N** with PyHCl following the same procedure (71% yield of ammonia). ^1H NMR of NH_4Cl (DMSO-d_6, 400 MHz, ppm): δ = 7.42

(t, J = 52 Hz); ^{1}H NMR of $^{15}NH_4Cl$ (DMSO-d_6, 400 MHz, ppm): δ = 7.42 (d, J = 72 Hz).

Hydrogenation of complexes 3 and 3-^{15}N with H_2

In an NMR tube, a brown solution of **3** (5.0 mg, 0.0026 mmol) in 0.5 ml of THF-d_8 was freeze-degassed three times and then exposed to 1 atm of H_2 at RT. The NMR tube was closed and left at RT for 8 h, until the NMR spectrum showed that the starting material had disappeared. The *in-situ* ^{1}H NMR spectrum shows the presence of free ligand [$CH_3N(CH_2CH_2NHP^iPr_2)_2$]. All the volatile components were vacuum transferred into a Schlenk flask containing PyHCl (15.3 mg, 0.13 mmol) dissolved in DMSO-d_6. The amount of ammonia (34%) was evaluated by quantitative ^{1}H NMR spectrum using dibromomethane as an internal standard. The NH_4Cl was formed by the reaction of NH_3 (produced by complex **3** with H_2) with PyHCl. When complex **3-^{15}N** was used in this process, NH_4Cl and $^{15}NH_4Cl$ were observed in the ^{1}H NMR spectrum, suggesting the formation of NH_3 and $^{15}NH_3$ by the reaction of complex **3-^{15}N** with H_2.

Reaction of complex 3 with TMSCl

A brown solution of **3** (20.0 mg, 0.0104 mmol) in 0.5 ml of THF-d_8 was treated with Me_3SiCl (11.3 mg, 0.104 mmol) and stirred at RT overnight. The *in-situ* ^{1}H NMR analysis showed that complex **3** had disappeared and complex **1** was formed. The reaction mixture was filtered. The formation of $HN(SiMe_3)_2$ and $N(SiMe_3)_3$ were observed by GC-MS analysis of the filtrate. The filtrate was dried *in vacuo* and the residue was washed three times with 2 mL of hexane until the washing was colourless. The residues were extracted with toluene and dried *in vacuo* to afford complex **1** in 46% yield. The $HN(SiMe_3)_2$ and $^{15}N(SiMe_3)_3$ were observed by GC-MS analysis of the reaction of **3-^{15}N** with TMSCl following the same procedures.

2. Supplementary Figures

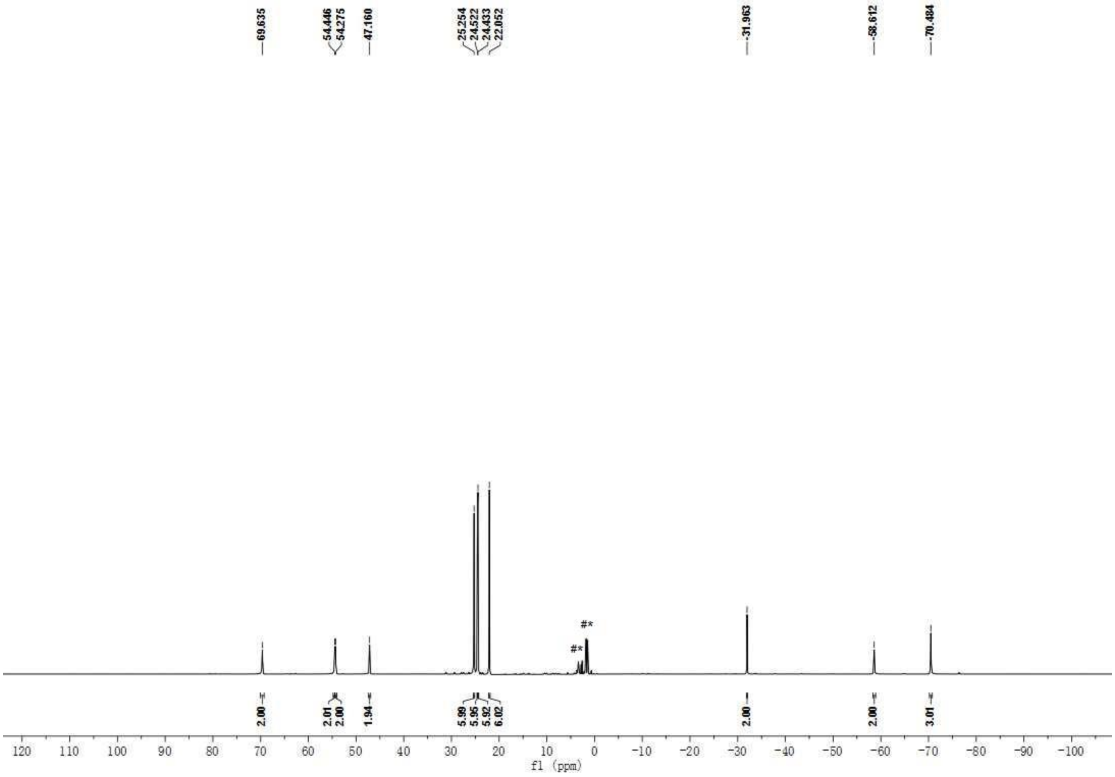

Fig. S1. The ^{1}H NMR (THF-d_8, 500 MHz) spectrum of complex **2** (# are peaks assigned to THF and * are peaks assigned to impurities in THF-d_8).

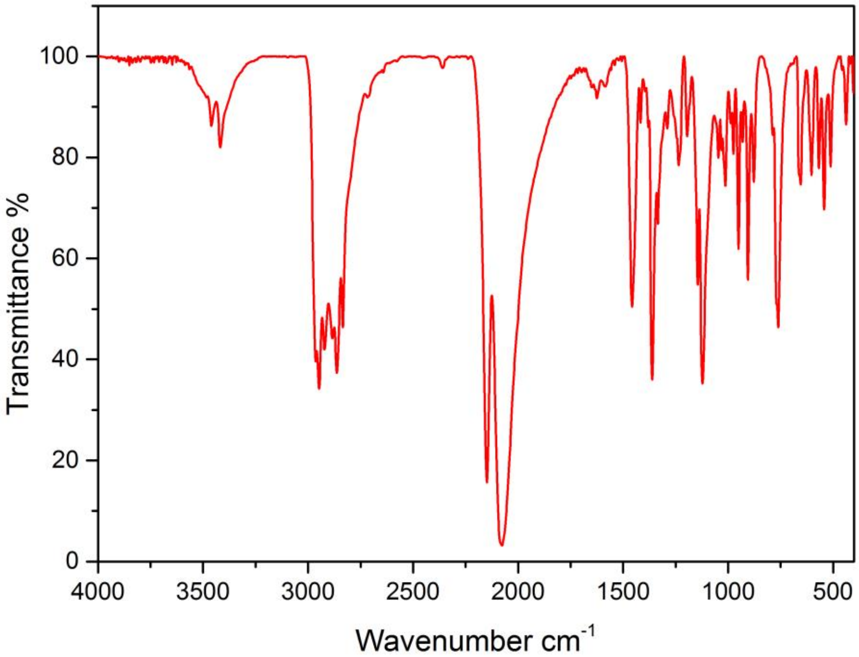

Fig. S2. FT-IR spectrum of complex **2**.

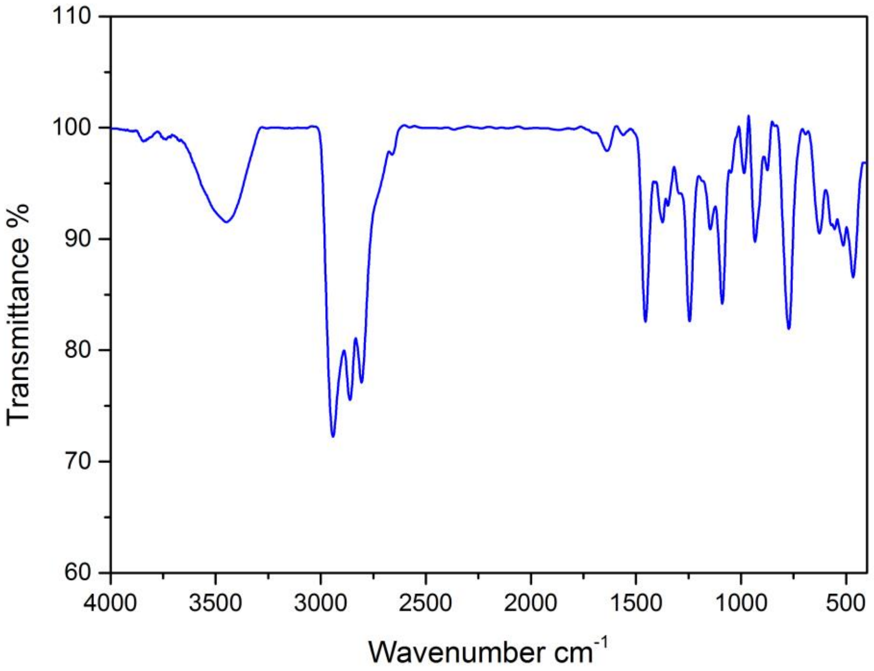

Fig. S3. FT-IR spectrum of complex **3**.

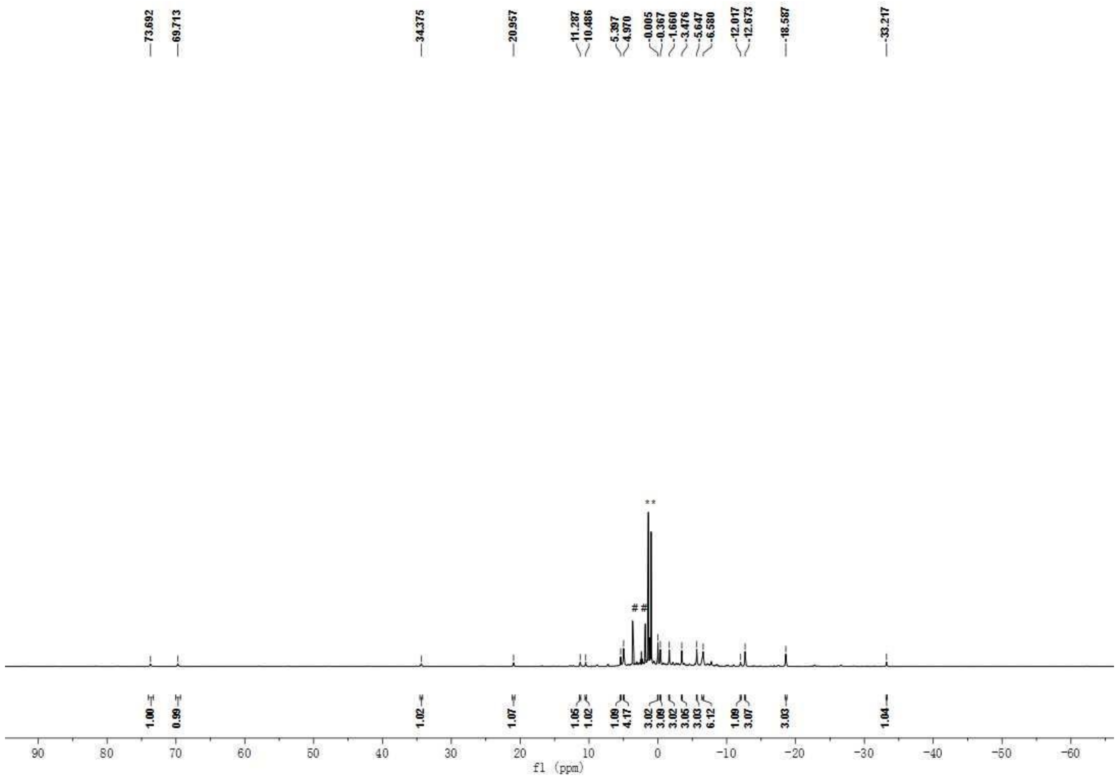

Fig. S4. The ^{1}H NMR (THF-d_8, 400 MHz) spectrum of complex **3** (# are peaks assigned to THF and * are peaks assigned to impurities in THF-d_8).

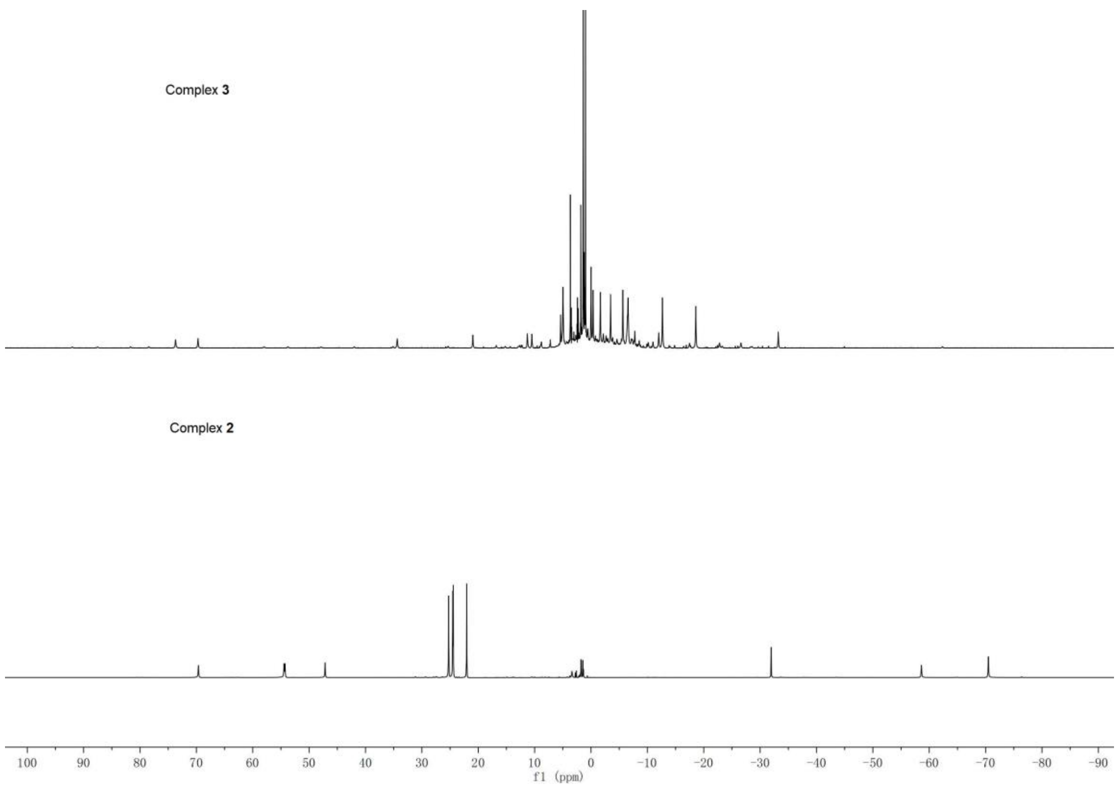

Fig. S5. The ^{1}H NMR spectra of complexes **2** and **3.**

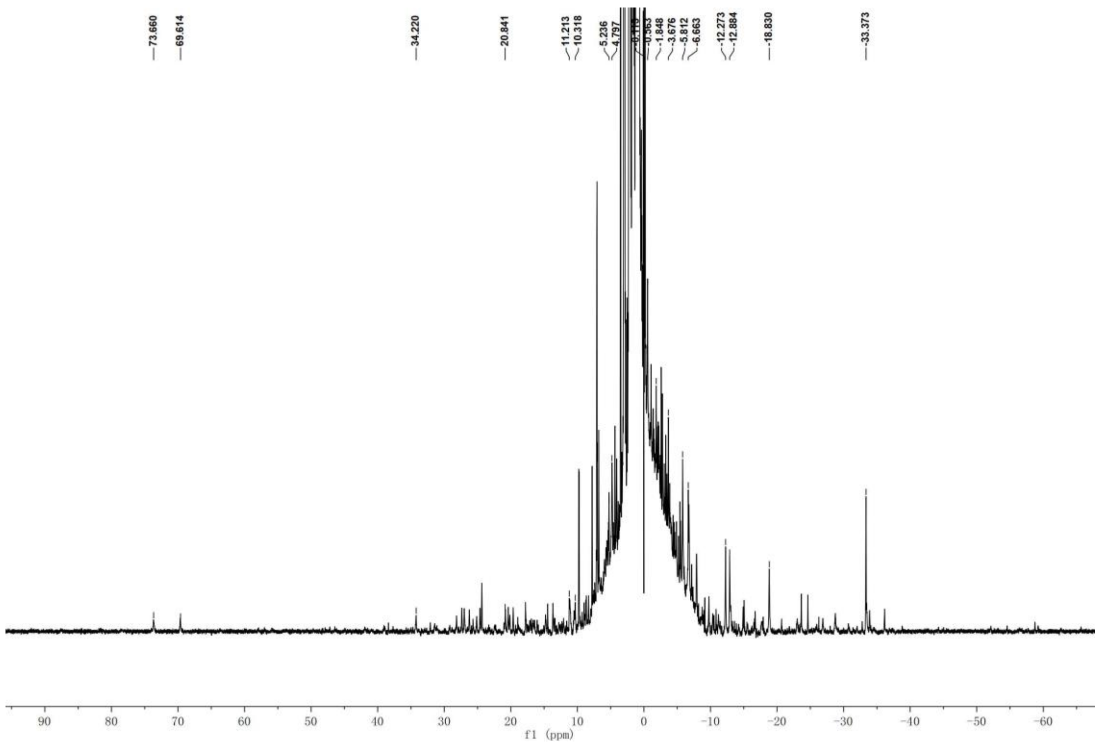

Fig. S6. The *in-situ* ^{1}H NMR (THF-d_8, 400 MHz) spectrum for the reaction of **2** with KC_8 under N_2. Complex **3** (the labelled peaks) was the sole identifiable product in this reaction.

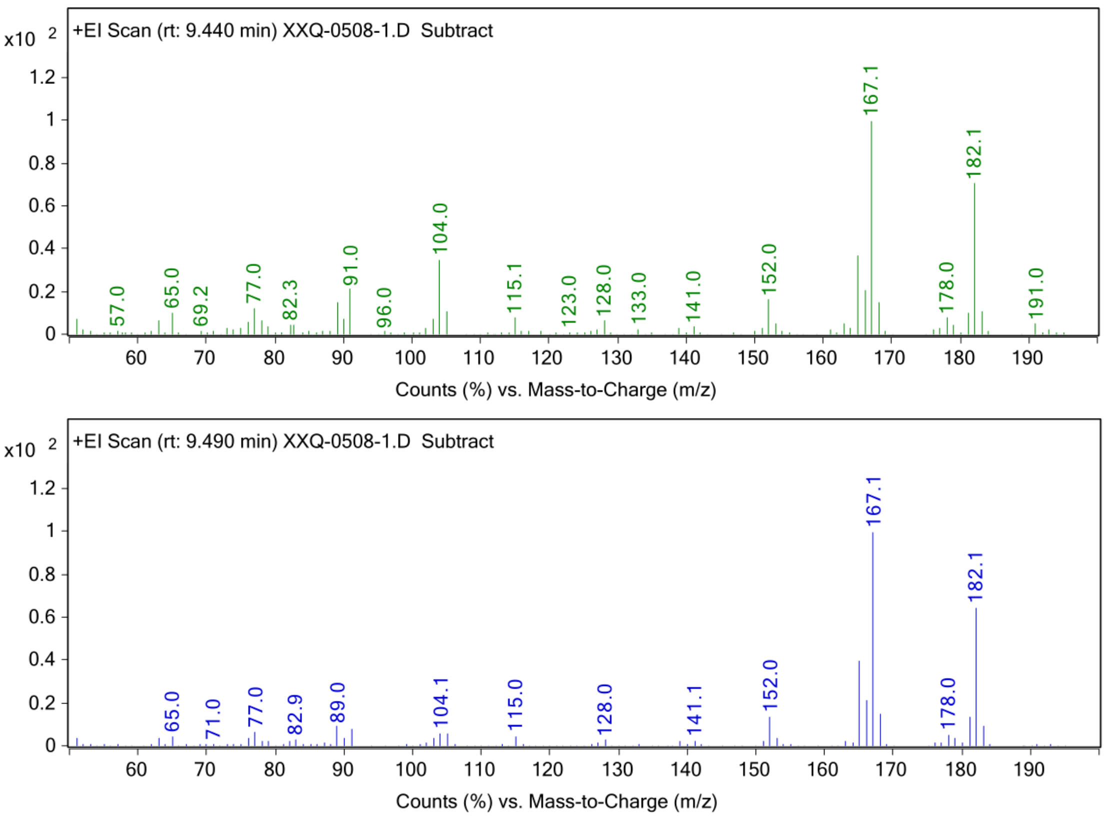

Fig. S7. GC-MS analysis of the reaction mixture of complex **2** with excess of KC_8 in toluene-THF mixed solvent under N_2 (the peak at 182.1 was assigned to dibenzyl, e.g., $PhCH_2–CH_2Ph$).

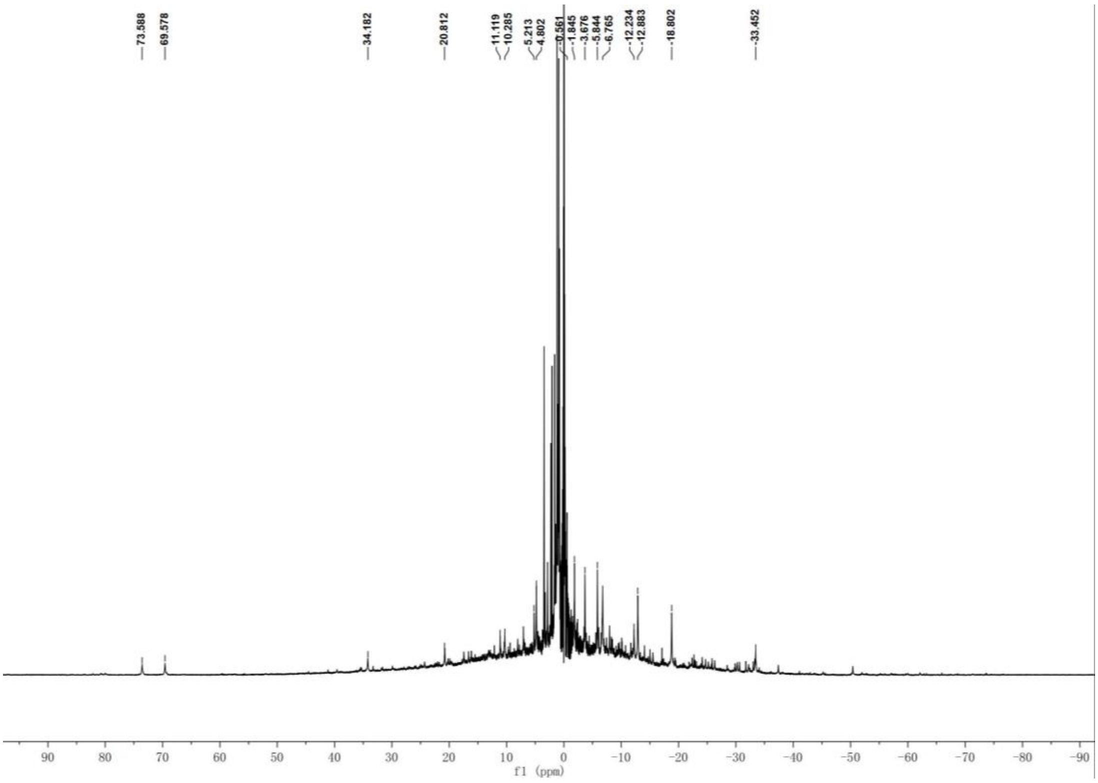

Fig. S8. The *in-situ* ^{1}H NMR (THF-d_8, 400 MHz) spectrum for the reaction of **2** with KC_8 in the presence of 9,10-dihydroanthracene under N_2 (the peaks of complex **3** were labelled).

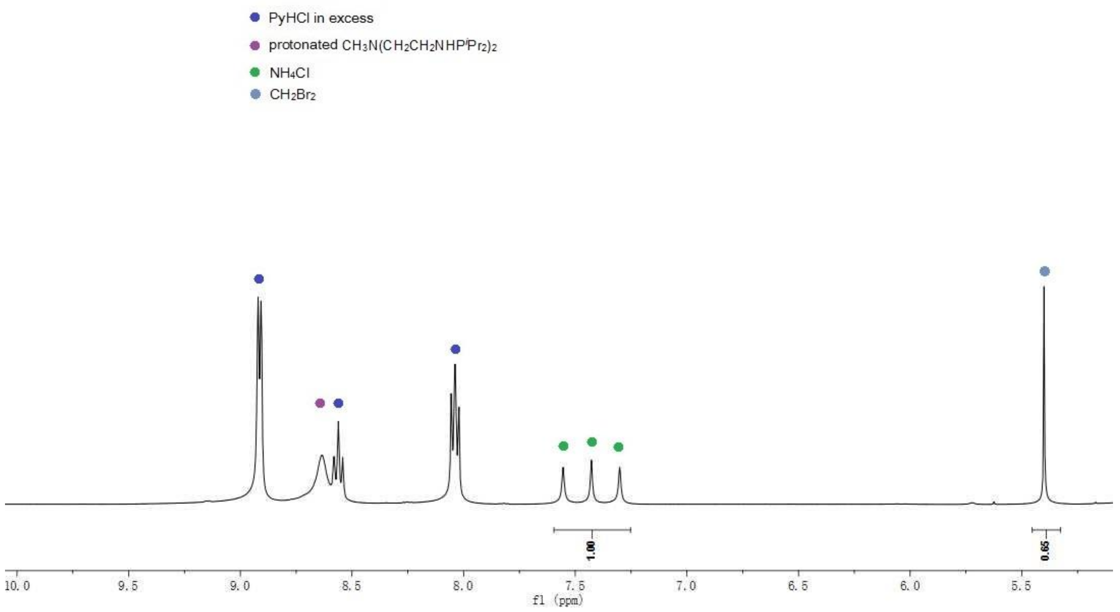

Fig. S9. The ^{1}H NMR (DMSO-d_6, 400 MHz) spectrum for the reaction of complex **3** with PyHCl (4 equiv. of dibromomethane was added for quantitative determination, 77% yield of ammonia).

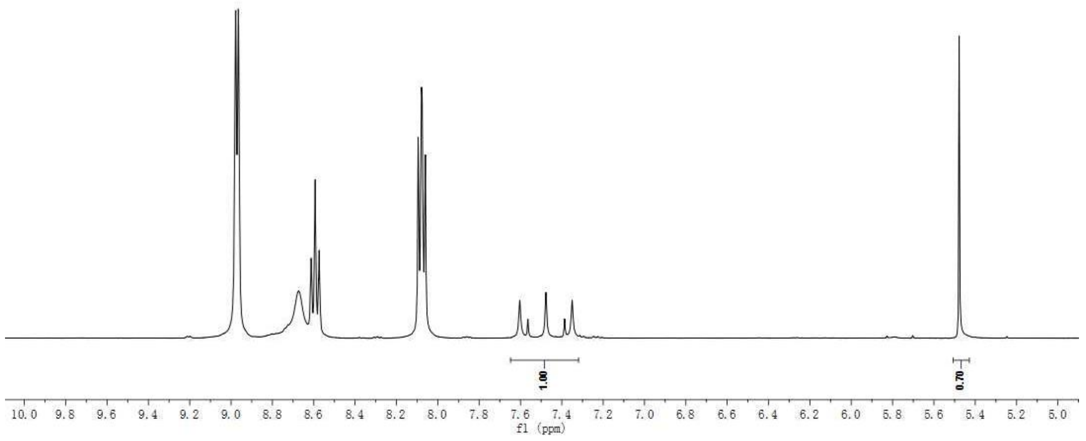

Fig. S10. The ^{1}H NMR (DMSO-d_6, 400 MHz) spectrum for the reaction of complex **3-^{15}N** with PyHCl (4 equiv. of dibromomethane was added for quantitative determination, 71% yield of ammonia).

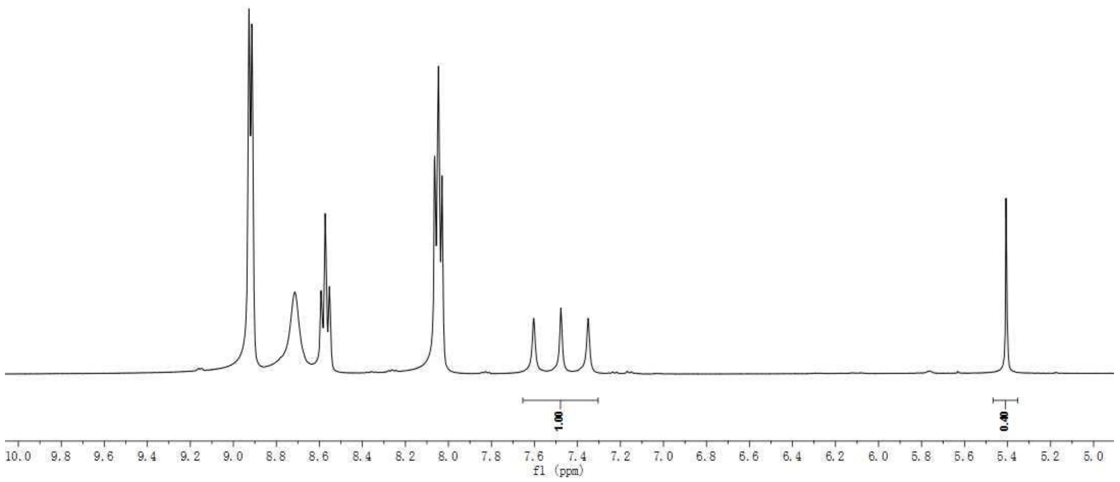

Fig. S11. The ^{1}H NMR (DMSO-d_6, 400 MHz) spectrum of NH_4Cl formed by the reaction mixture (reduction of **2** with KC_8 under N_2) with PyHCl (2 equiv. of dibromomethane was added for quantitative determination, 63% yield of ammonia).

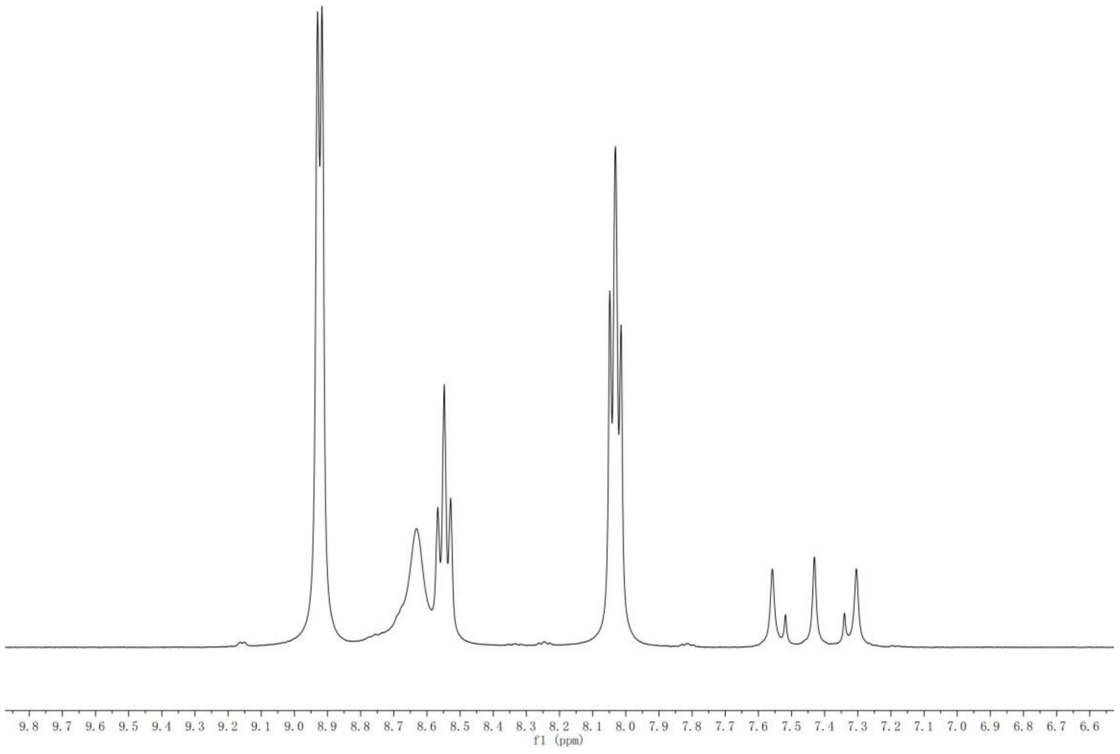

Fig. S12. The ^{1}H NMR (DMSO-d_6, 400 MHz) spectrum of NH_4Cl and $^{15}NH_4Cl$ formed by the reaction mixture (reduction of **2** with KC_8 under $^{15}N_2$) with PyHCl.

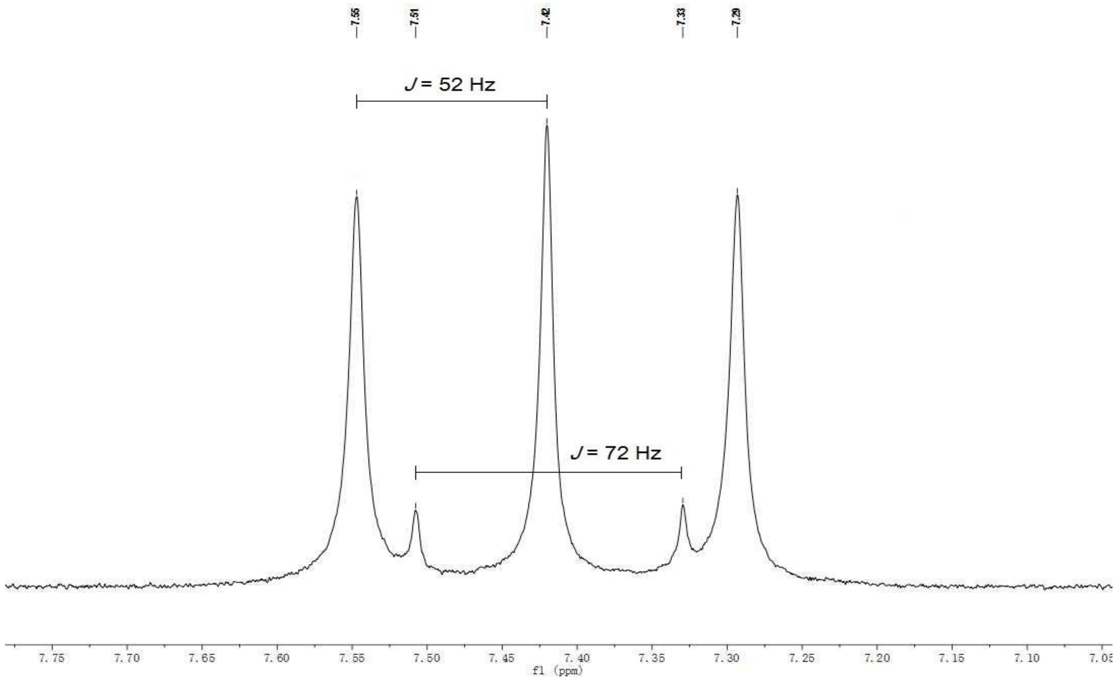

Fig. S13. The ^{1}H NMR (DMSO-d_6, 400 MHz) spectrum of NH_4Cl and $^{15}NH_4Cl$ formed by the reaction of complex **3-^{15}N** with PyHCl.

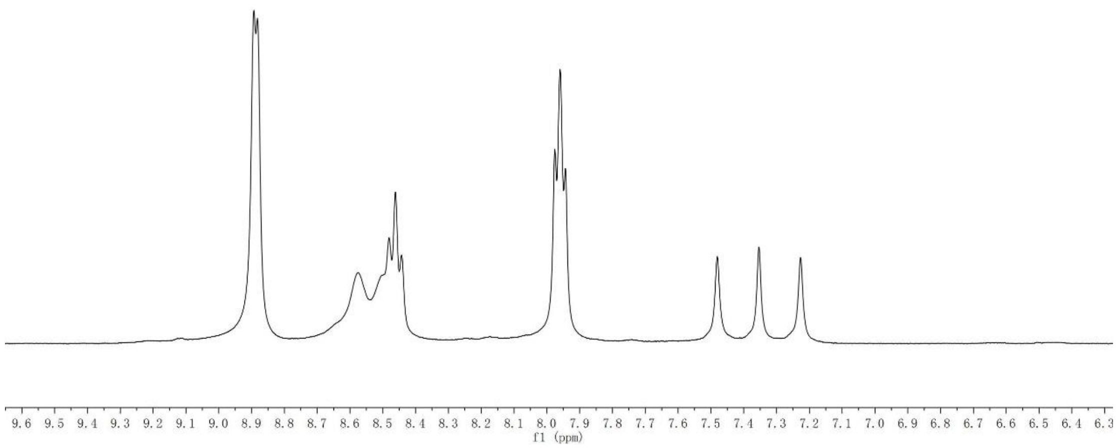

Fig. S14. The ^{1}H NMR (DMSO-d_6, 400 MHz) spectrum for the reaction of complex **3** with $^{15}N_2$ and then treated with PyHCl. Only NH_4^+ was formed in the reaction, suggesting that complex **3** can not react with $^{15}N_2$.

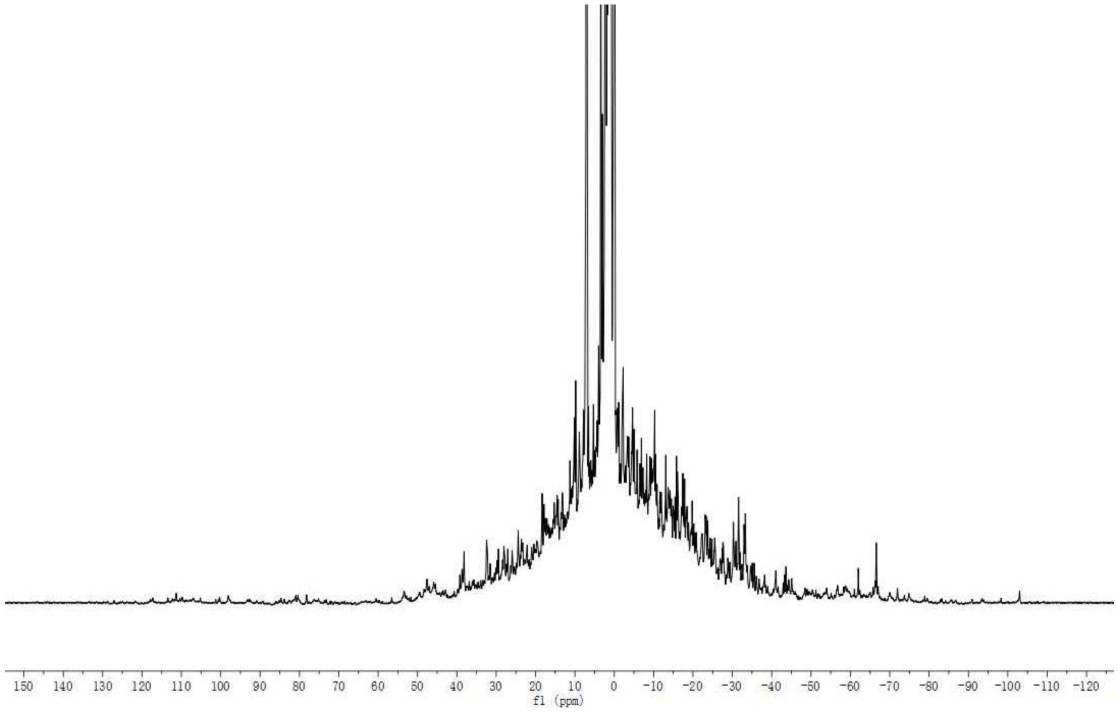

Fig. S15. The *in-situ* ^{1}H NMR (THF-d_8, 400 MHz) spectrum for the reaction of **2** with KC_8 under dynamic vacuum. No characteristic signals for complex **3** was observed. This result suggests that N_2 cleavage was necessary for the formation of complex **3**.

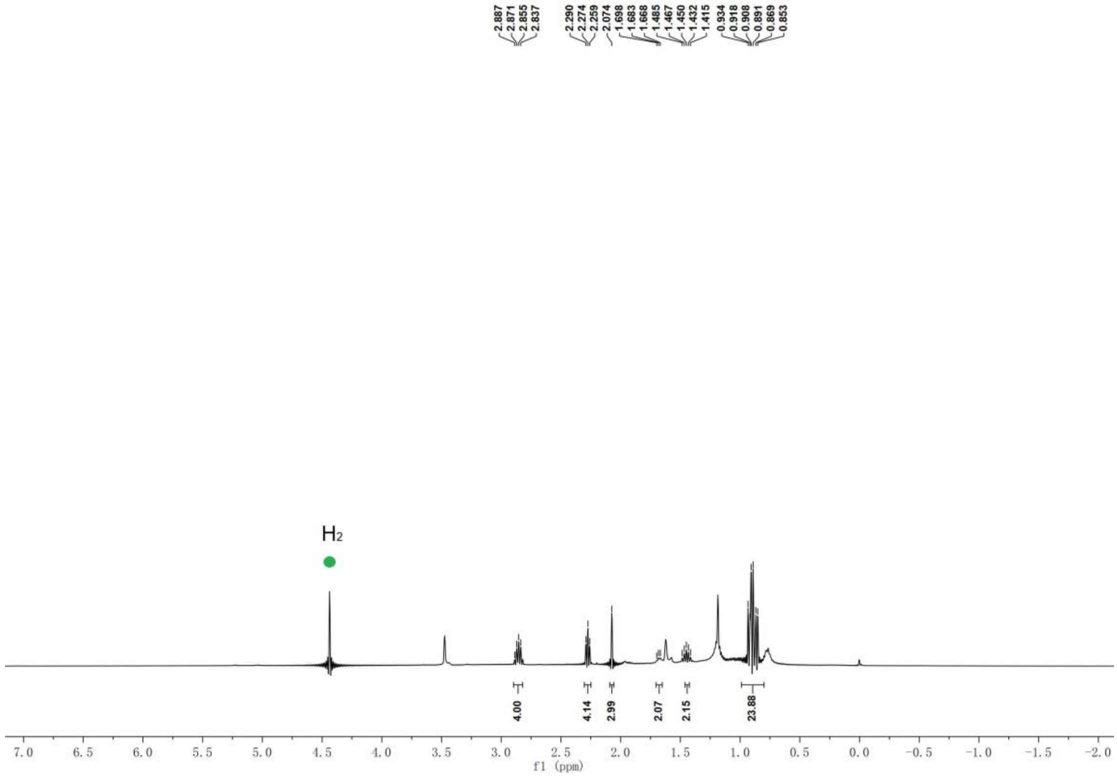

Fig. S16. The *in-situ* ^{1}H NMR (THF-d_8, 400 MHz) spectrum for the reaction of **3** with 1 atm H_2 (the labelled peaks were assigned to free ligand $CH_3N(CH_2CH_2NHP^{i}Pr_2)_2$ and the peak at 4.44 ppm corresponds to H_2).

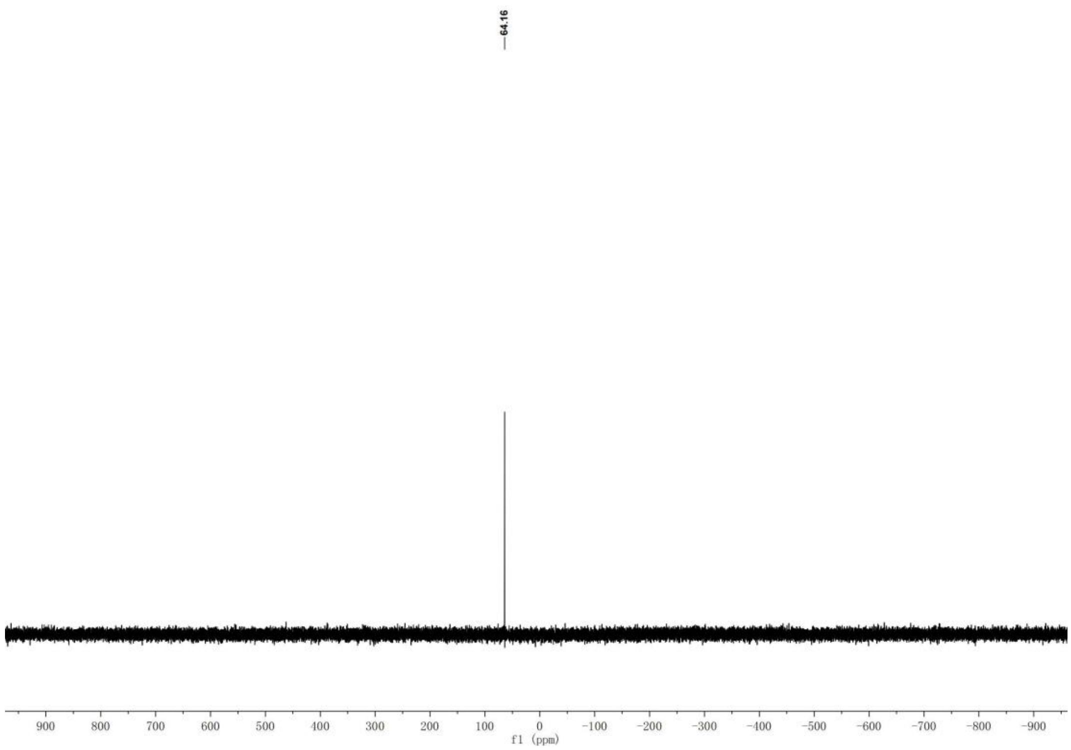

Fig. S17. The *in-situ* $^{31}P\{^1H\}$ NMR (THF-d_8, 162 MHz) spectrum for the reaction of **3** with 1 atm H_2 (the labelled peaks were assigned to free ligand $CH_3N(CH_2CH_2NHP^iPr_2)_2$).

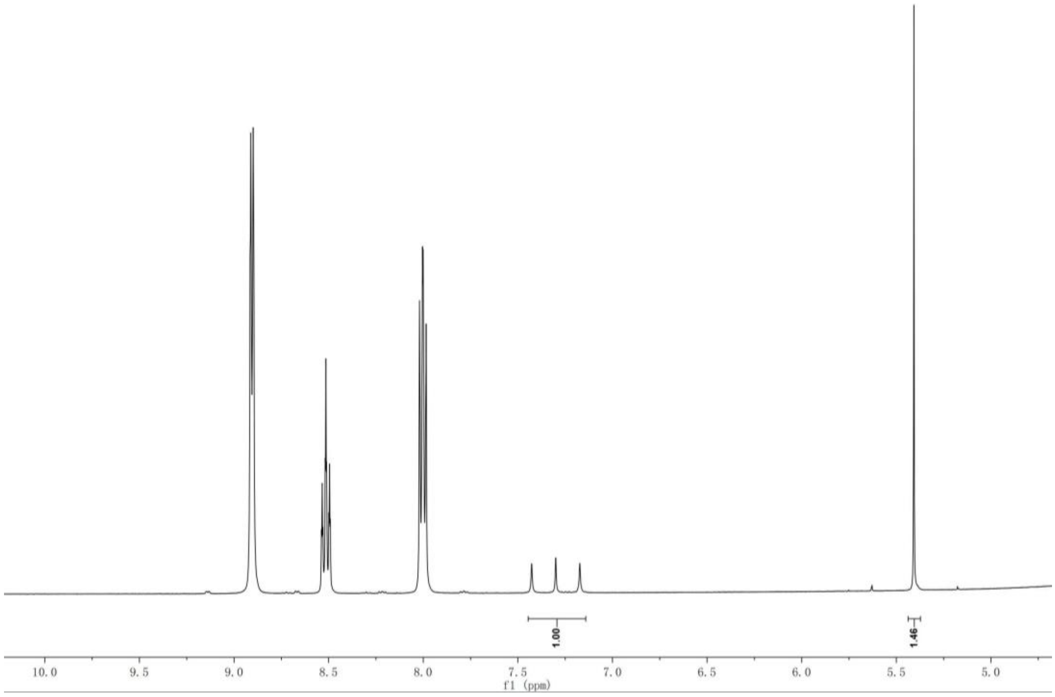

Fig. S18. The ^1H NMR (DMSO-d_6, 400 MHz) spectrum for the reaction of complex **3** with 1 atm H_2 and then treated the volatiles with excess PyHCl (4 equiv. of dibromomethane was added for quantitative determination, 34% yield of ammonia).

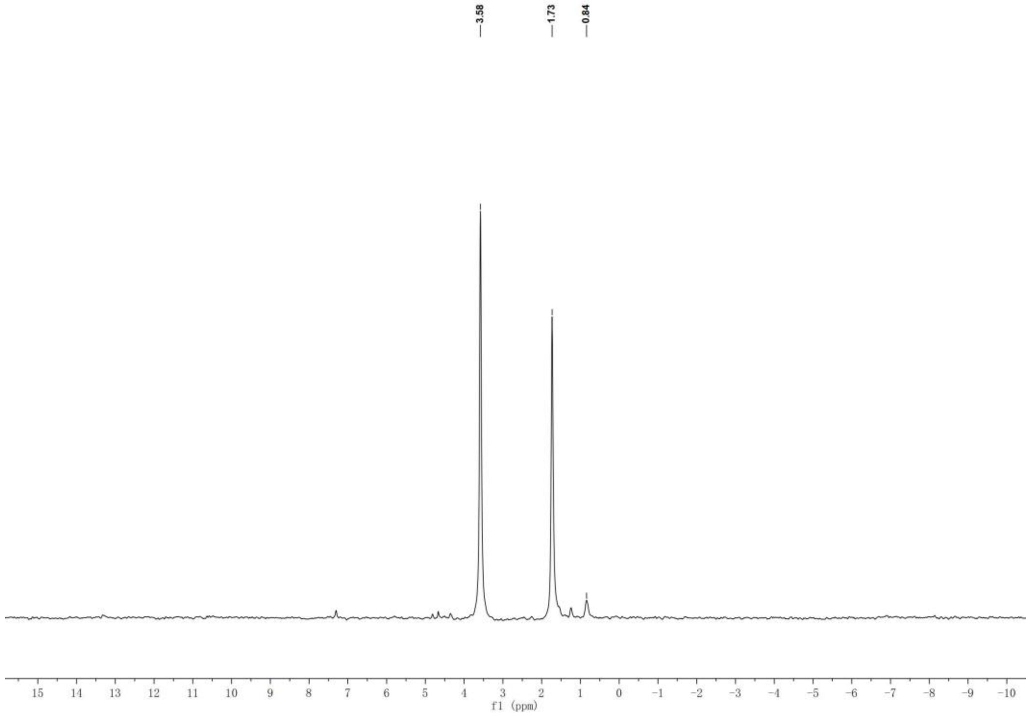

Fig. S19. The ^{2}H NMR (92 MHz) spectrum for the volatiles in THF obtained by the reaction of complex **3** with 1 atm D_2. The singlet at 0.84 ppm corresponds to ND_3. The resonances at 1.73 and 3.58 ppm correspond to the presence of natural abundance deuterated THF.

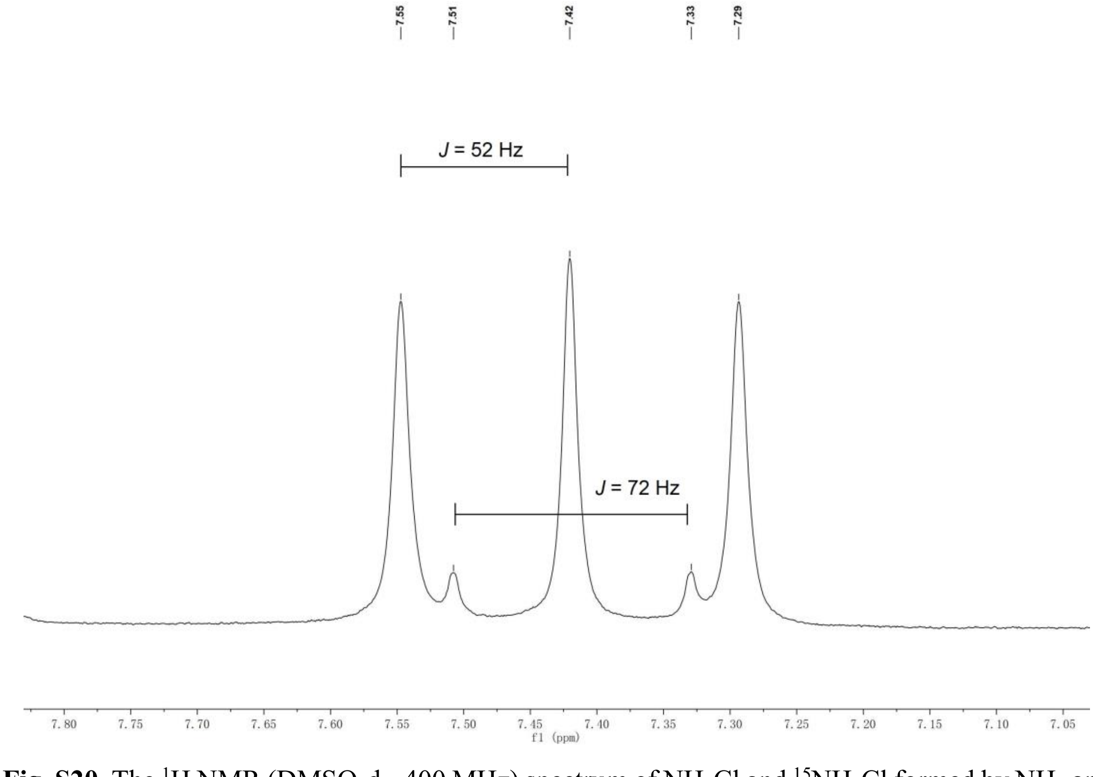

Fig. S20. The ^{1}H NMR (DMSO-d_6, 400 MHz) spectrum of NH_4Cl and $^{15}NH_4Cl$ formed by NH_3 and $^{15}NH_3$ (produced by the reaction of complex **3-^{15}N** with 1 atm H_2**)** with PyHCl. This result shows that both imido and nitride groups in complex **3** can react with H_2 to form NH_3.

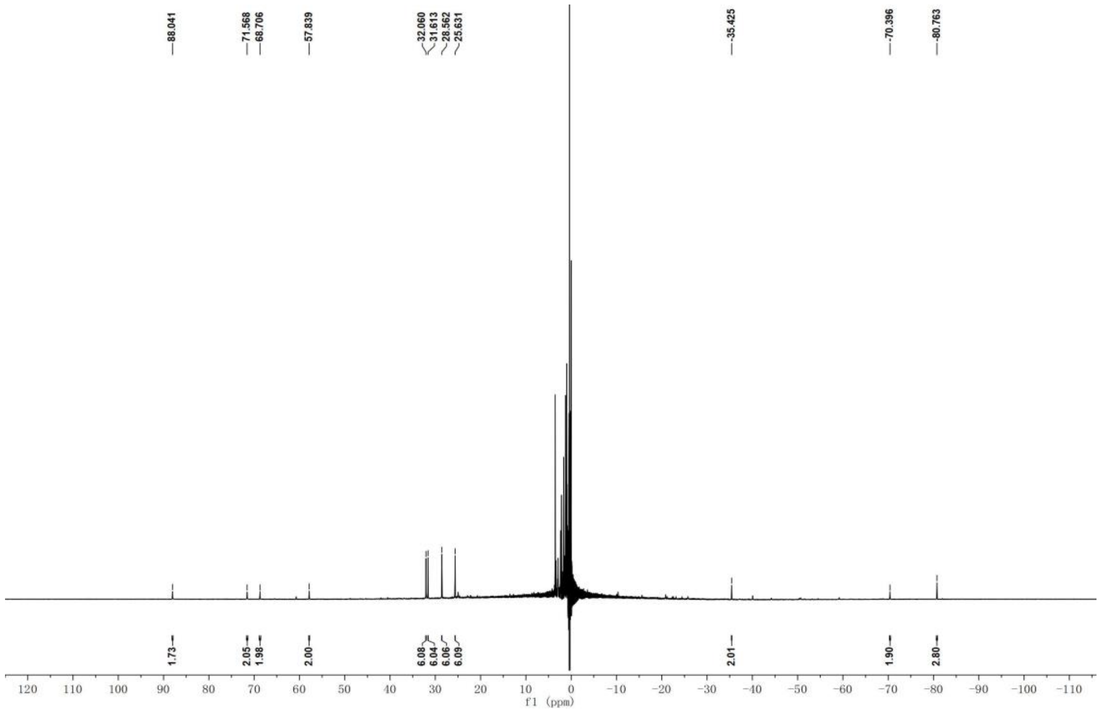

Fig. S21. The *in-situ* ^{1}H NMR (THF-d_8, 400 MHz) spectrum of the reaction of **3** with Me_3SiCl (the peaks for complex **1** are labelled). This result shows that by the reaction of complex **3** with Me_3SiCl, the uranium precursor was formed and a synthetic cycle was established.

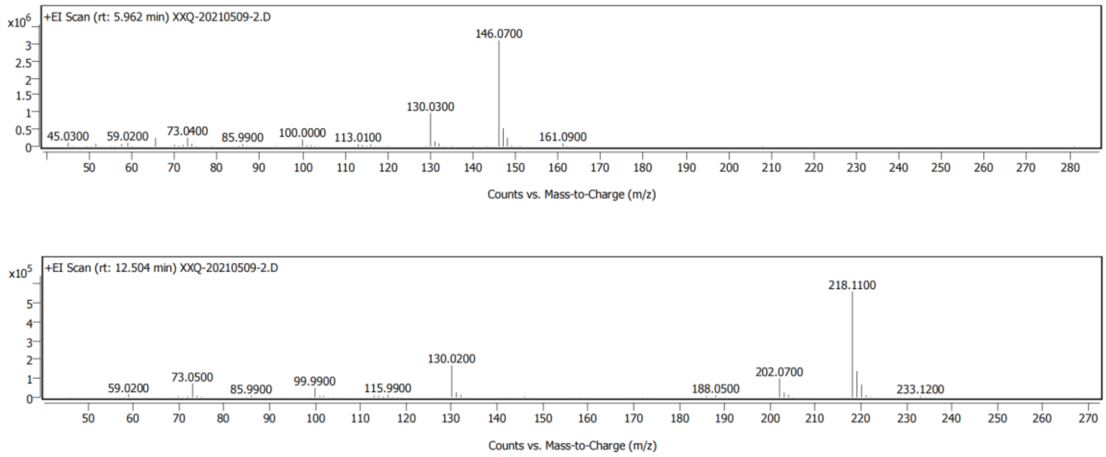

Fig. S22. GC-MS analysis of the worked-up reaction mixture of complex **3** with Me_3SiCl in THF (top: mass spectrum for $HN(SiMe_3)_2$; bottom: mass spectrum for $N(SiMe_3)_3$).

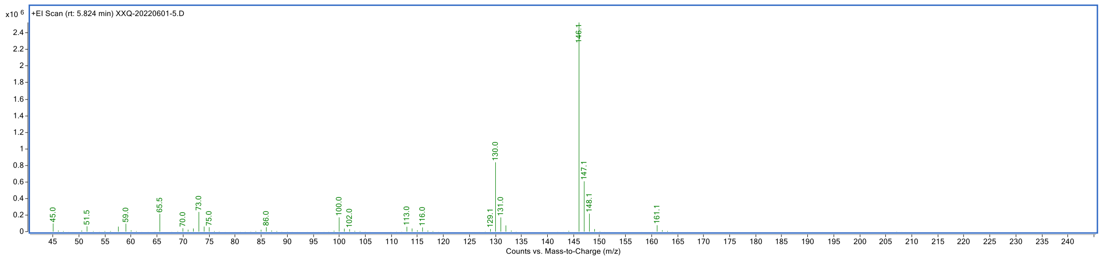

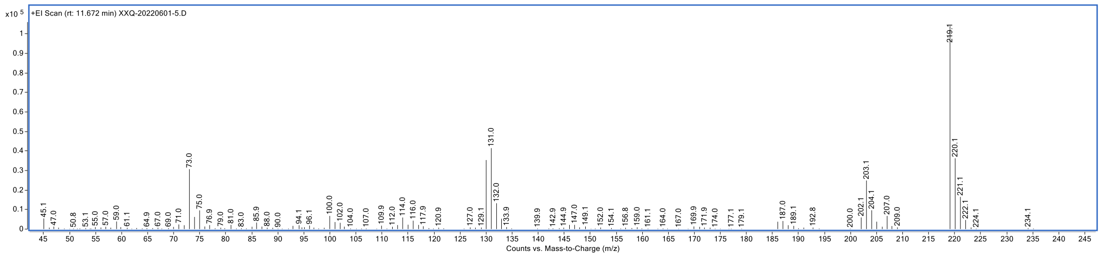

Fig. S23. GC-MS analysis of the worked-up reaction mixture of complex **3-^{15}N** with Me_3SiCl in THF (top: mass spectrum for $HN(SiMe_3)_2$; bottom: mass spectrum for $^{15}N(SiMe_3)_3$).

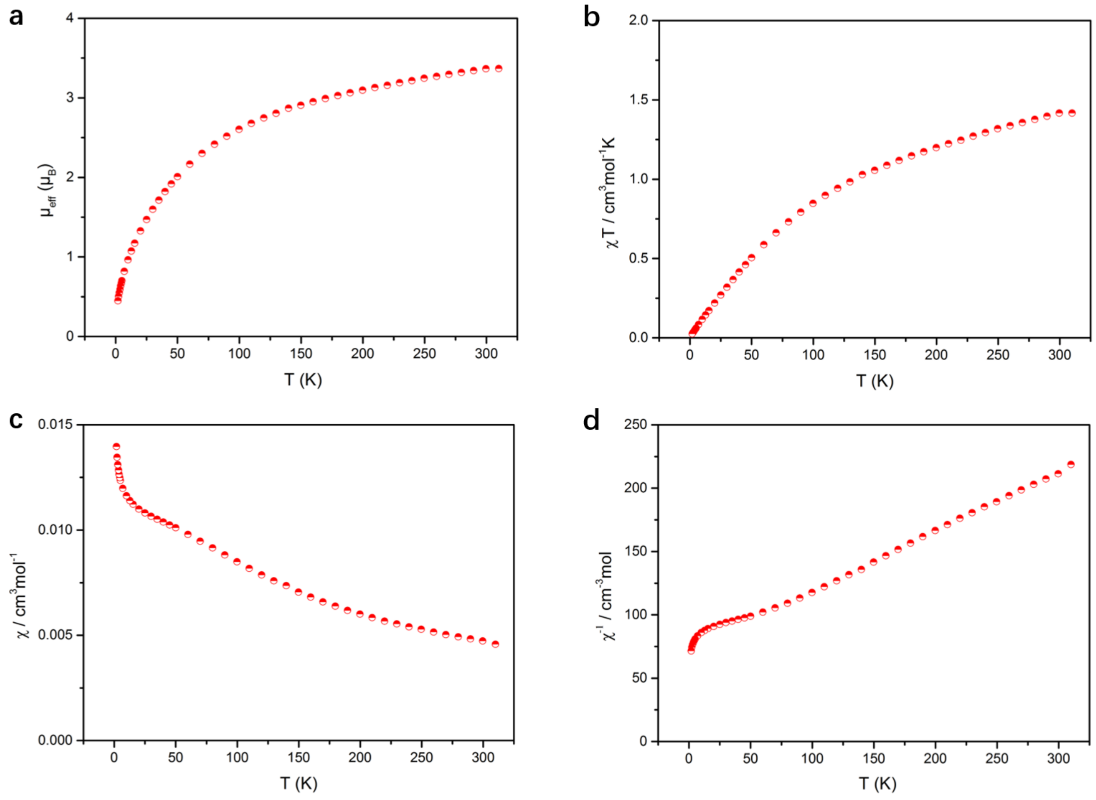

Fig. S24. Variable-temperature data of **2**. (a) μ_{eff} vs T, (b)) χT vs T, (c) χ vs T, and (d) 1/χ vs T.

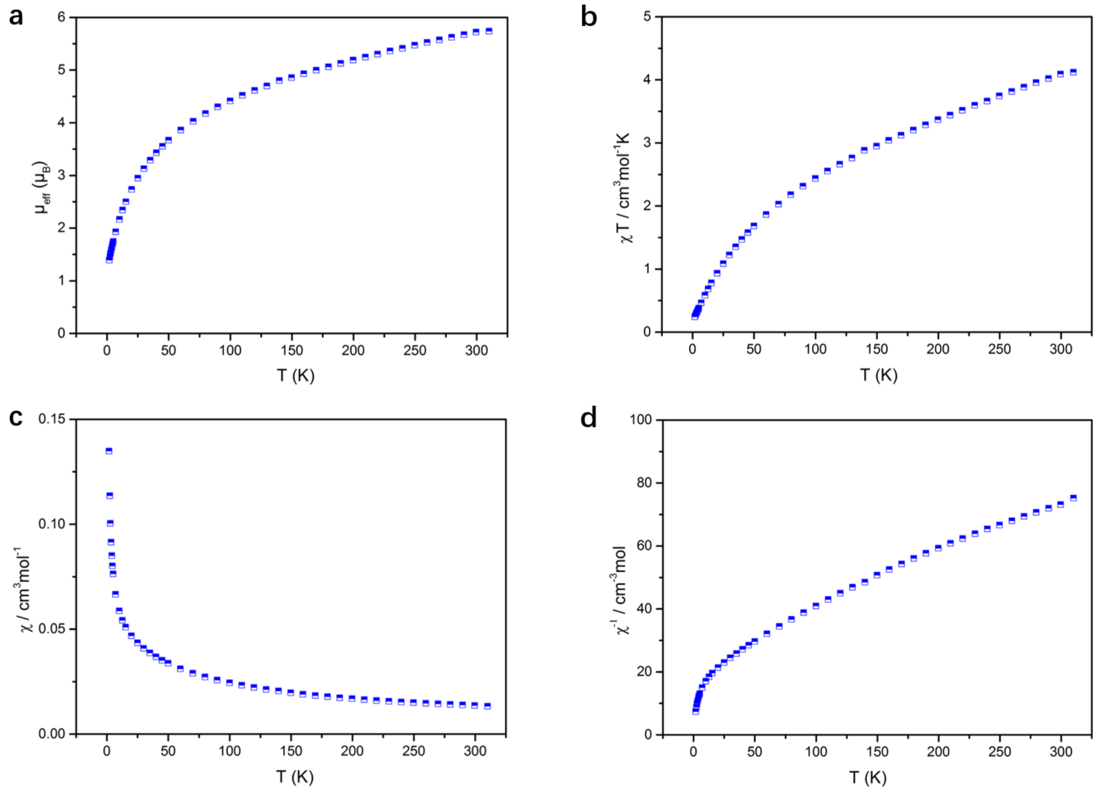

Fig. S25. Variable-temperature data of **3**. (a) μ_{eff} vs T, (b)) χT vs T, (c) χ vs T, and (d) $1/\chi$ vs T.

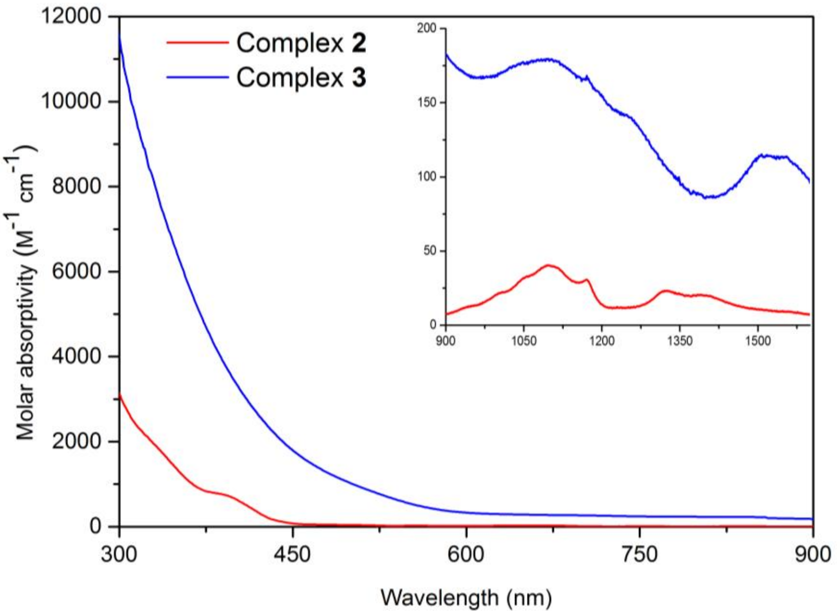

Fig. S26. UV−visible absorption spectra of complexes **2** and **3** measured in THF at RT. Inset: near-infrared absorption spectra.

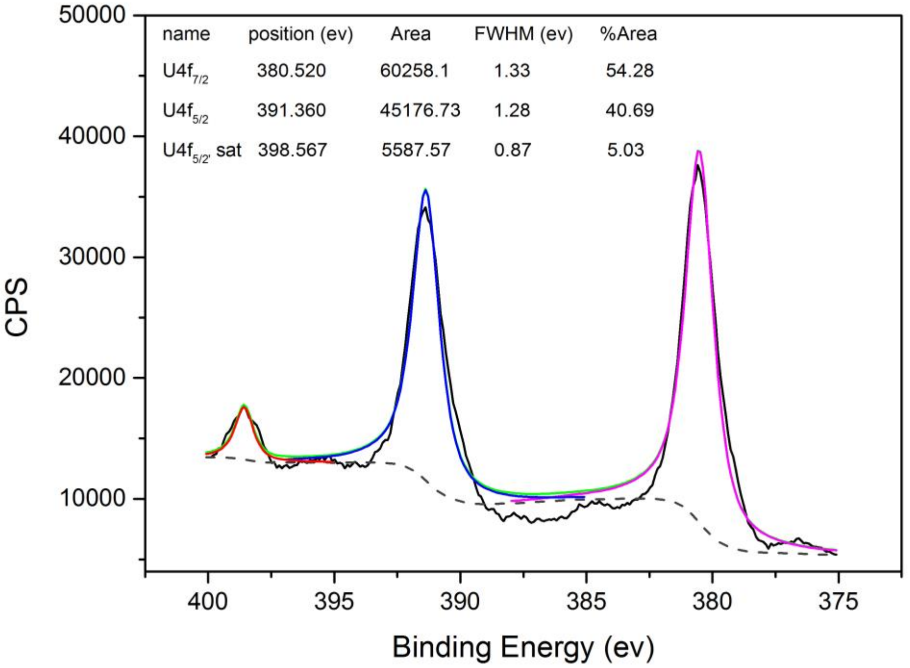

Fig. S27. XPS spectrum of U-4*f* of complex **3**.

3. X-ray crystallographic analysis

The crystallographic data were collected using a Bruker APEX-II CCD area detector with a radiation source of Ga(Kα) (1.34139 Å) or Mo(Kα) (0.71073 Å). Multi-scan or empirical absorption corrections (SADABS) were applied. The structures were solved using Patterson methods, expanded with difference Fourier syntheses, and refined using full-matrix least squares fitting on F^2 using the Bruker SHELXTL-2014 program package.[4,5] All non-hydrogen atoms were refined anisotropically. Hydrogen atoms were introduced at their geometric positions and refined as riding atoms. In complex **2**, the restraints (SIMU) were employed to displacement parameters of N4, N5 and N6. In complex **3** (prepared under Ar), pseudo-isotropic (ISOR) restraints were applied for the refinement of the disordered atoms. Two alternative orientations for the heavy atoms were refined and resulted in site occupancies of 98% and 2% for U1 and U1', U2 and U2', U3 and U3'. In complex **3** (prepared under N_2), the restraints (SADI, SIMU and RIGU) were used to refine the disordered atoms. Two alternative orientations for one carbon atom of isopropyl were refined and resulted in site occupancies of 71% and 29% for the C42 and C42'. Evaluation of the CIF using the CheckCIF routine at www.checkcif.iucr.org gave no A or B alert for these complexes. These data can be obtained free of charge from the Cambridge Crystallographic Data Centre (www.ccdc.cam.ac.uk/data-request/cif). Details regarding the data collection and refinement for these complexes are given in Table S1.

Table S1. Crystal data and structural refinements for **2** and **3**.

	**2**	**3**	
empirical formula	$C_{17}H_{39}N_9P_2U$	$C_{51}H_{120}K_2N_{13}P_6U_3$	$C_{51}H_{120}K_2N_{13}P_6U_3$
formula weight	669.54	1893.70	1893.70
temperature, K	193(2)	193(2)	190.01
wavelength, Å	0.71073	1.34139	1.34139
crystal system	Monoclinic	Monoclinic	Monoclinic
space group	$P2_1/n$	$P2_1/n$	$P2_1/n$
a, Å	15.0030(15)	14.0323(3)	14.084(3)
b, Å	7.4626(8)	21.1682(4)	21.139(4)
c, Å	23.472(2)	25.5817(5)	25.582(5)

α, °	90	90	90
β, °	96.211(4)	104.4300(10)	104.66(3)
γ, °	90	90	90
V, Å^3	2612.5(5)	7359.0(3)	7368(3)
Z	4	4	4
d_{calcd}, g cm^{-3}	1.702	1.709	1.707
μ, mm^{-1}	6.357	15.528	14.982
F(000)	1304.0	3684.0	3684.0
crystal size, mm	0.10 × 0.10 × 0.10	0.10 × 0.10 × 0.10	0.12 × 0.10 × 0.10
θ_{max},°	25.024	53.884	53.962
reflns collected	18198	63698	99102
indep reflns	4585 [R_{int} = 0.0317, R_{sigma} = 0.0279]	13433 [R_{int} = 0.0626, R_{sigma} = 0.0494]	13485 [R_{int} = 0.0605, R_{sigma} = 0.0350]
data/restraints/params	4585/18/265	13433/18/729	13485/64/714
goodness-of-fit on F^2	1.082	1.006	1.030
final *R* ($I > 2\sigma(I)$)	R_1 = 0.0215, wR_2 = 0.0527	R_1 = 0.0349, wR_2 = 0.0663	R_1 = 0.0328, wR_2 = 0.0774
R indices (all data)	R_1 = 0.0266, wR_2 = 0.0539	R_1 = 0.0554, wR_2 = 0.0743	R_1 = 0.0398, wR_2 = 0.0813
Residual electron density (e. Å^{-3}) max/min	1.91/-1.22	1.40/-1.06	1.84/-2.28
		prepared under Ar	prepared under N_2
CCDC	2021046	2021047	2044515

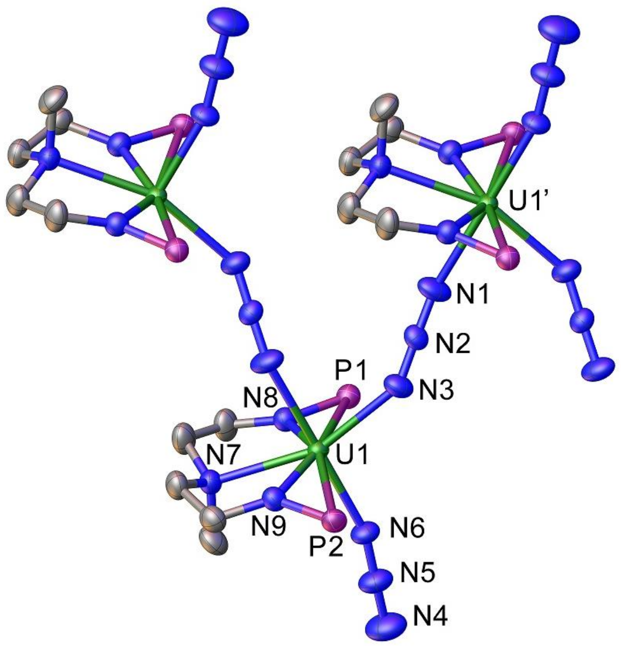

Fig. S28. X-ray molecular structure of complex **2** drawn with 50% probability. Hydrogen atoms and isopropyl moieties in $P^{i}Pr_2$ are omitted for clarity.

Table S2. Selected bond distances (Å) and angles (˚) for **2**.

N1-N2	1.159(4)	N6-U1	2.292(3)
N2-N3	1.169(4)	N7-U1	2.584(3)
N3-U1	2.447(3)	N8-U1	2.240(3)
N4-N5	1.145(5)	N9-U1	2.220(3)
N5-N6	1.181(4)	P1-U1	3.0289(10)
N8-P1	1.662(3)	P2-U1	2.9827(10)
N9-P2	1.666(3)	N1-U1’	2.471(4)
N1-N2-N3	179.0(5)	N2-N1-U1’	157.6(3)
N2-N3-U1	145.4(3)	N4-N5-N6	176.8(5)
N5-N6-U1	163.8(3)	P1-N8-U1	100.80(15)
P2-N9-U1	99.29(15)	N8-P1-U1	46.59(11)
N9-P2-U1	47.26(11)	N9-U1-N8	131.95(12)
N9-U1-N6	93.20(12)	N8-U1-N6	95.24(12)

N9-U1-N3	112.25(11)	N8-U1-N3	111.04(11)
N6-U1-N3	104.64(12)	N9-U1-N7	65.69(11)
N8-U1-N7	66.31(11)	N6-U1-N7	97.70(12)
N3-U1-N7	157.66(11)	N9-U1-P2	33.45(8)
N8-U1-P2	161.79(8)	N6-U1-P2	78.31(8)
N3-U1-P2	87.13(8)	N7-U1-P2	97.40(7)
N9-U1-P1	163.56(8)	N8-U1-P1	32.61(8)
N6-U1-P1	84.92(9)	N3-U1-P1	83.96(8)
N7-U1-P1	98.32(7)	P2-U1-P1	158.36(3)

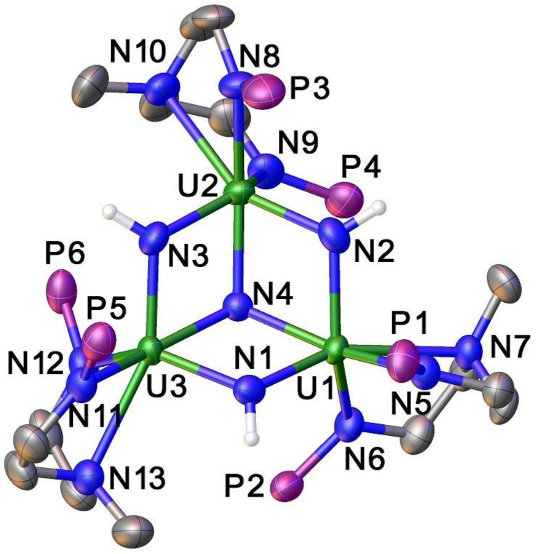

Fig. S29. X-ray molecular structure of complex **3** drawn with 50% probability. Hydrogen atoms (except for the NH protons) , isopropyl moieties in P^{i}Pr$_2$ and two K$^+$ counter ions are omitted for clarity.

Table S3. Selected bond distances (Å) and angles (°)for **3**.

U3-N3	2.188(6)	U3-N4	2.204(5)
U3-N1	2.222(5)	U3-N12	2.466(5)
U3-N11	2.380(5)	U3-N13	2.707(6)
U3-U2	3.5408(3)	U3-U1	3.5409(3)

U1-N1	2.193(5)	U1-N2	2.222(6)
U1-N4	2.218(5)	U1-N5	2.381(5)
U1-N6	2.449(5)	U1-N7	2.710(5)
U1-U2	3.5450(3)	U2-N2	2.193(5)
U2-N4	2.211(5)	U2-N3	2.231(5)
U2-N8	2.374(6)	U2-N9	2.448(6)
U2-N10	2.704(6)		
N3-U3-N4	73.87(19)	N3-U3-N1	102.49(19)
N4-U3-N1	73.27(19)	N3-U3-N11	96.70(19)
N4-U3-N11	160.62(19)	N1-U3-N11	92.89(18)
N3-U3-N12	108.3(2)	N4-U3-N12	88.78(18)
N1-U3-N12	138.22(19)	N11-U3-N12	110.42(18)
N3-U3-N13	154.63(18)	N4-U3-N13	128.82(18)
N1-U3-N13	96.00(18)	N11-U3-N13	64.86(18)
N12-U3-N13	66.15(18)	N3-U3-U2	37.17(14)
N4-U3-U2	36.75(13)	N1-U3-U2	88.66(13)
N11-U3-U2	132.29(14)	N12-U3-U2	99.12(13)
N13-U3-U2	162.14(13)	N3-U3-U1	89.03(14)
N4-U3-U1	36.92(13)	N1-U3-U1	36.39(14)
N11-U3-U1	128.36(13)	N12-U3-U1	116.17(13)
N13-U3-U1	115.91(12)	U2-U3-U1	60.077(7)
N1-U1-N4	73.56(18)	N1-U1-N2	102.6(2)
N4-U1-N2	73.03(19)	N1-U1-N5	95.43(18)
N4-U1-N5	159.08(19)	N2-U1-N5	92.76(19)
N1-U1-N6	108.59(18)	N4-U1-N6	89.29(18)
N2-U1-N6	137.86(19)	N5-U1-N6	111.28(18)
N1-U1-N7	153.45(17)	N4-U1-N7	130.58(17)
N2-U1-N7	96.24(18)	N5-U1-N7	64.89(17)

N6-U1-N7	66.48(17)	N1-U1-U3	36.95(13)
N4-U1-U3	36.65(13)	N2-U1-U3	88.44(14)
N5-U1-U3	130.58(12)	N6-U1-U3	99.80(12)
N7-U1-U3	163.77(11)	N1-U1-U2	89.00(13)
N4-U1-U2	36.76(13)	N2-U1-U2	36.31(14)
N5-U1-U2	127.89(13)	N6-U1-U2	116.21(12)
N7-U1-U2	116.94(11)	U3-U1-U2	59.959(7)
N2-U2-N4	73.7(2)	N2-U2-N3	102.2(2)
N4-U2-N3	72.88(19)	N2-U2-N8	96.3(2)
N4-U2-N8	159.66(19)	N3-U2-N8	92.6(2)
N2-U2-N9	107.5(2)	N4-U2-N9	89.32(19)
N3-U2-N9	139.3(2)	N8-U2-N9	110.8(2)
N2-U2-N10	154.0(2)	N4-U2-N10	129.2(2)
N3-U2-N10	97.4(2)	N8-U2-N10	65.6(2)
N9-U2-N10	65.7(2)	N2-U2-U3	88.90(14)
N4-U2-U3	36.60(13)	N3-U2-U3	36.33(14)
N8-U2-U3	127.96(14)	N9-U2-U3	116.78(14)
N10-U2-U3	116.87(14)	N2-U2-U1	36.87(15)
N4-U2-U1	36.89(13)	N3-U2-U1	88.24(14)
N8-U2-U1	131.41(14)	N9-U2-U1	99.10(14)
N10-U2-U1	162.06(16)	U3-U2-U1	59.964(6)

4. Theoretical Calculations

All calculations were carried out at the DFT level of theory using the hybrid functional B3PW91[6-7] with the Gaussian 09 suite of programs.[8] The U and P atoms were represented with a small-core Stuttgart-Dresden relativistic effective core potential associated with their adapted basis set.[9-11] Additionally, the P basis set was augmented by a d-polarization function ($\alpha = 0.340$)[12] to represent the valence orbitals. All the other atoms C, N, K and H were described with a 6-31G (d,p) double–ζ quality basis set.[13-14] The nature of the extrema (minimum) was established with analytical frequencies calculations and geometry optimizations were computed without any symmetry constraints. The enthalpy energy was computed at T = 298 k in the gas phase. Dispersion corrections were included by means of single point energy calculations using the GD3BJ approach.[15] The redox potential K/K^+ was estimated from our calculation and compared to the experimental value using the methodology published by Castro *et al.*[16] The computed redox potential is -3.01 V vs. -2.99 V experimentally which supports the correctness of our modelling of the electron transfer properties of KC_8. Spin-orbit corrections were not considered in this study since the molecular environment will mainly quenched the spin-orbit effect due to the ligand field and the strong hybridization of the uranium atomic orbitals.. The differential SO correction was found to be 1.3 kcal/mol in the redox step suing the CIPSO methods.[17,18]

Two facts suggest the significance of the potassium. First, the release of N_2 from the monomeric form of **2'** in the absence of K was calculated to imply an activation barrier of 46.7 kcal mol^{-1} (28.6 kcal mol^{-1} from the uncapped monoazide complex). The N-N bond cleavage implies a single electron transfer from the uranium center to the azide ligand. Indeed, at the **TS1**, the unpaired spin density appears to be distributed between U (2.19), in line with a U(IV) system, and the two terminal nitrogen atoms of the azide ligand (0.54 for the nitride and 0.42 for the N_2), that are stabilized by the potassium cation. At the **TS2**, the assistance from the potassium is again crucial since an interaction between one potassium and the phenyl ring of the toluene is observed, allowing the proton transfer.

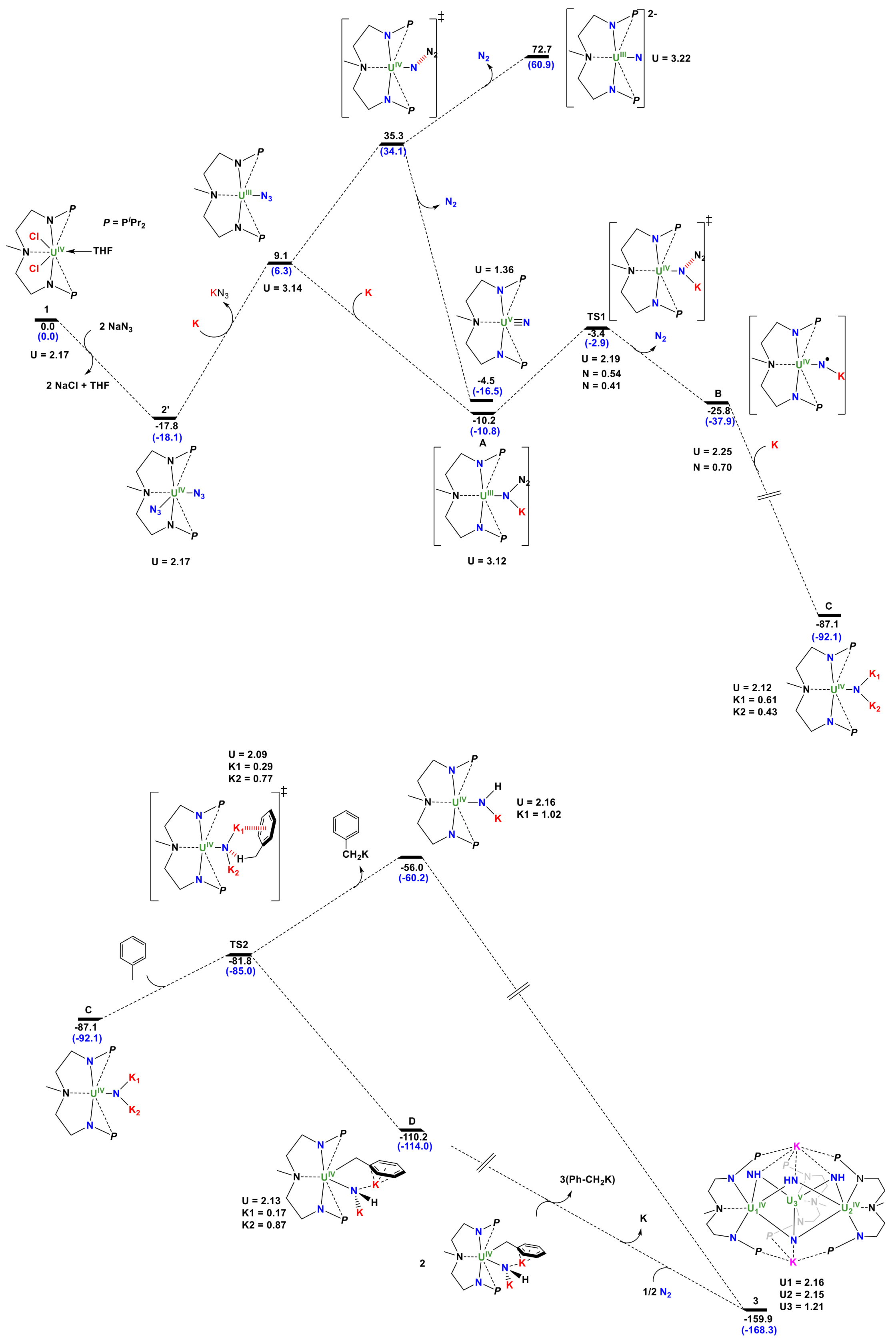

Fig. S30. Computed enthalpy profile at RT with all possible reactions including dispersion corrections (GD3BJ). The values in brackets are the Gibbs free energy.

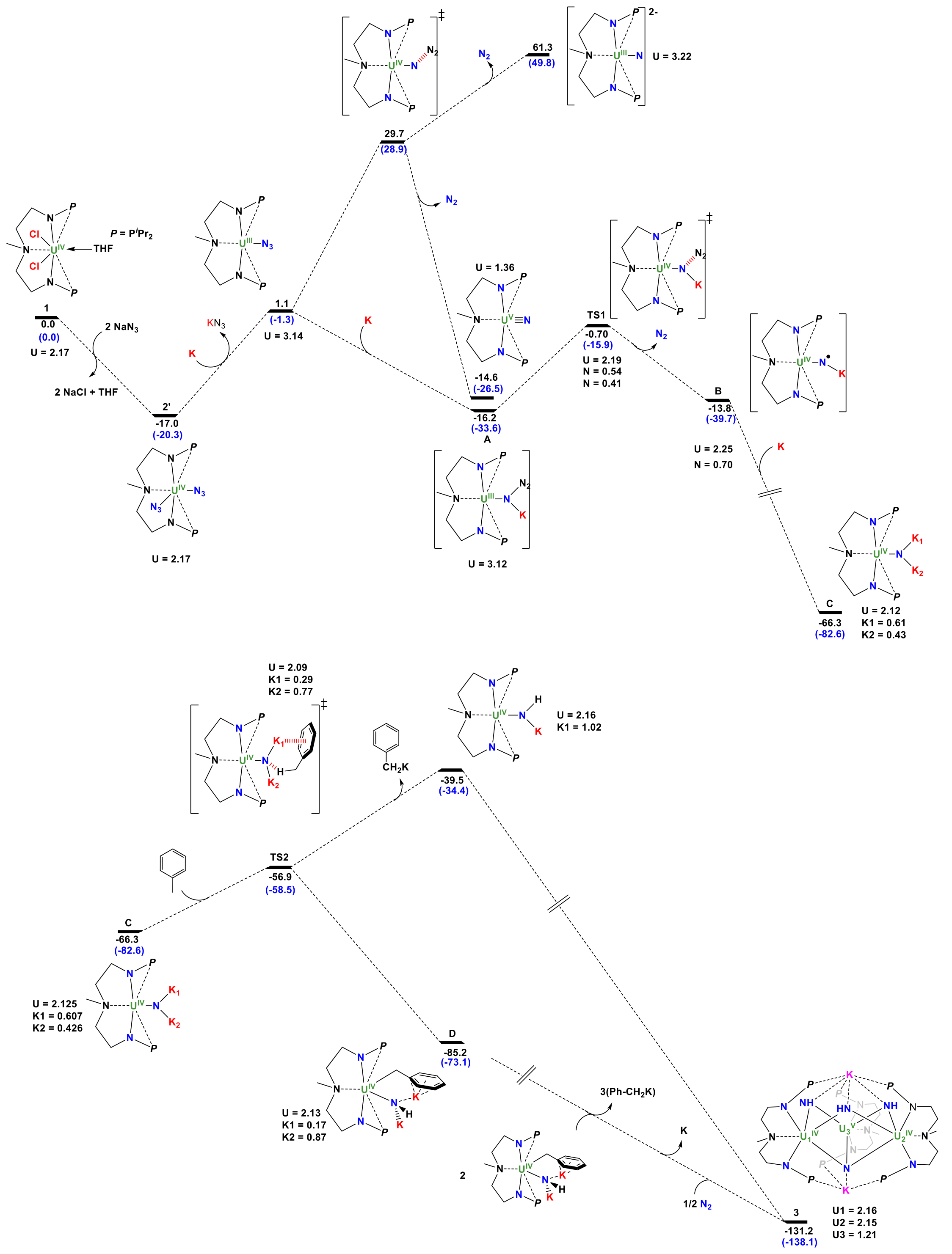

Fig. S31. Computed enthalpy profile at RT including all possible reactions. The values in brackets are the Gibbs free energy.

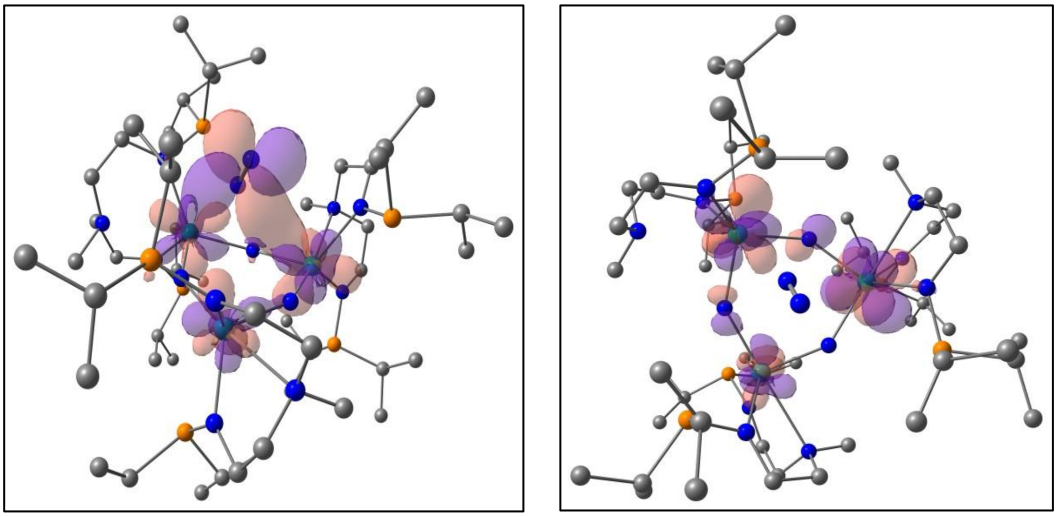

Fig. S32. The molecular orbitals of **F** (left: LUMO; right: HOMO).

Table S4. Charges and spin multiplicity of complex **1** to **3**.

Complex	Charge	Spin Multiplicity
**1**	U = 1.378757	3
**2'**	U = 1.648714	3
**1.1**	U = 1.221655	4
**A**	U = 0.959109	4
**B**	U = 1.137101<br>N = -0.667924	4
**C**	U = 0.937710<br>K = 0.223070<br>K = 0.335016	4
**D**	U = 1.330402<br>K = -0.002073<br>K = 0.498437	4
**E**	U1 = 1.500017<br>U2 = 1.540897<br>U3 = 1.546787	7
**F**	U1 = 1.532365<br>U2 = 1.534958<br>U3 = 1.535494	7
**G**	U1 = 1.502763<br>U2 = 1.516836<br>U3 = 1.656473	6
**3**	U1 = 1.533588<br>U2 = 1.491044<br>U3 = 1.723086	6

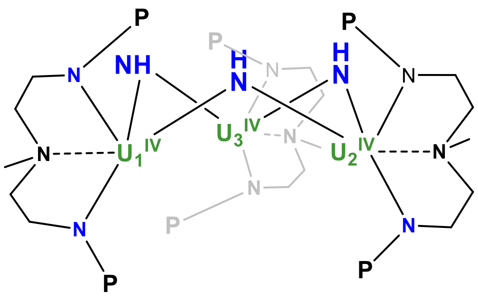

U1=2.179
U2=2.168
U3=2.181

BD (1) U 20 - N 33
(13.48%) 0.3671* U 20 s(6.68%)p 0.64(4.26%)d 6.61(44.11%)f 6.73(44.93%)
(86.52%) 0.9302* N 33 s(38.88%)p 1.57(61.11%)d 0.00(0.01%)
BD (1) N 33 - U 34
(86.62%) 0.9307* N 33 s(41.25%)p 1.42(58.74%)d 0.00(0.01%)
(13.38%) 0.3658* U 34 s(11.45%)p 0.26(2.97%)d 4.61(52.73%)f 2.87(32.81%)
BD (1) N 33 - H 103
(70.78%) 0.8413* N 33 s(19.39%)p 4.15(80.56%)d 0.00(0.05%)
(29.22%) 0.5406* H 103 s(99.90%)p 0.00(0.10%)

BD (1) U 20 - N 21
(13.73%) 0.3706* U 20 s(10.31%)p 0.38(3.96%)d 5.13(52.93%)
(86.27%) 0.9288* N 21 s(41.19%)p 1.43(58.81%)d 0.00(0.01%)
BD (1) N 21 - U 22
(87.04%) 0.9330* N 21 s(39.01%)p 1.56(60.98%)d 0.00(0.01%)
(12.96%) 0.3600* U 22 s(9.09%)p 0.63(5.75%)d 5.01(45.58%)f 4.35(39.55%)
BD (1) N 21 - H 104
(70.38%) 0.8389* N 21 s(19.72%)p 4.07(80.22%)d 0.00(0.05%)
(29.62%) 0.5442* H 104 s(99.90%)p 0.00(0.10%)

BD (1) U 22 - N 35
(12.93%) 0.3596* U 22 s(9.63%)p 0.71(6.80%)d 5.67(54.55%)f 3.01(28.98%)
(87.07%) 0.9331* N 35 s(43.00%)p 1.33(56.99%)d 0.00(0.01%)
BD (1) U 34 - N 35
(13.36%) 0.3655* U 34 s(6.80%)p 0.43(2.91%)d 6.10(41.48%)f 7.17(48.79%)
(86.64%) 0.9308* N 35 s(35.99%)p 1.78(63.99%)d 0.00(0.01%)

BD (1) N 35 - H 105
(70.31%) 0.8385* N 35 s(20.55%)p 3.86(79.39%)d 0.00(0.05%)
(29.69%) 0.5449* H 105 s(99.90%)p 0.00(0.10%)

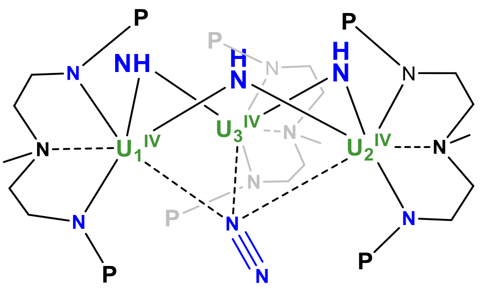

U1=2.182
U2=2.166
U3=2.160

<u>Wiberg Bond Index</u>
N19 - U3 = 0.26

<u>Second Order Perturbation Theory</u>
From the **σ bond N19 – N194 to the U1 = 2.0 kcal/mol**
From the **σ bond N19 – N194 to the U2 = 2.0 kcal/mol**
From the **σ bond N19 – N194 to the U3 = 8.0 kcal/mol**

From the **π bond N19 – N194 to the U1 = 2.0 kcal/mol**
From the **π bond N19 – N194 to the U1 = 1.8 kcal/mol**
From the **π bond N19 – N194 to the U1 = 1.1 kcal/mol**

From the second **π bond N19 – N194 to the U1 = 1.0 kcal/mol**
From the second **π bond N19 – N194 to the U1 = 1.0 kcal/mol**
From the second **π bond N19 – N194 to the U1 = 6.4 kcal/mol**

From LP of N19 **to the U1 = 10.0 kcal/mol**
From LP of N19 **to the U2 = 7.0 kcal/mol**
From LP of N19 **to the U3 = 53.0 kcal/mol**

<u>Bond</u>
BD (1) U 1 - N 16
(13.01%) 0.3607* U 1 s(5.17%)p 0.44(2.30%)d 8.05(41.63%)f 9.84(50.87%)g 0.01(0.03%)
(86.99%) 0.9327* N 16 s(36.05%)p 1.77(63.94%)d 0.00(0.01%)
BD (1) U 2 - N 16
(12.31%) 0.3509* U 2 s(8.82%)p 0.37(3.28%)d 6.66(58.74%)f 3.30(29.10%)g 0.01(0.06%)
(87.69%) 0.9364* N 16 s(43.02%)p 1.32(56.97%)d 0.00(0.01%)
BD (1) N 16 - H 161

(70.17%) 0.8377* N 16 s(20.59%)p 3.85(79.35%)d 0.00(0.05%)
(29.83%) 0.5461* H 161 s(99.90%)p 0.00(0.10%)

BD (1) U 2 - N 17
(12.61%) 0.3551* U 2 s(4.56%)p
0.56(2.54%)d10.34(47.17%)f10.01(45.69%)g 0.01(0.03%)
(87.39%) 0.9348* N 17 s(35.65%)p 1.80(64.34%)d 0.00(0.01%)
BD (1) U 3 - N 17
(12.95%) 0.3598* U 3 s(8.70%)p 0.79(6.90%)d 6.87(59.79%)f
2.82(24.55%)g 0.01(0.05%)
(87.05%) 0.9330* N 17 s(44.33%)p 1.26(55.66%)d 0.00(0.01%)
BD (1) N 17 - H 162
(69.80%) 0.8355* N 17 s(19.94%)p 4.01(80.01%)d 0.00(0.05%)
(30.20%) 0.5495* H 162 s(99.91%)p 0.00(0.09%)

BD (1) U 1 - N 18
(13.07%) 0.3615* U 1 s(9.48%)p 0.26(2.45%)d 5.60(53.07%) f
3.68(34.94%)
(86.93%) 0.9324* N 18 s(39.91%)p 1.51(60.09%)d 0.00(0.01%)
BD (1) U 3 - N 18
(13.83%) 0.3719* U 3 s(5.56%)p 0.85(4.73%)d 8.78(48.85%)f
7.34(40.84%)
(86.17%) 0.9283* N 18 s(41.01%)p 1.44(58.98%)d 0.00(0.01%)
BD (1) N 18 - H 163
(70.65%) 0.8405* N 18 s(18.74%)p 4.33(81.21%)d 0.00(0.05%)
(29.35%) 0.5418* H 163 s(99.90%)p 0.00(0.10%)

BD (1) N 19 - N 194
(51.09%) 0.7148* N 19 s(37.46%)p 1.66(62.30%)d 0.01(0.24%)
(48.91%) 0.6994* N 194 s(36.04%)p 1.77(63.62%)d 0.01(0.33%)
BD (2) N 19 - N 194
(50.40%) 0.7099* N 19 s(0.03%)p99.99(99.56%)d13.79(0.41%)
(49.60%) 0.7043* N 194 s(0.01%)p99.99(99.54%)d30.03(0.45%)
BD (3) N 19 - N 194
(50.68%) 0.7119* N 19 s(2.30%)p42.39(97.31%)d 0.17(0.39%)
(49.32%) 0.7023* N 194 s(2.03%)p48.13(97.54%)d 0.21(0.44%)

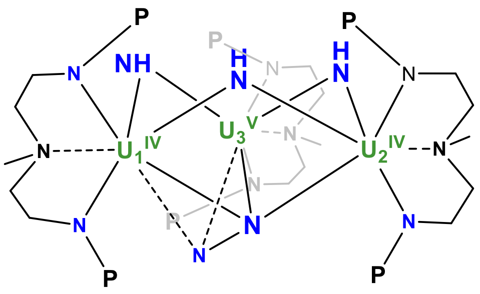

U1=2.296
U2=2.248
U3=1.304

BD (1) U 1 - N 16
(19.03%) 0.4362* U 1 s(3.50%)p
1.19(4.18%)d12.00(42.03%)f14.35(50.26%)g
(80.97%) 0.8998* N 16 s(40.68%)p 1.46(59.31%)d 0.00(0.01%)
BD (1) U 2 - N 16
(13.49%) 0.3673* U 2 s(9.91%)p 0.41(4.04%)d 5.90(58.41%)f
2.79(27.61%)g 0.00(0.03%)
(86.51%) 0.9301* N 16 s(38.61%)p 1.59(61.39%)d 0.00(0.00%)
BD (1) N 16 - H 161
(70.62%) 0.8404* N 16 s(20.57%)p 3.86(79.38%)d 0.00(0.05%)
(29.38%) 0.5420* H 161 s(99.90%)p 0.00(0.10%)

BD (1) U 2 - N 17
(14.50%) 0.3808* U 2 s(9.18%)p 0.31(2.85%)d 4.96(45.55%)f
4.62(42.38%)
(85.50%) 0.9247* N 17 s(38.11%)p 1.62(61.88%)d 0.00(0.01%)
BD (1) U 3 - N 17
(13.72%) 0.3704* U 3 s(11.00%)p 0.46(5.02%)d 6.25(68.78%)f
1.38(15.16%)g 0.00(0.04%)
(86.28%) 0.9289* N 17 s(39.16%)p 1.55(60.83%)d 0.00(0.01%)
BD (1) N 17 - H 162
(70.38%) 0.8390* N 17 s(19.67%)p 4.08(80.28%)d 0.00(0.05%)
(29.62%) 0.5442* H 162 s(99.91%)p 0.00(0.09%)

BD (1) U 1 - N 18
(17.55%) 0.4189* U 1 s(7.90%)p 1.01(7.99%)d 4.77(37.69%)f
5.87(46.37%)g 0.01(0.05%)
(82.45%) 0.9080* N 18 s(26.64%)p 2.75(73.35%)d 0.00(0.00%)
BD (2) U 1 - N 18
(20.32%) 0.4508* U 1
s(0.20%)p33.05(6.64%)d99.99(38.37%)f99.99(54.78%)

(79.68%) 0.8926* N 18 s(1.53%)p64.15(98.46%)d 0.01(0.01%)
BD (1) N 18 - H 163
(71.46%) 0.8454* N 18 s(20.29%)p 3.93(79.67%)d 0.00(0.04%)
(28.54%) 0.5342* H 163 s(99.90%)p 0.00(0.10%)

BD (1) U 1 - N 194
(20.70%) 0.4549* U 1 s(7.34%)p 1.92(14.12%)d 6.23(45.74%)f 4.47(32.78%)
(79.30%) 0.8905* N 194 s(39.96%)p 1.50(59.98%)d 0.00(0.05%)
BD (1) U 3 - N 194
(8.17%) 0.2859* U 3 s(9.24%)p 3.01(27.80%)d 4.06(37.53%)f 2.75(25.42%)g 0.00(0.01%)
(91.83%) 0.9583* N 194 s(38.10%)p 1.62(61.84%)d 0.00(0.07%)
BD (2) U 3 - N 194
(10.19%) 0.3192* U 3 s(3.92%)p 1.98(7.76%)d 6.62(25.95%)f15.92(62.35%)
(89.81%) 0.9477* N 194 s(0.14%)p99.99(99.78%)d 0.50(0.07%)

BD (1) U 2 - N 19
(11.29%) 0.3359* U 2 s(9.50%)p 0.64(6.10%)d 3.85(36.59%)f 5.03(47.79%)g 0.00(0.02%)
(88.71%) 0.9419* N 19 s(43.72%)p 1.29(56.27%)d 0.00(0.01%)
BD (1) N 19 - N 194
(53.38%) 0.7306* N 19 s(17.54%)p 4.69(82.35%)d 0.01(0.10%)
(46.62%) 0.6828* N 194 s(21.94%)p 3.55(77.92%)d 0.01(0.14%)

Cartesian coordinates of all optimized structures

77

460215-U-Cl2.log

U 4.659781 4.286939 17.152799
Cl 2.452807 2.962122 17.802635
Cl 3.409887 6.581498 17.676992
P 4.191754 4.850912 14.113988
P 6.053489 4.764446 19.976775
O 6.688006 5.712965 16.555171
N 4.498986 3.449117 15.084001
N 5.793945 3.429817 18.893387
N 5.616801 1.786440 16.813560
C 2.429669 4.770567 13.406224
C 6.126299 2.050560 19.204117
C 5.259964 4.624537 12.558990
C 4.583177 2.081100 14.601996
C 7.007426 1.789415 16.356172
C 5.112774 4.351940 21.572180
C 7.896328 4.613378 20.482090
C 1.427434 4.876575 14.556848
C 4.736457 1.160218 15.813678
C 5.498976 1.151818 18.137331
C 2.090667 3.608536 12.471217
C 3.645113 4.085116 21.242168
C 6.739594 4.579568 12.932162
C 8.224465 3.636721 21.612475
C 5.249524 5.516920 22.557077
C 4.984202 5.741852 11.549983
C 8.494239 5.997518 20.755708
C 6.530393 7.147926 16.336015
C 8.086690 5.355004 16.533668
C 7.880051 7.609710 15.810748
C 8.849513 6.674077 16.536753
H 2.381131 5.707054 12.830578
H 7.217771 1.897252 19.242706
H 5.739865 1.735206 20.185160
H 4.984615 3.663119 12.106343
H 3.688106 1.767526 14.049903
H 5.429779 1.952544 13.906783
H 7.084584 2.324491 15.407440
H 7.639276 2.295448 17.088546
H 7.387064 0.764480 16.213442
H 5.550492 3.450597 22.020510
H 8.346570 4.226518 19.556738
H 1.463365 3.993189 15.202103
H 1.621741 5.749018 15.187922
H 0.407211 4.962286 14.163386
H 5.113138 0.167860 15.512528
H 3.759921 1.031111 16.286430
H 4.431068 1.046379 18.344584
H 5.957171 0.148096 18.143688
H 2.806882 3.490468 11.651731
H 2.040061 2.662257 13.018212
H 1.103539 3.767887 12.019133
H 3.178547 4.956250 20.769094
H 3.518230 3.242374 20.558476
H 3.086994 3.865162 22.160800
H 7.062320 5.534085 13.362226
H 6.949056 3.795217 13.665398
H 7.357238 4.387453 12.046399
H 7.825729 3.981107 22.571987
H 7.827018 2.635132 21.428477
H 9.311961 3.545530 21.731444
H 6.284930 5.694767 22.864337
H 4.865129 6.444917 22.119051
H 4.669641 5.314978 23.465652
H 3.955869 5.723952 11.178803
H 5.162958 6.729030 11.992650
H 5.647395 5.645103 10.681711
H 8.288214 6.696773 19.939397
H 8.084326 6.440209 21.669786
H 9.581889 5.933101 20.886504
H 6.272019 7.605459 17.295070
H 5.698182 7.278507 15.642391
H 8.061015 8.666303 16.024654
H 7.945471 7.466036 14.726631
H 9.822106 6.587607 16.044923
H 9.015778 7.018098 17.562547
H 8.287030 4.726361 17.404511
H 8.278706 4.779137 15.620991

68

459989-U-N6.log

U 5.063114 4.376004 17.024219
P 4.256390 4.872959 14.130552

P 5.898392 4.795818 19.899271
N 2.039706 5.443425 18.267658
N 4.534238 3.439425 15.048312
N 5.719735 3.388291 18.917472
N 7.011540 5.436365 16.564124
N 5.647905 1.772081 16.813945
N 1.115672 5.832568 18.831354
N 3.000357 5.030806 17.669530
N 7.878834 6.162314 16.147951
N 8.715798 6.844306 15.751636
C 2.513181 4.810351 13.376733
C 6.096376 2.027143 19.222764
C 5.376021 4.775361 12.604234
C 4.654258 2.076125 14.575251
C 7.050243 1.701208 16.379118
C 5.006760 4.418635 21.527610
C 7.746687 4.882441 20.341952
C 1.473706 4.800531 14.497139
C 4.750893 1.176856 15.806644
C 5.477459 1.146122 18.138590
C 2.246961 3.702318 12.356341
C 3.567836 3.985160 21.252725
C 6.845506 4.759821 13.021806
C 8.213382 3.992096 21.493891
C 5.048087 5.639654 22.449792
C 5.089435 5.945857 11.659816
C 8.191388 6.336532 20.528912
H 2.446662 5.780341 12.863266
H 7.190299 1.893332 19.250323
H 5.722513 1.686327 20.200586
H 5.144614 3.834155 12.088057
H 3.791868 1.743231 13.981807
H 5.536386 1.940870 13.927540
H 7.166386 2.194769 15.412404
H 7.691575 2.218177 17.095986
H 7.389770 0.657650 16.285359
H 5.543253 3.589971 22.008444
H 8.191930 4.516183 19.406968
H 1.543688 3.884706 15.092730
H 1.606361 5.643875 15.179717
H 0.461635 4.854626 14.078744
H 5.071413 0.157108 15.535342
H 3.758338 1.105992 16.264859
H 4.400117 1.072088 18.322295
H 5.895446 0.125872 18.156547
H 2.985355 3.678854 11.549568
H 2.235376 2.717342 12.833057
H 1.262604 3.845801 11.893420
H 2.981925 4.799655 20.814954
H 3.520689 3.136078 20.565655
H 3.077617 3.693827 22.189480
H 7.139305 5.709941 13.478756
H 7.060361 3.966702 13.743581
H 7.486725 4.601334 12.146512
H 7.807056 4.324075 22.455282
H 7.928705 2.944684 21.355954
H 9.306697 4.027720 21.579463
H 6.068222 5.929258 22.718438
H 4.566369 6.503809 21.979036
H 4.510832 5.429153 23.382135
H 4.077193 5.915998 11.246244
H 5.216737 6.906090 12.173007
H 5.788406 5.931408 10.815118
H 7.898483 6.956240 19.676663
H 7.760278 6.784004 21.431065
H 9.282127 6.393257 20.627619

65
460545-U-N3.log
U 6.960249 3.218932 15.068346
P 5.536033 0.709403 14.030825
P 8.549356 5.654041 14.045330
N 8.465983 2.565290 19.392474
N 8.149805 2.675845 18.291790
N 7.820687 2.787778 17.136867
N 5.010412 2.042165 14.954423
N 7.029961 5.484193 14.813200
N 4.790276 4.535984 16.075544
C 9.428368 8.164341 15.259385
C 7.183610 6.188346 11.665177
C 6.024473 -0.819629 16.314905
C 4.570468 1.732631 11.609968
C 2.942604 0.198060 12.784624
C 4.414506 0.504165 12.506538
C 5.497007 -2.120485 14.214371
C 5.193150 -0.863830 15.032197
C 9.565705 7.007594 11.769973
C 8.269068 6.794873 12.553395

C 3.562550 3.792171 15.709639
C 3.791757 2.279817 15.699551
C 11.135782 6.300775 15.049498
C 9.656799 6.653002 15.233609
C 6.181869 6.496709 15.400159
C 4.776917 5.900878 15.503516
C 4.947203 4.588728 17.536426
H 11.741379 6.762282 15.839081
H 11.526655 6.657672 14.090583
H 11.298750 5.219512 15.091449
H 5.384853 -3.017895 14.834690
H 6.528116 -2.108555 13.841101
H 4.830156 -2.233036 13.354098
H 9.377566 7.609984 10.872730
H 9.988910 6.051134 11.441348
H 10.329985 7.526271 12.355738
H 9.998434 8.621292 16.078277
H 8.375698 8.422900 15.407507
H 9.762469 8.642076 14.332234
H 5.784724 -1.673385 16.959950
H 5.847684 0.096954 16.885400
H 7.096254 -0.863848 16.089743
H 6.945998 6.860267 10.831562
H 6.264862 5.995525 12.226389
H 7.517199 5.235250 11.236491
H 2.410192 -0.013173 11.848344
H 2.443162 1.052331 13.252617
H 2.809984 -0.669489 13.438047
H 4.102448 6.560430 16.075519
H 4.381714 5.816928 14.486487
H 3.297293 4.088710 14.690303
H 2.724872 4.069979 16.370924
H 4.006835 1.609812 10.677286
H 5.619128 1.909147 11.346968
H 4.196044 2.632601 12.110586
H 9.332386 6.236391 16.197499
H 7.916707 7.761260 12.937291
H 4.114660 5.135517 18.008180
H 5.885438 5.083471 17.797542
H 4.980852 3.577618 17.947908
H 3.864249 1.896295 16.731527
H 2.897651 1.809320 15.266533
H 4.128358 -0.856048 15.301589
H 6.104970 7.415341 14.795469
H 6.539210 6.816091 16.394008
H 4.862139 -0.354291 11.985860

65
460991-TS-release-N2.log
U 6.902177 3.268267 14.888000
P 5.516332 0.711826 14.012016
P 8.487547 5.688744 14.015216
N 9.772073 1.654533 16.212575
N 7.653096 2.949925 16.731347
N 8.866173 2.156575 15.733030
N 4.955975 2.094206 14.844486
N 4.788081 4.532606 16.073160
N 6.915582 5.533795 14.674276
C 7.279085 6.328070 11.575832
C 6.087286 -0.670037 16.364752
C 4.556347 1.564081 11.527207
C 2.912983 0.161068 12.837434
C 4.386355 0.409035 12.514412
C 5.485062 -2.099673 14.370478
C 5.209907 -0.793334 15.117700
C 3.546805 3.792097 15.761068
C 3.796608 2.285927 15.690750
C 5.051623 4.514270 17.520918
C 4.733104 5.921043 15.570383
C 6.131601 6.512105 15.393415
C 9.688059 7.032434 11.834869
C 8.338505 6.866934 12.535983
C 11.012705 6.193583 15.208659
C 9.541149 6.608002 15.303050
C 9.374789 8.127180 15.346262
H 4.808779 -0.495634 12.055099
H 6.571308 6.759375 16.374269
H 6.024235 7.468130 14.854938
H 4.153752 -0.766230 15.418600
H 2.881765 1.809146 15.310344
H 3.956972 1.876013 16.702001
H 5.129429 3.484562 17.874699
H 6.007538 4.995320 17.736143
H 4.250904 5.029725 18.075762
H 8.007318 7.840458 12.920325
H 9.135444 6.180652 16.230747
H 4.217205 2.507827 11.969501
H 5.602798 1.687517 11.227667

H 3.966445 1.391828 10.619112
H 2.748901 4.035230 16.482400
H 3.213816 4.116967 14.770097
H 4.259938 5.889366 14.583639
H 4.110220 6.555550 16.223157
H 2.772050 -0.650418 13.557872
H 2.444950 1.061764 13.247319
H 2.358792 -0.106822 11.928820
H 7.587090 5.362945 11.154579
H 6.318242 6.183243 12.077840
H 7.130410 7.020582 10.738612
H 7.147297 -0.767367 16.106436
H 5.967201 0.294990 16.865717
H 5.844803 -1.462348 17.083164
H 9.781169 8.607278 14.449477
H 8.327249 8.428713 15.439722
H 9.915805 8.542791 16.205665
H 10.441647 7.493499 12.479777
H 10.080302 6.063934 11.503245
H 9.583783 7.668044 10.947138
H 4.787246 -2.267296 13.544403
H 6.502437 -2.114207 13.961757
H 5.395931 -2.953471 15.052764
H 11.123892 5.105784 15.237493
H 11.481722 6.553676 14.286536
H 11.581462 6.612018 16.047923

63
461415-U-N.log
U 7.104434 3.163207 13.948724
P 5.278037 0.207968 13.773704
P 8.758087 6.091148 13.621828
N 7.923743 2.851106 15.544158
N 5.001389 1.824816 14.212365
N 5.017352 4.439800 15.381810
N 7.144455 5.654373 13.936443
C 7.741197 7.725290 11.585395
C 6.261932 -0.444401 16.292205
C 3.666689 0.367467 11.485275
C 2.366047 -0.489830 13.460467
C 3.736664 -0.438395 12.781578
C 5.037807 -2.315189 15.127144
C 5.081374 -0.809487 15.389267
C 3.782277 3.701640 15.131042
C 3.989712 2.189360 15.169793
C 5.390132 4.383948 16.789636
C 4.938393 5.817396 14.901995
C 6.316946 6.463442 14.786215
C 10.054870 8.310911 12.393818
C 8.660576 7.836136 12.799648
C 11.003426 6.090153 15.366360
C 9.533674 6.514362 15.345403
C 9.352788 7.919043 15.919547
H 4.033387 -1.468952 12.528084
H 6.726955 6.599404 15.807855
H 6.150356 7.495228 14.404350
H 4.146296 -0.507081 15.883170
H 2.995999 1.740405 14.979400
H 4.241997 1.890049 16.208150
H 5.507448 3.345061 17.097593
H 6.367776 4.843579 16.931070
H 4.630570 4.887900 17.429524
H 8.228831 8.556758 13.508730
H 8.981469 5.793489 15.965124
H 3.465038 1.421663 11.706256
H 4.622536 0.342502 10.951242
H 2.877343 -0.005706 10.812703
H 2.982355 4.003967 15.843290
H 3.444915 3.957959 14.119322
H 4.500907 5.788300 13.896652
H 4.272014 6.432864 15.546714
H 2.393494 -0.999747 14.430183
H 1.978204 0.519828 13.631257
H 1.626631 -1.016208 12.832558
H 8.174280 7.050192 10.836748
H 6.777614 7.291962 11.869266
H 7.577427 8.704344 11.106920
H 7.195087 -0.847479 15.880648
H 6.416504 0.639402 16.358106
H 6.132643 -0.858664 17.304766
H 9.894799 8.675037 15.335575
H 8.301320 8.221419 15.948390
H 9.740997 7.977690 16.949743
H 10.717081 8.434660 13.258189
H 10.526089 7.578170 11.726307
H 10.022073 9.274908 11.860935
H 4.135372 -2.625418 14.587296
H 5.903851 -2.625996 14.527776

H 5.072252 -2.885972 16.068511
H 11.101437 5.050386 15.040829
H 11.619924 6.711007 14.700987
H 11.431494 6.172126 16.378286

63
462767-UV-N.log
U 6.946715 3.267809 15.028898
P 8.464501 5.655410 14.002176
P 5.566450 0.739576 14.010610
N 6.878123 5.519343 14.637619
N 4.769778 4.543518 16.087744
N 4.973781 2.136775 14.803862
N 7.754764 2.954396 16.556012
C 9.324961 8.150580 15.249500
C 9.483049 6.630518 15.278190
C 10.954512 6.206294 15.246074
C 8.346124 6.776109 12.476224
C 9.709994 6.906784 11.795841
C 6.116519 6.510053 15.362510
C 4.719851 5.928300 15.568880
C 4.978052 4.540351 17.545075
C 3.794453 2.300728 15.628314
C 3.538689 3.802033 15.733875
C 5.174544 -0.740051 15.124505
C 5.449823 -2.064385 14.409876
C 4.502079 0.434953 12.466340
C 3.011882 0.216840 12.726996
C 4.734260 1.575304 11.474621
C 5.998135 -0.618473 16.407983
C 7.300868 6.206233 11.518691
H 11.501730 6.665519 16.078428
H 11.450982 6.516713 14.320177
H 11.057677 5.121032 15.335012
H 5.308963 -2.904728 15.100156
H 6.483547 -2.110528 14.047160
H 4.785198 -2.227769 13.556104
H 9.627617 7.509469 10.883153
H 10.101185 5.923960 11.508072
H 10.454739 7.386492 12.437695
H 9.838146 8.600446 16.108786
H 8.277007 8.461814 15.292331
H 9.766662 8.588957 14.348168
H 5.703272 -1.392547 17.126808
H 5.879509 0.357514 16.887598
H 7.066679 -0.742978 16.201295
H 7.171120 6.867435 10.653407
H 6.331262 6.084649 12.009514
H 7.609981 5.224061 11.140275
H 2.492883 -0.049516 11.797449
H 2.544891 1.130739 13.107700
H 2.823846 -0.584553 13.448074
H 4.108582 6.572554 16.222307
H 4.235581 5.890238 14.588519
H 3.221622 4.147789 14.745436
H 2.726622 4.024060 16.445552
H 4.187447 1.397026 10.540990
H 5.795250 1.684924 11.225857
H 4.384656 2.527874 11.888360
H 9.049737 6.245052 16.211503
H 8.014234 7.765347 12.817379
H 4.152278 5.055971 18.063347
H 5.922422 5.029262 17.790046
H 5.048715 3.514219 17.910255
H 3.934941 1.865039 16.631988
H 2.888963 1.832330 15.215307
H 4.107259 -0.683135 15.378108
H 6.003554 7.464108 14.821082
H 6.576333 6.757956 16.334407
H 4.929767 -0.482497 12.038106

66
461834-U-N3-K.log
U 6.926257 3.273234 14.985715
K 8.814432 0.077796 18.179005
P 5.533200 0.752691 14.023591
P 8.539027 5.682349 14.102222
N 9.074464 2.891798 19.102288
N 8.565846 2.818052 18.061891
N 8.051419 2.612010 16.993593
N 5.027604 2.033265 15.032985
N 4.780377 4.542151 16.036159
N 7.003047 5.516317 14.860925
C 7.197595 6.074991 11.681671
C 6.135454 -0.886597 16.208130
C 4.522326 1.854285 11.666804
C 2.923845 0.259287 12.808581
C 4.388301 0.591651 12.519434

C 5.503696 -2.094224 14.085701
C 5.241561 -0.871410 14.965874
C 3.545391 3.784225 15.722506
C 3.788703 2.274934 15.749259
C 5.001329 4.599131 17.489025
C 4.720313 5.906131 15.460363
C 6.108090 6.538737 15.358592
C 9.569849 6.927530 11.783432
C 8.262062 6.737671 12.555535
C 11.102525 6.387448 15.113237
C 9.616907 6.726665 15.269690
C 9.366375 8.234444 15.229625
H 4.838250 -0.241473 11.961437
H 6.432990 6.926082 16.337565
H 6.030532 7.413124 14.694957
H 4.194844 -0.875527 15.295786
H 2.912513 1.779253 15.312618
H 3.852360 1.914944 16.789736
H 5.062295 3.587065 17.898571
H 5.941344 5.112577 17.707885
H 4.186126 5.131412 18.002259
H 7.893437 7.717186 12.886208
H 9.284869 6.344561 16.245542
H 4.147316 2.733854 12.202347
H 5.563777 2.045835 11.385880
H 3.942714 1.758884 10.742059
H 2.723610 4.074241 16.396254
H 3.252751 4.055472 14.703775
H 4.323142 5.803311 14.445862
H 4.027155 6.544770 16.030659
H 2.805810 -0.624706 13.442274
H 2.413891 1.096113 13.295592
H 2.389626 0.060273 11.872086
H 7.548324 5.107315 11.301492
H 6.266068 5.906625 12.230459
H 6.970727 6.703002 10.812787
H 7.187933 -0.947801 15.892418
H 5.990243 0.024831 16.800949
H 5.911821 -1.759430 16.834682
H 9.699692 8.677383 14.285737
H 8.310398 8.486204 15.363738
H 9.926831 8.730318 16.031146
H 10.318834 7.481686 12.355397
H 10.007942 5.963353 11.501030
H 9.385970 7.488445 10.860069
H 4.790159 -2.172462 13.261472
H 6.511238 -2.065536 13.653166
H 5.421642 -3.017552 14.671279
H 11.283040 5.310757 15.191758
H 11.499929 6.720052 14.149078
H 11.689570 6.884397 15.894165

66
461835-TS-release-K-N2.log
U 6.862974 3.313798 14.964509
K 8.833258 1.703720 18.950394
P 8.461625 5.675143 14.080944
P 5.554067 0.783523 14.093388
N 6.928207 5.547329 14.845381
N 4.717876 4.590518 16.041658
N 7.678445 2.803733 16.851897
N 8.838453 2.083822 15.805709
N 9.771530 1.550437 16.220817
N 4.950807 2.145985 14.942235
C 7.127667 6.228556 11.687333
C 8.240545 6.791336 12.571026
C 9.552281 6.923175 11.793314
C 9.602911 6.599270 15.282181
C 9.439986 8.119331 15.302370
C 6.095048 6.569967 15.442721
C 4.687446 5.984545 15.542489
C 4.936931 4.565976 17.495732
C 3.705440 2.361021 15.651975
C 3.474302 3.870031 15.686656
C 5.232161 -0.754663 15.143909
C 5.619388 -2.027705 14.388582
C 4.519991 0.507052 12.531112
C 3.053692 0.134592 12.754117
C 4.661261 1.725742 11.618326
C 5.996671 -0.635695 16.463358
C 11.063602 6.176377 15.096508
H 5.030996 -0.337591 12.048423
H 6.467622 6.868447 16.435540
H 6.040267 7.491110 14.843928
H 4.156164 -0.774666 15.360298
H 2.836285 1.890192 15.175033
H 3.751920 1.951432 16.674247
H 4.978244 3.532045 17.847121

H 5.890214 5.042361 17.738210
H 4.132101 5.090885 18.032937
H 7.938298 7.779721 12.940705
H 9.252923 6.194702 16.242466
H 4.217958 2.616067 12.078489
H 5.710592 1.942910 11.389469
H 4.145067 1.555294 10.667287
H 2.650458 4.138998 16.366170
H 3.192375 4.189403 14.678771
H 4.263451 5.954073 14.533975
H 4.030856 6.614487 16.163269
H 2.932428 -0.720643 13.425022
H 2.486991 0.974530 13.167929
H 2.583185 -0.131330 11.800339
H 7.406044 5.249323 11.278827
H 6.187425 6.115104 12.235122
H 6.944703 6.895719 10.837796
H 7.076849 -0.703467 16.287767
H 5.796680 0.318137 16.961969
H 5.713444 -1.451042 17.139703
H 9.791595 8.578430 14.373122
H 8.401154 8.425609 15.455938
H 10.034539 8.550325 16.116643
H 10.340073 7.408893 12.375443
H 9.924906 5.943808 11.472565
H 9.395790 7.526924 10.892374
H 5.004027 -2.191418 13.499674
H 6.668657 -1.998692 14.072547
H 5.495192 -2.904085 15.034800
H 11.177342 5.088464 15.130187
H 11.472745 6.522437 14.142298
H 11.686327 6.607027 15.889317

64
462163-U-NK.log
U 6.910565 3.321320 15.131284
K 8.967048 0.781844 17.808157
P 5.607274 0.749871 14.153040
P 8.505358 5.686282 14.136801
N 7.757776 2.845295 16.814007
N 6.965702 5.542078 14.900838
N 4.737524 4.585886 16.117086
N 5.008535 2.114604 14.998296
C 3.111252 0.352722 12.724184
C 5.386435 -2.077006 14.395628
C 9.324314 8.259234 15.218999
C 5.929720 -0.762585 16.482357
C 5.143732 -0.780906 15.170196
C 4.693244 5.945818 15.529396
C 6.086331 6.565731 15.425114
C 4.932748 1.719634 11.627043
C 4.618202 0.541599 12.550247
C 4.895686 4.660824 17.579856
C 3.517357 3.819460 15.763158
C 3.781589 2.314566 15.745711
C 11.056890 6.406674 15.149675
C 9.571406 6.752410 15.293059
C 9.532124 6.872161 11.789229
C 8.226463 6.713635 12.571814
C 7.146833 6.048744 11.719124
H 11.640470 6.917270 15.924430
H 11.460258 6.721329 14.181917
H 11.233062 5.331088 15.249043
H 5.197411 -2.946029 15.036426
H 6.423664 -2.145714 14.045722
H 4.733024 -2.169393 13.524293
H 9.350421 7.422420 10.859170
H 9.953963 5.897622 11.518185
H 10.292558 7.423449 12.348837
H 9.887866 8.771126 16.008137
H 8.269556 8.517157 15.350541
H 9.656571 8.680453 14.264754
H 5.570076 -1.542905 17.163268
H 5.844694 0.207418 16.985387
H 6.990453 -0.966033 16.275067
H 6.930148 6.658758 10.835053
H 6.213385 5.916165 12.274168
H 7.475184 5.064343 11.363406
H 2.639490 0.157459 11.754046
H 2.647163 1.256040 13.132057
H 2.860339 -0.485395 13.381296
H 4.006940 6.597535 16.092684
H 4.298214 5.844214 14.513975
H 3.229899 4.114606 14.749807
H 2.684071 4.074690 16.435868
H 4.434611 1.594642 10.659363
H 6.007652 1.812110 11.436925
H 4.577372 2.662248 12.059211

H 9.232110 6.385896 16.271938
H 7.872480 7.703195 12.887627
H 4.060802 5.208562 18.043539
H 5.832522 5.163089 17.830676
H 4.936150 3.654314 18.000974
H 3.870749 1.927903 16.774621
H 2.905417 1.818034 15.307455
H 4.075345 -0.693281 15.405163
H 6.015774 7.452707 14.778070
H 6.430658 6.924920 16.407687
H 5.047841 -0.365318 12.102767

65
459629-U-NK2.log
U 4.198970 4.455147 17.361944
K 0.765377 3.405648 16.278250
K 0.928976 4.081536 20.158623
P 6.151368 4.985614 19.807073
P 3.853406 4.924856 14.225296
N 2.496745 4.205904 18.068376
N 5.049008 1.911939 17.011963
N 5.580110 3.588021 19.025724
N 3.688789 3.519392 15.183371
C 8.702457 6.268393 19.743032
C 5.658376 6.018415 12.318691
C 6.146163 6.061143 22.437073
C 8.716421 3.983777 20.836486
C 6.651075 5.077564 14.418621
C 4.223625 4.564853 21.793452
C 3.017868 3.696730 11.695110
C 4.944103 1.363384 18.376795
C 4.108623 1.296856 16.064619
C 1.242333 4.749791 13.132264
C 8.064364 4.876763 19.780034
C 5.724512 4.816064 21.655850
C 6.424692 1.788732 16.522563
C 3.956249 2.149543 14.795174
C 5.560313 4.901892 13.361922
C 5.783077 2.198842 19.357795
C 2.704928 4.810761 12.695157
H 9.781481 6.196512 19.556453
H 8.573955 6.801829 20.691314
H 8.264250 6.890457 18.956108
H 6.682634 6.098023 11.933649
H 5.399660 6.989931 12.758093
H 5.000486 5.854600 11.460847
H 5.842897 5.981668 23.488722
H 5.676030 6.961572 22.024158
H 7.228720 6.216649 22.421562
H 9.792831 3.884957 20.643768
H 8.290144 2.976303 20.848714
H 8.610887 4.401900 21.843413
H 7.650078 5.026806 13.967434
H 6.604650 4.302881 15.190782
H 6.562299 6.053312 14.912536
H 3.950152 4.411336 22.845441
H 3.918031 3.685796 21.217176
H 3.657090 5.427364 21.418715
H 2.354127 3.761699 10.822542
H 2.870211 2.709262 12.143586
H 4.045585 3.741354 11.324348
H 5.249519 0.301788 18.406600
H 3.889115 1.427411 18.664260
H 3.139796 1.236335 16.574652
H 4.410216 0.266042 15.803913
H 0.569432 4.929208 12.285100
H 1.013738 5.497495 13.901188
H 0.996744 3.751206 13.515906
H 8.215722 4.412467 18.794961
H 6.262961 3.943689 22.047631
H 6.767243 0.740646 16.547307
H 7.094643 2.393997 17.136511
H 6.491445 2.141947 15.492356
H 4.855450 2.041237 14.164004
H 3.141719 1.688650 14.204858
H 5.699463 3.931362 12.867115
H 5.460612 1.931049 20.379523
H 6.841787 1.892703 19.290632
H 2.858072 5.781853 12.204498

80
460924-TS-KH.log
U 3.988676 5.247173 15.862143
K 0.316082 3.210994 15.435284
K 1.312874 4.339535 19.050404
P 6.106697 5.399723 18.312479
P 3.431676 6.440933 12.989672
N 2.287622 4.627601 16.552235

N 5.153978 3.000223 14.887464
N 5.530346 4.183738 17.263958
N 3.746068 4.868097 13.571542
C 8.479303 6.972504 18.206147
C 4.520089 8.256560 11.110716
C 6.460711 5.713963 21.115883
C 8.904224 4.527382 18.696870
C 6.141388 6.958413 12.528133
C 4.606655 4.187573 20.354124
C 2.065773 5.692388 10.500059
C 5.209133 2.119656 16.064104
C 4.240975 2.523505 13.841128
C 0.718474 5.832980 12.627556
C 7.994829 5.535022 17.992425
C 6.026437 4.679136 20.077338
C 6.493878 3.226705 14.343159
C 3.942173 3.643189 12.831602
C 4.816615 6.906825 11.768270
C 5.975526 2.809468 17.199559
C 1.902029 6.401937 11.845235
C 0.178993 7.270967 18.265942
C 1.583867 7.413442 18.216015
C 2.309742 7.379163 16.935724
C 2.264361 7.439907 19.453855
C 1.584024 7.320446 20.665049
C 0.193112 7.166763 20.690373
C -0.502384 7.153106 19.477581
H 9.518096 7.087915 17.871628
H 8.446475 7.259907 19.263013
H 7.864775 7.690143 17.653491
H 5.361781 8.575277 10.483462
H 4.363344 9.034501 11.867942
H 3.630757 8.229629 10.474346
H 6.371658 5.308393 22.131739
H 5.834784 6.612516 21.061563
H 7.499501 6.028990 20.981556
H 9.932317 4.612769 18.321241
H 8.580837 3.494560 18.540619
H 8.945935 4.702234 19.777171
H 6.980626 7.145983 11.847270
H 6.339593 6.021271 13.057016
H 6.132018 7.764919 13.271783
H 4.547932 3.695553 21.333487
H 4.280013 3.477750 19.586719
H 3.910796 5.036540 20.364810
H 1.141770 5.770288 9.912094
H 2.278922 4.627245 10.631228
H 2.870576 6.118629 9.894588
H 5.663552 1.142736 15.820493
H 4.175324 1.943847 16.384163
H 3.308620 2.234878 14.341808
H 4.633815 1.626258 13.330802
H -0.212716 5.929008 12.055809
H 0.584411 6.340747 13.588676
H 0.876824 4.766388 12.826959
H 8.026630 5.329938 16.912487
H 6.706440 3.818854 20.121078
H 6.981333 2.278626 14.060831
H 7.115911 3.735244 15.082598
H 6.432040 3.858559 13.456307
H 4.760685 3.716145 12.093775
H 3.057428 3.326325 12.253739
H 4.878597 6.129918 10.995054
H 5.784586 2.238906 18.125079
H 7.059994 2.720294 17.015413
H 1.700710 7.466290 11.658484
H 3.345714 7.557547 19.451323
H 2.141631 7.359352 21.598321
H -0.338176 7.084826 21.634236
H -1.585177 7.052583 19.474283
H -0.381078 7.255034 17.333558
H 1.744743 7.815122 16.107817
H 3.280076 7.883472 17.013987
H 2.194720 5.981063 16.731659

65
459688-U-NKH.log
U 4.542960 4.389877 17.195717
K 1.150111 2.666999 18.928876
P 6.021058 4.824328 19.795506
P 4.183929 4.934542 14.257580
N 2.680502 4.769747 17.689062
N 5.382713 1.806883 16.920404
N 5.489021 3.394227 19.024239
N 4.409314 3.472188 15.110643
C 8.603757 6.018896 19.858723
C 5.345579 6.361128 12.125766
C 5.632622 6.219333 22.207098

C 8.394992 3.911130 21.244607
C 6.906576 5.028434 13.588293
C 3.846508 4.558190 21.542826
C 2.606642 3.957214 12.000694
C 5.204900 1.153102 18.226911
C 4.568511 1.198796 15.855669
C 1.418595 4.499287 14.165828
C 7.912070 4.659258 20.001727
C 5.342840 4.860048 21.566762
C 6.798535 1.834413 16.544397
C 4.527253 2.115403 14.625670
C 5.529946 5.070795 12.927742
C 5.805256 2.020282 19.340924
C 2.580165 4.857161 13.238093
H 1.754553 5.195169 17.746734
H 9.693613 5.896515 19.832848
H 8.372127 6.682883 20.698550
H 8.299958 6.532692 18.941233
H 6.147046 6.473409 11.385625
H 5.378769 7.238915 12.782122
H 4.394932 6.386569 11.585057
H 5.230780 6.255975 23.226706
H 5.164191 7.029472 21.636667
H 6.704271 6.432784 22.270860
H 9.479165 3.749406 21.192956
H 7.919623 2.931873 21.353100
H 8.200415 4.480130 22.159718
H 7.701600 5.086034 12.835113
H 7.053737 4.108270 14.161248
H 7.039932 5.875729 14.272651
H 3.430253 4.611023 22.556242
H 3.649216 3.556987 21.148263
H 3.304445 5.273748 20.914169
H 1.650439 4.026508 11.466945
H 2.750050 2.906450 12.270478
H 3.393244 4.232188 11.291878
H 5.637371 0.137998 18.237113
H 4.124973 1.050126 18.393975
H 3.551314 1.088116 16.250061
H 4.934211 0.192451 15.588412
H 0.463364 4.599978 13.636710
H 1.393616 5.139208 15.052448
H 1.501598 3.465900 14.517614
H 8.166951 4.059916 19.115917
H 5.853703 4.075500 22.138508
H 7.208780 0.816616 16.437416
H 7.374276 2.366614 17.304736
H 6.919648 2.361473 15.596492
H 5.429134 1.968888 14.006833
H 3.678996 1.796999 14.001966
H 5.433062 4.208230 12.256149
H 5.381089 1.683859 20.301601
H 6.892062 1.845876 19.411241
H 2.446264 5.898022 12.910038

80
460980-product-KH.log
U 4.071113 5.220065 15.881172
K 2.376782 6.553617 19.096551
K 0.652991 3.016160 17.732916
P 2.501514 6.029896 13.206127
P 5.348645 4.600189 18.963008
N 3.774269 4.937872 13.603077
N 5.554745 4.109975 17.316804
N 5.961113 3.699166 14.611972
N 2.242843 5.075917 16.768955
C 1.834527 9.513566 17.803738
C 2.358955 9.835372 19.063977
C 3.691041 9.501182 19.330350
C 4.465506 8.838139 18.378123
C 4.730015 7.698135 16.145699
C 3.949567 8.469170 17.110142
C 2.605953 8.852034 16.854027
C 1.065800 5.055969 12.401345
C 6.632213 3.210964 16.940544
C 3.126114 7.056152 11.730994
C 4.572902 4.213431 12.634600
C 7.075198 4.600063 14.312006
C 5.312377 2.980174 19.969299
C 7.015995 5.386825 19.492836
C 0.489224 4.074941 13.422957
C 5.367053 3.147570 13.384080
C 6.355878 2.642326 15.553091
C 1.325180 4.359228 11.064132
C 4.235730 2.048659 19.416924
C 4.352611 7.866465 12.148014
C 8.159759 4.440771 19.864157
C 5.068626 3.270634 21.451564

C 2.021251 7.972428 11.201246
C 6.797367 6.428990 20.595205
H 1.393082 5.445193 16.325497
H 5.808745 7.759903 16.337642
H 4.530564 8.010947 15.113800
H 2.170664 8.601618 15.889200
H 0.810406 9.787873 17.559312
H 1.759375 10.366092 19.797817
H 4.137861 9.774999 20.284202
H 5.504894 8.609364 18.601076
H 0.317528 5.845573 12.239827
H 7.613674 3.715059 16.941301
H 6.741829 2.353095 17.623962
H 3.423607 6.358947 10.936450
H 3.978948 3.702325 11.863918
H 5.260613 4.878020 12.081699
H 6.743127 5.385495 13.628944
H 7.429871 5.078008 15.229189
H 7.920707 4.067091 13.846731
H 6.288806 2.490634 19.863198
H 7.289851 5.923331 18.573179
H 1.226604 3.309191 13.688901
H 0.192529 4.584092 14.345394
H -0.394815 3.566600 13.018223
H 6.134636 2.687242 12.737327
H 4.673933 2.357310 13.693698
H 5.512455 1.945258 15.622938
H 7.228089 2.075495 15.181072
H 1.775489 5.020509 10.317977
H 1.982497 3.492806 11.187545
H 0.382931 3.986349 10.641600
H 3.236176 2.466951 19.592610
H 4.347882 1.888501 18.340266
H 4.269227 1.073948 19.919157
H 4.087994 8.622847 12.895054
H 5.130549 7.230847 12.581915
H 4.783470 8.389725 11.285534
H 7.952495 3.903259 20.795779
H 8.363035 3.696314 19.090311
H 9.084866 5.009684 20.024465
H 5.868920 3.865045 21.901759
H 4.126149 3.813333 21.595994
H 4.995852 2.335851 22.020865
H 1.176059 7.414346 10.787595
H 1.636412 8.623253 11.995466
H 2.406580 8.620477 10.404347
H 6.006586 7.137159 20.331075
H 6.518002 5.961149 21.545690
H 7.715987 7.001565 20.775297

195
U3-N4-s-4r.log
U -2.887825 5.126523 0.745954
U -2.739863 7.324120 3.500525
U -2.634742 3.740203 3.988824
K 0.423678 5.456302 2.596647
K -6.141649 5.363413 3.001897
P 0.582387 8.443203 4.219991
P 0.665722 2.450440 4.302632
P -5.812334 7.185842 0.019813
P -5.574089 1.926493 2.845785
P -5.533382 7.041991 5.868613
P 0.222505 5.321933 -0.847735
N -0.918646 2.406979 4.997864
N -4.835180 2.617835 4.247257
N -4.919101 7.955037 4.531828
N -3.195109 3.000192 6.563850
N -3.504633 9.886445 2.919142
N -1.056113 8.958070 4.007232
N -1.992227 6.994674 1.376252
N -1.836660 5.684815 4.722847
N -1.846733 3.576849 1.850561
N -3.572131 5.361932 2.669159
N -3.774133 3.288987 -1.110111
N -5.153151 5.593639 -0.154798
N -1.424473 4.812650 -1.078275
C -8.305511 7.891757 1.220925
C 3.104169 9.444726 3.266386
C 1.508653 4.171443 6.346150
C 2.333650 8.277660 6.447887
C 2.262844 0.590288 2.874692
C 3.303866 2.657409 5.448969
C 1.194543 9.356637 1.634229
C -0.064587 -0.154057 3.416121
C 1.594557 9.607492 3.087263
C 0.002333 9.036158 6.946901
C 1.819381 2.782338 5.792524
C 1.145996 9.100024 5.937848

C	1.144463 0.629437 3.920786
C	-1.095187 1.707023 6.261035
C	2.767246 4.099225 -0.780328
C	1.973603 6.905314 -2.533830
C	0.840475 2.529946 -0.718018
C	1.351462 3.840798 -1.313442
C	-8.197049 0.946490 3.924336
C	-7.725743 6.894696 0.210909
C	-8.218276 2.385910 1.862138
C	-8.148803 5.935435 6.186191
C	-7.485059 2.073550 3.171629
C	-2.668137 3.905072 7.585069
C	-7.463298 7.240032 5.761544
C	-8.121646 8.433552 6.457245
C	-3.852474 -0.286883 3.069049
C	-1.316615 10.389059 3.981720
C	-2.817397 10.659125 3.962843
C	-5.601403 7.219986 8.683830
C	-5.313296 2.157816 5.543942
C	-5.467795 9.292878 4.348836
C	-4.658022 2.932157 6.680573
C	-4.965320 9.932372 3.061466
C	-3.133098 10.347593 1.581412
C	-5.339722 0.029899 2.946305
C	-3.660928 8.323455 7.527352
C	-2.569782 1.673494 6.651959
C	-5.156164 8.029471 7.463863
C	-8.600370 6.844160 -1.044369
C	-0.387660 7.700263 -2.187915
C	-6.262551 9.456189 -1.600739
C	0.517694 6.479920 -2.345277
C	-5.241140 3.302891 -1.054818
C	-3.281937 1.928105 -0.903647
C	-3.275658 3.864792 -2.365396
C	-5.748127 8.017868 -1.702959
C	-5.815881 4.712697 -1.106396
C	-4.312133 8.004045 -2.218671
C	-1.778968 4.148019 -2.322346
C	-5.931652 -0.635921 1.701994
H	-9.345189 7.643926 1.474320
H	-8.305627 8.910259 0.816956
H	-7.726590 7.928651 2.150783
H	-7.759192 5.890404 0.663192
H	-7.535838 2.966456 3.815195
H	-7.620090 7.369682 4.678579
H	3.446662 9.785762 4.246978
H	3.643689 10.031710 2.511773
H	3.413871 8.398390 3.145866
H	2.768816 4.098384 0.317012
H	1.824093 4.953170 5.643973
H	2.038665 4.348726 7.290731
H	0.437028 4.305597 6.517315
H	2.027882 7.243130 6.639203
H	2.714211 8.688874 7.391921
H	3.168440 8.244585 5.742627
H	1.912069 1.009010 1.924867
H	2.579916 -0.443037 2.683224
H	3.148841 1.157291 3.172023
H	3.594934 1.628111 5.222364
H	3.919009 2.989219 6.295438
H	3.573727 3.282559 4.587950
H	1.525481 8.365365 1.298895
H	1.654192 10.095947 0.965899
H	0.109762 9.396341 1.502204
H	-0.879502 -0.171207 4.142156
H	-0.462128 0.289569 2.495942
H	0.754274 2.604255 0.372155
H	1.330212 10.639368 3.354129
H	-1.589926 4.022072 7.468661
H	-0.850778 9.647632 6.647088
H	0.342124 9.379200 7.932658
H	-0.362108 8.009222 7.062475
H	-0.909632 10.917591 4.861872
H	1.584106 2.032898 6.559686
H	1.458699 10.146888 5.817123
H	1.505532 0.170048 4.851725
H	-0.523133 2.172694 7.081495
H	-0.750857 0.657919 6.229372
H	3.190579 5.051674 -1.105411
H	3.450889 3.303729 -1.103579
H	2.392100 7.332222 -1.614000
H	2.042272 7.678544 -3.309569
H	2.613932 6.076628 -2.848166
H	1.541588 1.713850 -0.935279
H	1.387498 3.752786 -2.408076
H	0.217503 -1.190024 3.188030
H	-8.223331 0.021101 3.340384
H	-9.240193 1.224751 4.127538

H -7.725096 0.718338 4.883116
H -7.760091 3.218033 1.314971
H -9.272219 2.636233 2.043950
H -8.202795 1.525081 1.184306
H -7.730279 5.057142 5.680743
H -9.226703 5.965873 5.977350
H -8.032191 5.756201 7.260794
H -2.874915 3.529996 8.603186
H -3.129812 4.887155 7.464952
H -8.040049 8.361605 7.546657
H -9.193470 8.471894 6.219336
H -7.682496 9.387826 6.156146
H -3.304488 0.032333 2.174962
H -3.400144 0.226886 3.921658
H -0.854871 10.884804 3.110502
H -5.450553 -0.261202 0.790820
H -3.235022 10.355838 4.927368
H -3.012536 11.741101 3.842154
H -6.680188 7.036661 8.699110
H -5.346638 7.744138 9.613760
H -5.102520 6.244079 8.707801
H -5.159757 1.073151 5.692730
H -6.398608 2.311112 5.676058
H -5.258177 9.959697 5.205919
H -6.567592 9.291295 4.255385
H -5.022246 3.965943 6.656072
H -4.954635 2.492069 7.652106
H -5.375147 9.372987 2.212453
H -5.333426 10.974149 2.992529
H -2.056734 10.254411 1.430695
H -3.420682 11.402312 1.424445
H -3.636206 9.726096 0.837483
H -3.075843 7.396671 7.534700
H -3.409583 8.885782 8.435520
H -3.326943 8.903455 6.663049
H -3.098577 1.011918 5.959945
H -2.687086 1.250841 7.667374
H -5.702943 8.980433 7.447610
H -1.434744 7.606010 0.779623
H -1.236387 5.861876 5.529717
H -1.218010 2.868441 1.471893
H -8.659994 7.820486 -1.535198
H -9.627545 6.557164 -0.781111
H -8.237668 6.125612 -1.783579
H -1.422060 7.405514 -1.994061
H -0.368752 8.319671 -3.093426
H -0.054931 8.327842 -1.352178
H -0.137281 2.241345 -1.106148
H -7.306558 9.514555 -1.278503
H -6.193234 9.962799 -2.571810
H -5.665948 10.034270 -0.885483
H 0.207115 5.925055 -3.240702
H -5.522581 2.834659 -0.104603
H -5.671463 2.693105 -1.871964
H -2.193545 1.928790 -0.838578
H -3.593643 1.255349 -1.721973
H -3.675961 1.548463 0.041732
H -3.798665 4.811577 -2.520240
H -3.517829 3.209608 -3.222374
H -6.381295 7.461043 -2.404178
H -5.773695 5.095052 -2.143048
H -6.892732 4.597913 -0.894757
H -3.649458 8.566954 -1.551235
H -4.249270 8.462908 -3.213453
H -3.914556 6.987635 -2.286944
H -1.223839 3.206222 -2.464622
H -1.552341 4.755141 -3.214800
H -3.689125 -1.365086 3.190388
H -7.007304 -0.463536 1.598804
H -5.777281 -1.722017 1.731386
H -5.857233 -0.350898 3.835308
192
Complex trimere
U -9.857168 5.972538 4.749631
U -9.233051 9.783394 6.312049
U -6.351242 8.049966 3.820800
P -7.119099 10.620431 8.643997
P -12.256591 6.275101 2.664344
P -10.399664 4.833627 7.892764
P -10.280047 11.995402 4.169621
P -6.890254 8.199704 0.484111
P -4.495349 5.872636 5.091085
N -9.631355 4.362428 6.409697
N -9.058278 3.514432 3.834943
N -11.242822 4.995676 3.202696
N -10.106246 7.799002 6.022528
N -8.165114 6.746571 3.602257
N -10.639823 11.530385 5.791889

N	-11.468763 10.053881 7.971914
N	-8.777099 10.137246 8.535677
N	-7.264529 9.374445 5.292867
N	-5.782833 8.765703 1.696106
N	-4.437081 10.073246 3.788216
N	-4.345387 7.558235 4.816765
C	-12.546130 10.591798 3.315552
C	-8.241084 9.569060 -1.658600
C	-9.087130 14.210263 5.476147
C	-4.684505 6.565973 -0.238108
C	-10.725287 6.086627 0.268760
C	-8.787603 3.360654 9.907419
C	-5.143396 4.454719 7.466446
C	-6.391422 9.476074 11.278135
C	-7.947059 13.271724 9.075133
C	-14.258158 5.381028 4.397068
C	-12.551597 3.107768 7.276216
C	-5.636310 12.691059 9.860889
C	-7.076534 12.193305 9.714973
C	-4.823699 9.103764 9.334219
C	-6.280798 9.306582 9.761574
C	-12.687421 9.357535 7.555119
C	-8.242758 3.543010 2.619802
C	-15.105879 6.497998 2.305657
C	-14.024735 5.598124 2.903865
C	-12.593151 7.742806 0.267714
C	-12.147137 6.360124 0.755627
C	-9.070707 14.254328 2.973029
C	-9.877588 13.867068 4.216514
C	-12.303457 3.795638 9.675211
C	-11.557701 3.404506 8.395892
C	-9.116834 5.841567 10.220905
C	-8.997263 4.702828 9.203487
C	-9.001825 3.059776 6.285675
C	-8.277469 2.948863 4.944843
C	-10.326209 2.802831 3.609880
C	-11.275749 3.624987 2.735891
C	-11.961876 12.602115 1.916068
C	-11.989515 12.013981 3.327004
C	-11.759096 12.056352 6.547280
C	-11.682130 11.509721 7.970895
C	-11.026796 9.548952 9.279110
C	-9.659350 10.098748 9.686931
C	-6.676093 6.465935 -1.739961
C	-5.808066 7.383108 -0.871356
C	-8.205040 10.659226 0.609543
C	-7.422279 9.809979 -0.390482
C	-4.729839 9.707528 1.355779
C	-3.745176 9.813660 2.516376
C	-4.972292 11.435776 3.820746
C	-3.566652 9.845774 4.951953
C	-3.186791 8.372563 5.114243
C	-2.862489 5.540458 7.522503
C	-4.289118 5.627332 6.977352
C	-2.788520 5.428055 2.897408
C	-2.946580 5.038993 4.364304
C	-3.043284 3.519112 4.513838
H	-8.256000 5.847877 10.902129
H	-10.017359 5.743388 10.838603
H	-9.159619 6.814577 9.722393
H	-8.118588 4.905846 8.577580
H	-4.748531 6.554472 7.344812
H	-6.844793 8.410333 9.470562
H	-11.722020 13.669080 1.914336
H	-12.944787 12.490765 1.440773
H	-11.232270 12.087651 1.280113
H	-11.895660 8.512353 0.615345
H	-9.150309 10.175350 0.879743
H	-8.441320 11.642820 0.184753
H	-7.652439 10.814477 1.540358
H	-8.110786 13.725856 2.962371
H	-8.858241 15.331113 2.968271
H	-9.583508 14.014271 2.037974
H	-7.107226 5.662862 -1.131262
H	-6.073726 5.997749 -2.528971
H	-7.505320 6.987652 -2.223569
H	-7.648505 9.114846 -2.457599
H	-8.629653 10.519952 -2.044974
H	-9.103396 8.920986 -1.462537
H	-11.942070 9.936160 2.680389
H	-13.572413 10.579728 2.927596
H	-12.552260 10.143239 4.312673
H	-4.028802 7.168970 0.393553
H	-5.097290 5.767347 0.389964
H	-10.006220 6.775607 0.725359
H	-12.635939 12.647261 3.950717
H	-5.700075 11.575422 3.019328
H	-9.643936 13.992297 6.389820

H -8.816987 15.273945 5.482390
H -8.155298 13.633439 5.517624
H -11.757743 13.157593 6.611122
H -6.501992 10.341154 -0.669835
H -10.826575 14.421591 4.222685
H -5.372790 8.173890 -1.498459
H -5.128806 10.707880 1.118467
H -4.155926 9.401953 0.465547
H -13.589392 8.023998 0.617532
H -12.603967 7.776616 -0.829152
H -15.032116 7.522180 2.690340
H -16.101835 6.120994 2.570577
H -15.056791 6.540434 1.214178
H -10.665490 6.227550 -0.818063
H -12.822734 5.595105 0.347878
H -4.072568 6.088022 -1.013602
H -2.359043 4.622195 7.201563
H -2.876659 5.529091 8.620100
H -2.241275 6.385162 7.211823
H -6.177732 4.547210 7.122139
H -5.153801 4.412422 8.562852
H -4.758886 3.492314 7.109888
H -4.747897 8.913920 8.259295
H -4.378474 8.250910 9.862348
H -4.206003 9.980653 9.559472
H -4.173998 12.189560 3.709970
H -5.489889 11.605178 4.766964
H -5.830523 10.346237 11.635059
H -5.973379 8.598035 11.787967
H -7.425046 9.587735 11.616138
H -3.648936 5.094173 2.307444
H -2.702752 6.510124 2.768448
H -12.728826 11.787311 6.096341
H -3.928520 3.130920 3.996688
H -10.822582 11.968188 8.464904
H -12.588022 11.776867 8.545108
H -4.989705 11.972242 10.371583
H -5.608144 13.624161 10.437512
H -5.193409 12.897397 8.879238
H -2.331642 8.125325 4.461609
H -2.819763 8.247690 6.144917
H -9.779183 11.097069 10.141474
H -9.286801 9.449175 10.494246
H -4.136108 10.158857 5.833887
H -2.660216 10.476751 4.904709
H -10.960262 8.458961 9.184631
H -11.771776 9.763810 10.067236
H -12.951713 9.633878 6.533236
H -13.535854 9.597366 8.218934
H -12.525241 8.277834 7.575439
H -7.550431 13.562997 8.096394
H -7.977421 14.170643 9.703397
H -8.973009 12.928698 8.919703
H -3.225066 8.856676 2.612701
H -2.987936 10.593501 2.314812
H -7.475200 11.951376 10.708445
H -10.660295 7.490068 6.832501
H -6.579748 9.927170 5.823891
H -8.240425 6.572928 2.590362
H -9.617758 3.115693 10.578118
H -7.880643 3.397438 10.525338
H -8.673378 2.527974 9.208063
H -13.482013 4.746382 4.832524
H -15.230439 4.905367 4.576512
H -14.248434 6.336354 4.935627
H -10.394593 5.068719 0.486595
H -11.630270 3.991424 10.515022
H -12.989648 2.997071 9.984277
H -12.900791 4.701018 9.514567
H -14.060090 4.625373 2.393026
H -7.347245 3.527636 4.991599
H -8.002597 1.896663 4.746363
H -8.788506 4.036089 1.813149
H -7.970572 2.524853 2.292303
H -7.333725 4.116946 2.811606
H -10.796592 2.663273 4.585854
H -10.149850 1.804244 3.171018
H -10.961221 2.504250 8.588989
H -9.733624 2.239037 6.386152
H -8.245054 2.875256 7.064400
H -13.175137 3.983520 7.063612
H -13.222140 2.286879 7.560443
H -12.050618 2.826927 6.345790
H -10.987553 3.533858 1.675706
H -12.275786 3.167075 2.810656
H -1.891996 4.962554 2.469297
H -3.103644 3.201770 5.558867
H -2.164228 3.031345 4.074393

H -2.074237 5.399336 4.924051

194
Complex N2 in cavity
U -2.824925 3.340975 3.945113
U -2.722329 7.530911 3.960656
U -2.835230 5.397501 0.330044
P 0.002758 5.947242 -1.007755
P -5.160869 7.762721 6.167789
P -5.372065 1.380633 3.254147
P -5.567545 7.201143 -0.630348
P 0.279645 2.126047 3.608328
P 0.324055 8.109436 4.917387
N -6.186688 5.212377 2.988899
N -1.385669 5.015053 -1.404149
N -4.912950 5.594525 -0.701470
N -3.477403 3.285373 -1.305546
N -5.198390 4.722410 3.063274
N -2.406613 3.837147 1.798566
N -2.757246 5.402433 4.698673
N -2.992110 7.056501 1.835246
N -1.054715 9.038002 4.458790
N -3.119405 10.120830 2.981875
N -2.945708 2.789718 6.708734
N -4.590361 8.602687 4.763226
N -4.634204 2.062628 4.660472
N -1.042625 2.131683 4.737147
C -6.016301 -1.165507 2.245219
C -1.546719 4.176038 -2.574331
C -4.707693 7.623125 -3.287069
C -5.621701 4.553386 -1.425634
C -5.958678 7.728621 -2.420560
C -3.004313 3.713335 -2.631241
C -2.887114 1.999473 -0.928482
C -4.942735 3.201369 -1.217151
C 0.377093 6.934875 -2.595323
C -6.477632 9.169434 -2.409538
C -0.824940 7.811988 -2.934329
C -8.490108 6.688523 -0.858848
C -5.371665 9.058762 7.549897
C -2.216006 1.513567 6.776384
C -4.056732 9.794933 7.785806
C -5.447379 -0.507357 3.505091
C -2.594347 10.410393 1.645814
C -4.579321 10.291542 2.993323
C -4.375269 2.635946 7.014682
C -5.213553 9.831333 4.306197
C -5.098708 1.754569 5.998439
C -5.826048 8.354983 8.831725
C -2.445225 10.954081 3.989920
C -1.019412 10.470282 4.238576
C -4.058411 -1.055738 3.811301
C -8.067350 8.346918 6.044021
C -6.982360 7.308254 5.751845
C -2.347836 3.764845 7.623138
C -7.229708 1.870969 3.386522
C -7.345331 5.951537 6.363386
C -7.807352 2.157969 1.996951
C -7.331600 6.956067 0.104575
C -8.158781 0.948375 4.178840
C 1.519277 4.778653 -0.964140
C 1.147445 3.400779 -0.421246
C 1.653468 7.769842 -2.486565
C 2.623296 5.391008 -0.095815
C -0.863087 1.608360 6.080861
C 0.810254 0.294051 3.418532
C 0.905206 8.771928 6.619293
C 1.730017 2.811420 4.640257
C -0.291755 9.133285 7.493342
C 1.704072 8.779012 3.784635
C -0.410332 -0.619385 3.366307
C 1.349115 8.443352 2.338481
C 3.074181 2.727571 3.916026
C 1.626237 0.128287 2.131410
C 1.760911 7.712515 7.321284
C 1.407377 4.253224 5.024277
C 3.092822 8.250052 4.144042
C -7.668471 8.106473 1.057684
H -6.109429 -0.723423 4.352582
H -6.046011 -2.256333 2.359745
H -7.033394 -0.834107 2.016346
H -4.097409 -2.140378 3.972641
H -1.329085 4.706002 -3.516301
H -0.873259 3.303242 -2.563866
H -4.312159 6.604075 -3.309931
H -4.922438 7.927645 -4.319247
H -3.913771 8.276689 -2.908460
H -6.663079 4.434353 -1.086629

H	-5.687964	4.776044	-2.505060
H	-6.734163	7.071252	-2.832226
H	-3.134664	2.909693	-3.378073
H	-3.620186	4.560755	-2.940473
H	-3.225229	1.726440	0.073196
H	-3.171724	1.203670	-1.638106
H	-1.798202	2.077189	-0.911871
H	-5.346118	2.459523	-1.930482
H	-5.167433	2.839283	-0.206686
H	0.513801	6.194814	-3.396962
H	-5.728510	9.848012	-1.984558
H	-6.693871	9.512061	-3.429345
H	-7.395005	9.285025	-1.824717
H	0.433203	2.878706	-1.061969
H	-1.002216	8.555485	-2.147650
H	-0.658469	8.354054	-3.873595
H	-1.736094	7.218397	-3.040018
H	-8.296163	5.848945	-1.532069
H	-9.400026	6.450336	-0.292358
H	-8.722295	7.561239	-1.478062
H	-1.659766	3.189103	1.513722
H	-2.913083	5.444607	5.712479
H	-3.551604	7.769561	1.346417
H	-6.138569	9.784022	7.249810
H	-2.094016	1.187274	7.825437
H	-2.813295	0.760030	6.258207
H	-3.720435	10.326888	6.892515
H	-4.162069	10.526936	8.596527
H	-3.261885	9.096876	8.069387
H	-3.116243	9.806685	0.900475
H	-2.722604	11.475088	1.385569
H	-1.532987	10.161884	1.591860
H	-4.864421	11.338576	2.782206
H	-4.969685	9.677664	2.173532
H	-4.528553	2.244036	8.037119
H	-4.808370	3.642333	6.982643
H	-6.290918	9.714751	4.110401
H	-5.135073	10.633491	5.060103
H	-6.176155	1.941617	6.131229
H	-4.951331	0.689352	6.248870
H	-5.092249	7.602444	9.143939
H	-5.930232	9.075695	9.652496
H	-6.789064	7.848871	8.717275
H	-2.456400	12.018683	3.693444
H	-3.002796	10.858321	4.924085
H	-5.391078	-0.941765	1.372832
H	-0.376682	10.743907	3.385119
H	-3.625134	-0.595010	4.702576
H	-3.371205	-0.869337	2.979509
H	-7.853061	9.319087	5.591787
H	-9.031678	8.005688	5.645175
H	-8.206631	8.505507	7.118586
H	-2.936153	4.686375	7.621144
H	-2.318276	3.382620	8.657816
H	-7.387476	5.996544	7.457736
H	-8.331241	5.618069	6.014668
H	-6.615189	5.182801	6.093555
H	-7.880146	1.246015	1.393576
H	-8.818660	2.577201	2.074405
H	-7.190621	2.869506	1.440251
H	-7.794486	0.747545	5.189959
H	-9.151066	1.408699	4.273656
H	-8.302876	-0.016000	3.680851
H	-0.104580	-1.661812	3.210698
H	1.884884	4.673706	-1.995341
H	2.043903	2.772722	-0.342662
H	2.550730	7.151520	-2.396854
H	1.777185	8.391302	-3.382452
H	1.617765	8.444292	-1.622905
H	3.527522	4.770029	-0.129056
H	2.903715	6.401326	-0.404484
H	-0.418609	0.599578	6.086289
H	-0.181882	2.234060	6.681333
H	1.433358	0.019636	4.281098
H	1.510522	9.672670	6.445011
H	1.794735	2.201639	5.552342
H	-0.620411	11.029751	5.101012
H	-0.922586	8.253565	7.667601
H	0.043502	9.495835	8.473378
H	-0.921915	9.901087	7.039929
H	-1.331072	4.009857	7.310681
H	1.708208	9.870895	3.910552
H	0.711351	3.469155	0.581524
H	-1.063394	-0.340374	2.530892
H	-1.009864	-0.570464	4.277186
H	0.349578	8.794766	2.068052
H	2.070707	8.893001	1.644968
H	1.358947	7.361151	2.174265

H 3.029120 3.201101 2.928193
H 3.847194 3.250625 4.493301
H 3.413384 1.696377 3.783955
H 2.504195 0.777537 2.090263
H 1.974489 -0.907267 2.026176
H 1.009179 0.360740 1.255969
H 2.624625 7.398956 6.729399
H 2.135443 8.090725 8.281142
H 1.164951 6.816011 7.526234
H 0.440729 4.342949 5.527367
H 2.178521 4.659901 5.690208
H 1.356144 4.898625 4.140863
H 2.298277 5.448198 0.948549
H 3.114764 7.154159 4.157325
H 3.827680 8.581704 3.399513
H 3.436543 8.608806 5.118418
H -6.916978 7.180569 4.663881
H -7.159706 2.822196 3.928872
H -7.179827 6.055472 0.712155
H -6.894513 8.229523 1.820835
H -7.765526 9.060432 0.526173
H -8.620708 7.921597 1.571480

194
Pre-activation of N2
U -2.692784 3.639752 3.836785
U -2.813595 7.266683 3.641930
U -2.775357 5.350736 0.620650
P 0.134506 6.238971 -1.268212
P -5.533685 7.261431 5.960626
P -5.438025 1.511674 3.235388
P -5.543970 7.285018 -0.506839
P 0.488100 1.920551 3.625893
P 0.429321 8.170390 4.985207
N -4.247485 4.417651 2.120566
N -1.227167 5.179538 -1.215592
N -4.730748 5.785913 -0.739915
N -3.407404 3.283896 -1.047658
N -3.937466 5.511806 2.733509
N -1.661313 3.960130 1.752103
N -1.979216 5.525855 4.740073
N -2.109807 7.211822 1.502845
N -1.070523 8.807022 4.426705
N -3.323851 9.939079 3.035309
N -2.832244 3.028794 6.540145
N -4.806210 8.215958 4.731493
N -4.566732 2.235403 4.526545
N -0.896879 2.183585 4.619787
C -6.581819 -1.011697 2.681691
C -1.300168 4.051242 -2.126003
C -4.813003 7.919726 -3.157096
C -5.400496 4.738873 -1.486596
C -6.046865 7.892941 -2.261210
C -2.749527 3.612050 -2.316512
C -2.995101 1.974184 -0.544863
C -4.869823 3.347759 -1.133558
C 0.208024 6.751457 -3.125875
C -6.660422 9.289850 -2.148730
C -1.071858 7.523672 -3.436013
C -8.459118 6.566325 -0.732652
C -5.603746 8.369581 7.533457
C -2.238268 1.695003 6.617048
C -4.193511 8.856522 7.849770
C -5.846273 -0.278634 3.805389
C -2.875687 10.320609 1.699836
C -4.778660 10.069440 3.124258
C -4.272001 3.041304 6.813536
C -5.365783 9.512789 4.421060
C -5.061435 2.124243 5.879097
C -6.166751 7.562673 8.704629
C -2.618463 10.679908 4.080385
C -1.163015 10.239563 4.220681
C -4.549815 -1.007142 4.148275
C -8.356761 8.286611 5.985441
C -7.417386 7.180499 5.500772
C -2.162191 3.977520 7.422644
C -7.193871 2.331293 3.317782
C -7.974006 5.803059 5.871140
C -7.728254 2.557751 1.902160
C -7.275395 6.783456 0.212708
C -8.270343 1.711387 4.211190
C 1.697484 5.116555 -1.175818
C 1.520561 4.078663 -0.068267
C 1.437528 7.584319 -3.487948
C 2.945228 5.954220 -0.873071
C -0.856007 1.652828 5.971736
C 0.685386 0.000216 3.494044
C 0.786174 9.052902 6.673028

C 1.962861 2.300548 4.796191
C -0.506182 9.198293 7.471412
C 1.733493 9.081190 3.905220
C -0.687737 -0.654879 3.364832
C 1.518326 8.655898 2.454029
C 3.317759 1.957068 4.177903
C 1.531526 -0.339900 2.262871
C 1.789964 8.219968 7.476751
C 1.896318 3.780987 5.168797
C 3.173065 8.809216 4.340993
C -7.654179 7.763653 1.325738
H -6.486542 -0.255028 4.697428
H -6.797608 -2.054924 2.956994
H -7.533113 -0.535424 2.423550
H -4.742868 -2.049746 4.441647
H -0.917619 4.282486 -3.135910
H -0.701788 3.187581 -1.782097
H -4.368406 6.925694 -3.249736
H -5.057786 8.288161 -4.164145
H -4.041653 8.576823 -2.738935
H -6.482759 4.689369 -1.280405
H -5.326727 4.893973 -2.582179
H -6.786760 7.209142 -2.697851
H -2.807652 2.753902 -3.016277
H -3.288803 4.450262 -2.765135
H -3.469492 1.791522 0.422122
H -3.283808 1.171213 -1.251169
H -1.915627 1.952464 -0.393113
H -5.254692 2.606394 -1.864016
H -5.243103 3.067417 -0.144249
H 0.225466 5.836310 -3.734710
H -5.938541 9.989171 -1.709295
H -6.948599 9.683471 -3.134770
H -7.552618 9.305536 -1.513764
H 0.646585 3.443934 -0.220702
H -1.091886 8.473356 -2.887093
H -1.151823 7.754089 -4.508120
H -1.954496 6.957846 -3.129665
H -8.247372 5.834781 -1.518397
H -9.331975 6.200339 -0.172137
H -8.767671 7.495871 -1.226347
H -0.680032 3.676108 1.800839
H -1.023285 5.636411 5.094242
H -1.296614 7.600921 1.013961
H -6.248103 9.241296 7.355288
H -2.182448 1.338495 7.666627
H -2.887282 1.017762 6.057311
H -3.763462 9.404049 7.007486
H -4.182268 9.506951 8.736615
H -3.526043 8.009745 8.047070
H -3.365494 9.683166 0.960705
H -3.102169 11.385316 1.489065
H -1.800935 10.161803 1.603056
H -5.092110 11.127463 2.995811
H -5.187018 9.501994 2.280227
H -4.477530 2.767101 7.869417
H -4.605053 4.077095 6.671494
H -6.457815 9.487323 4.252478
H -5.221987 10.252195 5.236420
H -6.118598 2.425367 5.987010
H -5.019247 1.085906 6.269053
H -5.566696 6.659103 8.869386
H -6.152850 8.145855 9.637503
H -7.199239 7.239581 8.534347
H -2.671816 11.773166 3.893638
H -3.124946 10.478660 5.028728
H -5.969868 -1.028707 1.771198
H -0.602725 10.579378 3.328960
H -4.014949 -0.514692 4.964365
H -3.873847 -1.023221 3.285329
H -8.000093 9.285475 5.718936
H -9.358651 8.164998 5.546289
H -8.484358 8.265349 7.074595
H -2.569010 4.974533 7.245119
H -2.288376 3.692413 8.486098
H -8.024498 5.667532 6.959278
H -8.992098 5.663276 5.478755
H -7.344618 5.002543 5.472730
H -7.929093 1.610780 1.383954
H -8.669100 3.127027 1.918168
H -7.004459 3.118904 1.306065
H -7.937395 1.585681 5.245308
H -9.165787 2.350407 4.231110
H -8.592168 0.727653 3.848111
H -0.592618 -1.741558 3.222815
H 1.825374 4.603191 -2.141288
H 2.407909 3.434739 0.005941
H 2.356461 6.990880 -3.490163

H 1.329126 8.018232 -4.492962
H 1.575340 8.417415 -2.786926
H 3.831981 5.310555 -0.780997
H 3.161664 6.707332 -1.635002
H -0.525128 0.597071 6.013667
H -0.143293 2.197748 6.618122
H 1.187364 -0.382522 4.395564
H 1.211998 10.050563 6.485849
H 1.837643 1.696042 5.705766
H -0.742658 10.828848 5.058412
H -0.978662 8.220767 7.619802
H -0.309874 9.630428 8.463925
H -1.239534 9.824468 6.959786
H -1.096200 4.018822 7.194175
H 1.546553 10.161200 3.993277
H 1.386541 4.565885 0.903485
H -1.226634 -0.251804 2.499521
H -1.317587 -0.472734 4.237612
H 0.481981 8.806599 2.140920
H 2.176213 9.213077 1.772283
H 1.729806 7.588330 2.325715
H 3.430885 2.417408 3.188258
H 4.138672 2.330510 4.807283
H 3.460581 0.877412 4.065148
H 2.531709 0.101827 2.295575
H 1.650875 -1.428019 2.150948
H 1.044861 0.036596 1.355326
H 2.735971 8.065473 6.949919
H 2.019218 8.694991 8.442621
H 1.374565 7.226895 7.685329
H 0.917249 4.050515 5.573621
H 2.664269 4.039823 5.911126
H 2.054588 4.416188 4.290003
H 2.822587 6.482591 0.079174
H 3.367518 7.731978 4.418466
H 3.885150 9.217030 3.608568
H 3.407810 9.263483 5.309163
H -7.357773 7.232523 4.406034
H -6.923095 3.311936 3.728503
H -7.027719 5.825353 0.687300
H -6.858546 7.830316 2.072717
H -7.830972 8.773897 0.933336
H -8.573887 7.446382 1.837975

5. References

1. Kahn O. Molecular Magnetism. New York: VCH Publishers, Inc., 1993.

2. Xin X, Douair I and Zhao Y *et al*. Dinitrogen Cleavage by a Multimetallic Cluster Featuring Uranium–Rhodium Bond. *J Am Chem Soc* 2020; **142**: 15004–11.

3. Bergbreiter DE and Killough JM. Reactions of potassium-graphite. *J Am Chem Soc* 1978; **100**: 2126–34.

4. Sheldrick GM. Crystal structure refinement with SHELXL. *Acta Crystallogr Sect C* 2015; **71**: 3–8.

5. Dolomanov OV, Bourhis LJ and Gildea RJ *et al*. OLEX2: A complete structure solution, refinement and analysis program. *J App. Crystallogr* 2009; **42**: 339–41.

6. Becke AD. Density-functional thermochemistry. III. The role of exact exchange. *J Chem Phys* 1993; **98**: 5648–52.

7. Burke K, Perdew JP and Wang Y. In electronic density functional theory: Recent progress and new directions. Dobson JF, Vignale G and Das MP. Eds., New York: Plenum, 1998.

8. Gaussian 09, Revision D.01, Frisch MJ, Trucks GW and Schlegel HB *et al*. Gaussian, Inc., Wallingford CT, 2016.

9. Küchle W, Dolg M and Stoll H *et al*. Energy-adjusted pseudopotentials for the actinides. Parameter sets and test calculations for thorium and thorium monoxide. *J Chem Phys* 1994; **100**: 7535–42.

10. Cao X, Dolg M and Stoll H. Valence basis sets for relativistic energyconsistent small-core actinide pseudopotentials. *J Chem Phys* 2003; **118**: 487–96.

11. Cao X and Dolg M. Segmented contraction scheme for small-core actinide pseudopotential basis set. J Mol Struct: *Theochem* 2004; **673**, 203–9.

12. Höllwarth A, Böhme M and Dapprich S *et al*. A set of d-polarization functions for pseudo-potential basis sets of the main group elements Al-Bi and f-type polarization functions for Zn, Cd, Hg. *Chem Phys Lett* 1993; **208**: 237–240.

13. McLean AD and Chandler GS. Contracted Gaussian basis sets for molecular calculations. I. Second row atoms, Z=11–18. *J Chem Phys*1980; **72**: 5639–48.

14. Hehre WJ, Ditchfield R and Pople JA. Self-consistent molecular orbital methods. XII. Further extensions of Gaussian-type basis sets for use in molecular orbital studies of organic molecules. *J*

Chem Phys 1972; **56**: 2257–61.

15. Grimme S, Ehrlich S and Goerigk L. Effect of the damping function in dispersion corrected desnity functional theory. *J Comp Chem* 2011; **32**: 1456–65.

16. Castro l and Bühl M. Calculations on the one electron dedox potential of oxoiron(IV) porphyrin complexes. *J. Chem. Theory Comput* 2014; **10**: 243–51.

17. Teichteil C and Spiegelmann F. Ab initio molecular calculations including spin-orbit coupling. II. Molecular test on the InH molecule and application to the g states of the Ar_2* excimer. *Chem Phys* 1983; **81**: 283–96.

18. Teichteil C, Pelissier M and Spiegelmann F. Ab initio molecular calculations including spin-orbit coupling. I. Method and atomic tests. *Chem Phys* 1983; **81**: 273–82.
